# Last Glacial Maximum pattern effects reduce climate sensitivity estimates

**DOI:** 10.1126/sciadv.adk9461

**Published:** 2024-04-17

**Authors:** Vincent T. Cooper, Kyle C. Armour, Gregory J. Hakim, Jessica E. Tierney, Matthew B. Osman, Cristian Proistosescu, Yue Dong, Natalie J. Burls, Timothy Andrews, Daniel E. Amrhein, Jiang Zhu, Wenhao Dong, Yi Ming, Philip Chmielowiec

**Affiliations:** ^1^ Department of Atmospheric Sciences, University of Washington, Seattle, WA, USA.; ^2^ School of Oceanography, University of Washington, Seattle, WA, USA.; ^3^ Department of Geosciences, University of Arizona, Tucson, AZ, USA.; ^4^ Department of Geography, University of Cambridge, Cambridge, UK.; ^5^ Department of Climate, Meteorology, and Atmospheric Sciences and Department of Earth Sciences and Environmental Change, University of Illinois at Urbana Champaign, Urbana, IL, USA.; ^6^ Cooperative Institute for Research in Environmental Science, University of Colorado, Boulder, CO, USA.; ^7^ Department of Atmospheric, Oceanic & Earth Sciences, Center for Ocean-Land-Atmosphere Studies, George Mason University, Fairfax, VA, USA.; ^8^ Met Office Hadley Centre, Exeter, UK.; ^9^ Climate and Global Dynamics Laboratory, NSF National Center for Atmospheric Research, Boulder, CO, USA.; ^10^ Cooperative Programs for the Advancement of Earth System Science, University Corporation for Atmospheric Research, Boulder, CO, USA.; ^11^ NOAA/Geophysical Fluid Dynamics Laboratory, Princeton, NJ, USA.; ^12^ Earth and Environmental Sciences and Schiller Institute for Integrated Science and Society, Boston College, Boston, MA, USA.

## Abstract

Here, we show that the Last Glacial Maximum (LGM) provides a stronger constraint on equilibrium climate sensitivity (ECS), the global warming from increasing greenhouse gases, after accounting for temperature patterns. Feedbacks governing ECS depend on spatial patterns of surface temperature (“pattern effects”); hence, using the LGM to constrain future warming requires quantifying how temperature patterns produce different feedbacks during LGM cooling versus modern-day warming. Combining data assimilation reconstructions with atmospheric models, we show that the climate is more sensitive to LGM forcing because ice sheets amplify extratropical cooling where feedbacks are destabilizing. Accounting for LGM pattern effects yields a median modern-day ECS of 2.4°C, 66% range 1.7° to 3.5°C (1.4° to 5.0°C, 5 to 95%), from LGM evidence alone. Combining the LGM with other lines of evidence, the best estimate becomes 2.9°C, 66% range 2.4° to 3.5°C (2.1° to 4.1°C, 5 to 95%), substantially narrowing uncertainty compared to recent assessments.

## Teaser

Ice sheet–driven temperature patterns amplified glacial cooling, implying less future warming than previously estimated.

## INTRODUCTION

Equilibrium climate sensitivity (ECS) is the steady-state response of global mean near-surface air temperature to a doubling of atmospheric CO_2_ from preindustrial levels. ECS is a focus of climate policy and projections because it governs Earth’s long-term response to anthropogenic greenhouse gas changes (*1*, *2*). Recently, the World Climate Research Programme’s 2020 climate sensitivity assessment, hereafter “WCRP20” (*1*), updated the 66% “likely” range for ECS to 2.6° to 3.9°C (2.3° to 4.7°C, 5 to 95%) with a central estimate of 3.1°C, which informed the “likely” range of 2.5° to 4.0°C (2.0° to 5.0°C, “very likely”) and central estimate of 3°C in the Intergovernmental Panel on Climate Change's Sixth Assessment Report (“IPCC AR6”) (*2*). This narrowing of uncertainty compared to previous assessments was achieved by quantitatively combining evidence from process understanding of climate feedbacks, observations over the historical record (1870 to present), and paleoclimate reconstructions of past cold and warm periods. Of these lines of evidence, paleoclimate data from the Last Glacial Maximum (LGM), approximately 21,000 years ago, provide a leading constraint on the upper bound of ECS (*1*–*3*).

Using paleoclimate data to constrain modern-day ECS requires accounting for how climate feedbacks change across different climate states (*1*, *2*, *4*–*9*). The standard assumption is that colder climates are less sensitive (i.e., have more-negative feedbacks) than warmer states (*1*, *2*, *5*–*9*). However, the simple assumption that feedbacks change with global mean temperature does not account for how feedbacks depend on changing spatial patterns of sea-surface temperature (SST), a phenomenon known as the SST “pattern effect” (*10*–*15*).

A robust understanding of the SST pattern effect has been developed in the context of recent warming. Over the past century, SSTs have warmed more in the tropical west Pacific and less in the east Pacific and Southern Ocean (*12*, *16*, *17*). SST changes in tropical regions of deep convection (e.g., the west Pacific) produce strongly negative (stabilizing) feedbacks, whereas SST changes in regions with reflective low clouds (e.g., the east Pacific) or sea ice produce relatively positive (destabilizing) feedbacks (*11*–*15*, *18*). This transient pattern of SST trends is expected to reverse in the future as the tropical east Pacific and Southern Ocean eventually warm at higher rates, producing more-positive feedbacks and a more-sensitive climate at equilibrium (*15*, *19*, *20*). Accounting for this transient pattern effect causes the historical record to become a weak constraint on high values of ECS (*1*, *2*, *16*, *17*, *21*), leaving the LGM as a leading constraint on the ECS upper bound (*1*).

However, pattern effects have not been accounted for in LGM evidence for modern-day ECS (*1*–*3*, *5*, *22*). If the spatial pattern of SST change in equilibrium at the LGM differs from the pattern of future warming, then the climate feedbacks governing climate sensitivity will differ as well. Continental ice sheets are responsible for approximately half of the total LGM forcing (*3*, *23*, *24*) and drive distinct climate responses from changes in topography, albedo, and sea level (*23*, *25*–*30*), suggesting that patterns of SST change at the LGM may differ substantially from those in response to a modern-day doubling of CO_2_. Previous work acknowledged this possibility (*1*, *2*) but did not account for LGM pattern effects because no quantification had yet been made. A key question is, would accounting for LGM pattern effects strengthen or weaken constraints on modern-day ECS?

Here, we quantify the LGM pattern effect and its uncertainty by leveraging two recent advances. First, with the advent of paleoclimate data assimilation (*31*), spatially complete reconstructions of SST and sea ice now exist for the LGM (*3*, *32*–*34*), including estimated uncertainties. Second, recent progress in quantifying pattern effects (*16*, *17*) provides methods using atmospheric general circulation models (AGCMs) to link SST patterns to climate feedbacks. These advances present an opportunity to compare SST changes at the LGM with those expected under anthropogenic CO_2_ forcing and to quantify resulting differences in climate feedbacks and sensitivity. To assess the robustness of our results, we use five AGCMs (sampling uncertainty in how feedbacks relate to SST patterns) and four reconstructions (*3*, *32*–*34*) of the LGM (sampling uncertainty in SST patterns).

### Dependence of modern-day ECS on pattern effects

ECS and climate feedbacks are connected through the standard model of global mean energy balance

ΔN=λΔT+ΔF (1)

where *N* is the top-of-atmosphere radiative imbalance; λ is the net climate feedback (negative for stable climates); *T* is the near-surface air temperature; and *F* is the “effective” radiative forcing, i.e., the change in net downward radiative flux after atmospheric adjustments to imposed perturbations but excluding radiative responses to changing surface temperature (*1*, *2*). Differences (Δ) are relative to an equilibrium reference state, e.g., the preindustrial period. When the forcing is a CO_2_ doubling (2xCO_2_) of preindustrial values, and the climate system reaches equilibrium (Δ*N* = 0), the resulting Δ*T* is referred to as the ECS

$$\text{ECS}=-\Delta F_{2x}/\lambda_{2x}$$ (2)

where Δ*F*_2x_ is the effective radiative forcing (ERF), and λ_2x_ is the net feedback for 2xCO_2_. More-negative values of λ_2x_ indicate a less-sensitive climate (lower ECS).

Here, we aim to quantify the difference in feedbacks (Δλ) operating in the modern climate under 2xCO_2_ (λ_2x_) and at the LGM (λ_LGM_)

$$\Delta \lambda =\lambda_{2x}-\lambda_{\text{LGM}}$$ (3)

Following recent research on pattern effects in the historical record (*1*, *16*, *17*), we estimate λ_2x_ and λ_LGM_ using AGCM simulations with SST and sea-ice concentration (SIC) prescribed as surface boundary conditions. We further evaluate the contributions to Δλ from pattern effects and global mean temperature changes between the LGM and 2xCO_2_.

To infer the modern-day ECS from LGM evidence, Eqs. 2 and 3 can be combined (*1*, *16*) to yield

$$\text{ECS}=\frac{-\Delta F_{2x}}{\lambda_{\mathrm{LGM}}^{*}+\Delta \lambda }$$ (4)

where $\lambda_{\text{LGM}}^{*}$ is the estimate of the unadjusted LGM feedback (determined using Eq. 1 applied to that state), which we take from previous assessments (*1*–*3*), and Δλ is estimated from our AGCM simulations. The value of Δλ depends on spatial patterns of LGM SST and SIC anomalies, for which we use state-of-the-art reconstructions (*3*, *32*–*34*) based on data assimilation.

## RESULTS

### Using data assimilation reconstructions to quantify pattern effects

Similar to Bayesian statistics, paleoclimate data assimilation (*31*) begins with a “prior” estimate of the climate state from model ensembles. Proxy data provide indirect climate observations that update the prior, balancing relative error in the prior and the observations. This results in a “posterior” state estimate, constrained by observations and accounting for uncertainty in priors and data. Since the posterior is sensitive to priors (*35*, *36*), proxies, and methods, we sample this uncertainty by using multiple reconstructions.

**Fig. 1.**
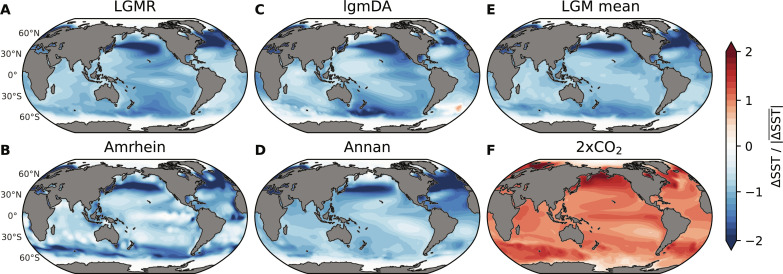
Patterns of SST anomalies from data assimilation at the LGM compared to modern-day doubling of CO_2_ (2xCO_2_). LGM reconstructions include (**A**) LGMR (*32*), (**B**) Amrhein (*34*), (**C**) lgmDA (*3*), (**D**) Annan (*33*), and (**E**) shows the mean of the four LGM patterns. (**F**) Pattern of the multimodel mean from near-equilibrium 2xCO_2_ simulations in LongRunMIP (*39*), initialized from preindustrial control. To show SST patterns, local SST anomalies are divided by absolute values of global mean SST anomalies. All panels show annual means. LGM reconstructions are infilled to modern coastlines (Materials and Methods).

Figure 1 shows the four SST reconstructions (Materials and Methods) we use to quantify the LGM pattern effect. All four reconstructions have a prominent common feature: amplified extratropical cooling in both the North Pacific and North Atlantic Oceans. While the LGM reconstructions differ in other regions that are important for climate feedbacks, e.g., the tropical Pacific (*11*–*15*) and Southern Ocean (*19*, *37*, *38*), their robust agreement in the northern extratropics proves to be essential for the LGM pattern effect. The zonally consistent maximum near 40°N in SST anomalies at the LGM is in strong contrast to the near-equilibrium response to modern-day 2xCO_2_ (Fig. 1F and fig. S1) as simulated by climate models in LongRunMIP (Materials and Methods) (*39*), suggesting the potential for feedbacks to differ between LGM and 2xCO_2_ climates. Using data-constrained patterns to quantify how LGM feedbacks compare to feedbacks in 2xCO_2_ is an advance over past comparisons (all based on models), which have produced conflicting results (text S1) (*22*, *23*, *40*–*44*). While our method overcomes the problem of unconstrained SST patterns from coupled atmosphere-ocean simulations of the LGM, we still rely on AGCMs to estimate feedbacks and their uncertainties.

We calculate net feedbacks using AGCMs with prescribed SST and SIC. We first conduct AGCM simulations with a “baseline” pattern representing the preindustrial climate, for which we use SST and SIC in the Late Holocene (mean of 0 to 4000 years ago) from the LGM Reanalysis (LGMR) (*32*). We then perform AGCM simulations with SST and SIC (Materials and Methods) from 2xCO_2_ in LongRunMIP (*39*) and the four LGM reconstructions (*3*, *32*–*34*) (SST in Fig. 1; SIC in fig. S2). Last, we calculate global mean Δ*N* and Δ*T* in each 2xCO_2_ and LGM simulation relative to the baseline, which yields net feedbacks as λ = Δ*N*/Δ*T* using Eq. 1. All forcings are held constant (Δ*F* = 0) at modern-day levels across our AGCM simulations; therefore, all changes in simulated top-of-atmosphere radiation and feedbacks can be attributed solely to SST/SIC differences (Materials and Methods).

**Fig. 2.**
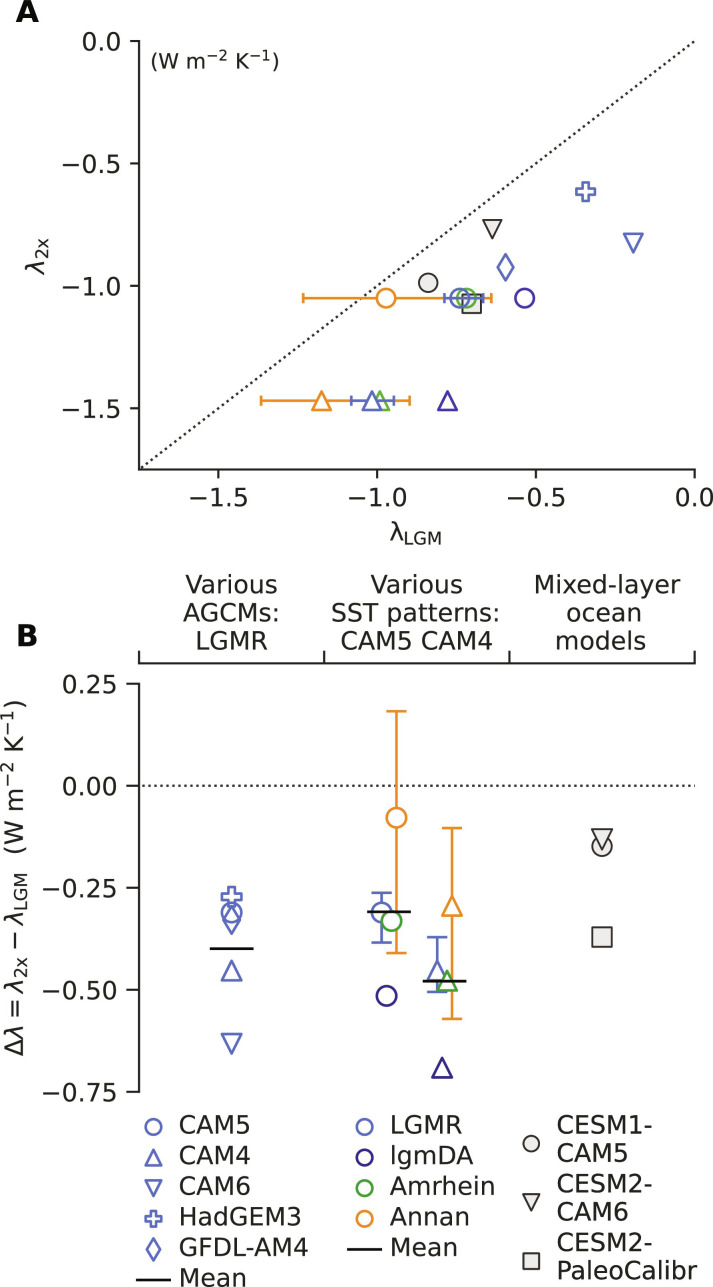
LGM and 2xCO_2_ climate feedbacks and LGM pattern effect (Δλ). Different AGCMs, all using the LGMR pattern for the LGM, are indicated by symbols; different LGM patterns (in CAM5 and CAM4) are indicated by colors. Error bars for Annan and LGMR represent first and fourth quartiles of ensemble members (Materials and Methods); central values indicate ensemble mean. For comparison with AGCM results using LGM data assimilation, the following feedbacks (in a mixed-layer ocean coupled to AGCM) from previous studies are also included: CESM1-CAM5 (*23*), CESM2-CAM6 (*48*), and CESM2-PaleoCalibr (*49*) (modified version of CAM6). (**A**) Scatterplot of 2xCO_2_ feedbacks, λ_2x_, versus LGM feedbacks, λ_LGM_, with λ_2x_ = λ_LGM_ shown as dotted line. (**B**) LGM pattern effect, Δλ = λ_2x_ − λ_LGM_, using feedbacks shown in (A), with Δλ = 0 shown as dotted line. Note that Δλ includes SST pattern effects and contributions from temperature dependence.

We find that λ_2x_ is more negative (stabilizing) than λ_LGM_, indicating that the climate system is more sensitive to LGM forcing than to 2xCO_2_ (Fig. 2). We use the LGMR pattern (Fig. 1A) in five AGCMs (CAM4, CAM5, CAM6, GFDL-AM4, and HadGEM3-GC3.1-LL) to evaluate uncertainty from atmospheric model physics, and we use all four LGM reconstructions (Fig. 1, A to D) in CAM4 and CAM5 to evaluate uncertainty from LGM patterns. This approach is supported by the result that AGCMs tend to reproduce observed relationships between SSTs and top-of-atmosphere radiation when observed SST patterns are prescribed (*45*, *46*). The LGM pattern effect, Δλ in Eq. 3, is negative across all five AGCMs and all four LGM reconstructions. The five AGCMs produce a mean Δλ = −0.40 Wm^−2^ K^–1^ (Fig. 2B; detailed results in tables S1 and S2). We also evaluate uncertainty in the 2xCO_2_ pattern but find that this is of secondary importance (Materials and Methods; figs. S3 and S4). Our main result is that the climate is more sensitive to LGM forcing than it is to modern-day 2xCO_2_ forcing (Δλ < 0), implying lower estimates of modern-day ECS by Eq. 4, and this finding is robust despite uncertainties in atmospheric physics and LGM reconstructions.

## DISCUSSION

### Physical mechanisms driving LGM pattern effects

For comparison with our feedbacks in AGCMs driven by LGM reconstructions, we examine previously published results (*23*) from AGCMs coupled to mixed-layer “slab” oceans (Fig. 2), which allow SST changes in response to imposed forcings but exclude changes in ocean dynamics (*47*). These mixed-layer model versions of CESM1-CAM5 (*23*), CESM2-CAM6 (*48*), and CESM2-PaleoCalibr (*49*) (using a modified CAM6), which differ from our AGCM experiments by including forcings from ice sheets and greenhouse gases, also produce Δλ < 0. Although disagreements in simulated SST patterns compared to proxy data suggest that free-running coupled models cannot reliably estimate the value of Δλ, the coupled models point to mechanisms driving Δλ that are consistent with the reconstructions and our AGCM simulations. In this section, we begin by reviewing simulations in coupled models that demonstrate the physical mechanisms linking patterns of forcing, SST response, and climate feedbacks.

**Fig. 3.**
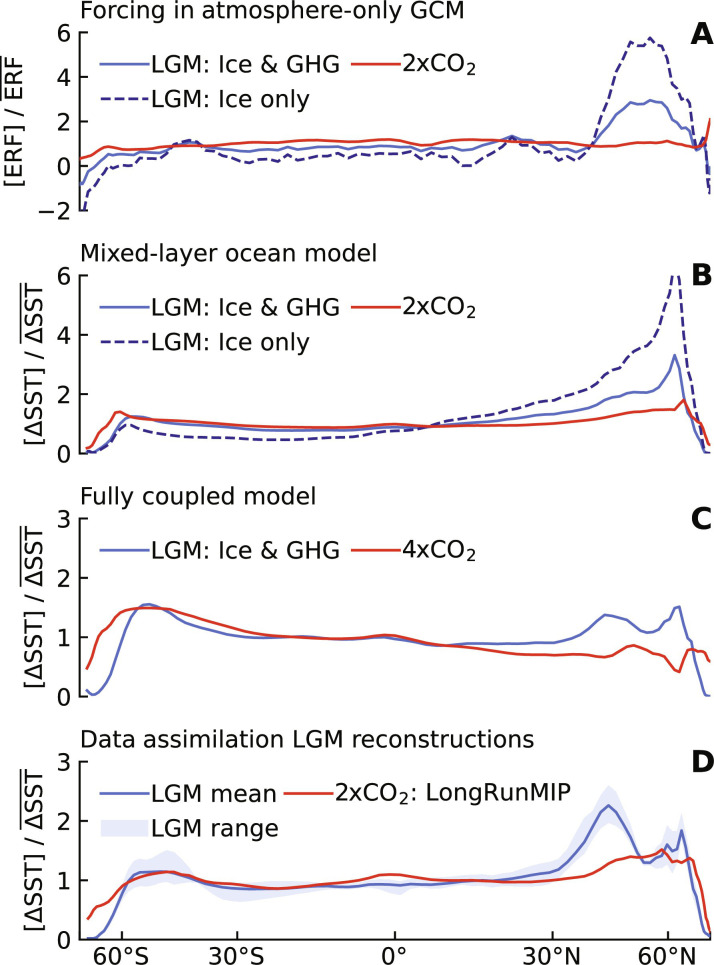
Zonal mean patterns of ERF and SST anomalies. All anomalies are normalized through division by global mean anomalies. (**A** to **C**) Model simulations in CESM1-CAM5 from Zhu and Poulsen (*23*). (A) ERF directly from three fixed-SST simulations using AGCM with LGM greenhouse gas (GHG) and ice-sheet (Ice) forcing, 2xCO_2_, and LGM ice-sheet forcing alone (including coastline changes) (*23*). (B) Equilibrium SST patterns, corresponding to (A), in the coupled mixed-layer ocean model. (C) Quasi-equilibrium SST patterns from fully coupled atmosphere-ocean model, comparing LGM forcings (*23*) with abrupt-4xCO_2_ forcing (*88*); no long-run 2xCO_2_ simulation is available. Note vertical-axis scales. (**D**) Mean and range of SST patterns from four data assimilation reconstructions (*3*, *32*–*34*) of the LGM compared to 2xCO_2_ multimodel mean from LongRunMIP (*39*).

First, we compare zonal mean patterns of ERF and SST changes from CESM1-CAM5 simulations (*23*) under three forcing scenarios: 2xCO_2_ forcing, LGM forcing (ice sheets and greenhouse gases), and LGM ice-sheet forcing alone (including coastline changes). The localized ice-sheet forcing causes the amplified SST response in the northern extratropics at the LGM compared to 2xCO_2_ (Fig. 3, A to C). Explaining the Northern Hemisphere’s response to LGM ice sheets has been a focus of previous studies, which found that amplified SST cooling in the northern extratropics is associated with changes in atmospheric stationary waves, driven by changes in ice-sheet albedo and topography (*23*, *29*, *30*, *50*). Differences in SST responses between LGM and 2xCO_2_ persist at quasi-equilibrium in a fully coupled (atmosphere-ocean GCM) version of CESM1-CAM5 (Fig. 3C and fig. S5). Comparing the fully coupled model’s response (Fig. 3C) to LGM forcing with the data assimilation patterns (Fig. 3D) that we use to quantify pattern effects supports the finding that LGM ice sheets amplify SST cooling in the northern extratropics (*23*, *29*, *30*), but this cooling pattern is more pronounced in proxy reconstructions. The amplified cooling of extratropical SST, driven by ice-sheet forcing, causes the LGM feedback to be less stabilizing than the feedback induced by CO_2_ forcing alone.

Decomposing λ from our AGCM simulations into component feedbacks (fig. S6), including results from direct model output and from radiative kernels (Materials and Methods), shows that shortwave cloud feedbacks are responsible for much of the negative value of Δλ and for much of the spread across AGCMs. The combined feedback from changes in lapse rate and water vapor also contributes to negative values of Δλ. While shortwave clear-sky feedbacks from sea ice and snow are also more positive for the LGM, cloud masking strongly damps the impact of those LGM feedbacks. Accounting for cloud masking (*51*, *52*), feedbacks from surface albedo are more positive in 2xCO_2_, i.e., contribute a positive Δλ, offsetting the negative total Δλ. Overall, our results align with the previous studies focused on the historical record that emphasize cloud and lapse-rate feedbacks in pattern effects (*11*, *13*, *15*, *20*).

Spatial distributions of feedbacks (fig. S7) clarify the connection between ice-sheet forcing, SST response, and cloud feedbacks. Where the SST cooling from LGM ice sheets is amplified in the North Pacific and North Atlantic, positive shortwave cloud feedbacks are prominent because of increases in reflective low clouds (*11*–*15*, *18*, *30*). Compared to 2xCO_2_ simulations, LGM reconstructions have relatively small SST anomalies in tropical ascent regions (fig. S1) where feedbacks are most negative (*11*–*14*, *18*, *37*). However, tropical patterns at the LGM differ across reconstructions, adding to the uncertainty in the LGM pattern effect. Despite these differences in the tropics, all four reconstructions produce a negative pattern effect due to the robust amplification of cooling in the northern extratropics. The role of the northern extratropics illustrates that pattern effects are not always dominated by the tropical Pacific, distinguishing the LGM pattern effect from the well-studied pattern effect of the historical period. In summary, the LGM SST pattern produces a less-negative global climate feedback compared to the 2xCO_2_ SST pattern and Δλ < 0.

### Separating pattern effects from temperature dependence of feedbacks

While our explanation for feedback differences between LGM and 2xCO_2_ forcing focuses on SST pattern differences, we also estimate how Δλ is affected by global mean temperature within our AGCM simulations. Our main AGCM simulations (Fig. 2), which determine our estimate of total Δλ, include not only the impact of SST patterns on feedbacks (pattern effects) but also differences in feedbacks caused by other asymmetries between LGM cooling and modern-day warming under 2xCO_2_ forcing (temperature dependence). We consider that

$$\Delta \lambda \approx \Delta \lambda_{\text{PatternOnly}}+\Delta \lambda_{T}$$ (5)

where Δλ_PatternOnly_ is the feedback change due to different patterns of SST anomalies and Δλ*_T_* is the feedback change due to different global mean temperatures (*T*). Recent community assessments (*1*, *2*) assume that warmer climates are more sensitive (Δλ*_T_* > 0) (*5*–*9*, *41*), which is at odds with the total Δλ < 0 we find for the LGM in AGCMs and coupled models (Fig. 2).

To separate pattern effects from temperature dependence, we perform additional “pattern-only” simulations in CAM4, CAM5, and CAM6 using the LGMR and 2xCO_2_ patterns. For these simulations, we multiply local SST anomalies by constant scaling factors to yield global mean Δ*SST* = −0.5 K with constant baseline SIC (Materials and Methods). SST scaling preserves spatial patterns of anomalies but forces global mean Δ*T* to be small and equal across simulations, i.e., Δλ*_T_* ≈ 0 in the pattern-only simulations. We then repeat the feedback calculations, computing Δλ_PatternOnly_ as in Eq. 3. We estimate the temperature dependence Δλ*_T_* as the residual difference between the main and pattern-only AGCM simulations, rearranging Eq. 5 to Δλ*_T_* ≈ Δλ − Δλ_PatternOnly_ (Materials and Methods). We note that ice-albedo contributions to Δλ could arise from SST patterns or temperature dependence, but our partitioning of Δλ treats sea ice as part of Δλ*_T_*.

The magnitude and sign of Δλ*_T_* is found to be model dependent, in agreement with recent multimodel assessments (*22*, *53*), but Δλ*_T_* appears to be positive and directionally consistent with standard assumptions (*1*, *2*) for feedback temperature dependence. However, Δλ_PatternOnly_ is negative and larger than Δλ*_T_* such that total Δλ < 0 in each AGCM (fig. S8 and table S3). These results suggest that total Δλ for the LGM is mostly attributable to SST pattern effects, and Δλ*_T_* plays a smaller role over this range of climates. Recent assessments (*1*, *2*) considered Δλ*_T_* for the LGM but did not account for the larger, opposing term, Δλ_PatternOnly_. The substantial LGM pattern effect found here motivates revising the LGM evidence for modern-day ECS.

### Climate sensitivity accounting for LGM pattern effects

Constraining modern-day ECS with paleoclimate evidence requires accounting for how forcings and feedbacks differ in paleoclimates relative to the modern-day 2xCO_2_ scenario (*1*, *2*, *5*). LGM inferences of ECS begin with applying Eq. 1 to the LGM in equilibrium, estimating the unadjusted LGM feedback as $\lambda_{\text{LGM}}^{*}=\frac{-\sum \Delta F}{\Delta T}$ . ERFs (Δ*F*) include not only CO_2_ but also ice sheets (including sea level) and, depending on the timescale chosen for ECS (*1*–*3*, *5*), additional changes that have distinct impacts at the LGM: vegetation, dust, N_2_O, and CH_4_ (Materials and Methods). $\lambda_{\text{LGM}}^{*}$ must then be adjusted for differences in feedbacks (Δλ) relative to those operating in modern-day 2xCO_2_, following Eq. 4.

Our results suggest that the LGM feedback is more positive than the 2xCO_2_ feedback because of the LGM ice-sheet forcing and resulting SST pattern. Failing to account for this difference in feedbacks would lead to the inference of higher values of modern-day ECS from the LGM, e.g., (*54*). Some past studies using fully coupled models have considered these feedback differences indirectly by applying an “efficacy” adjustment (*55*) to the LGM forcings. The efficacy framework has led to disparate results for multiple reasons: changes in how forcing is quantified (*40*, *41*, *56*) before ERF became standard (*2*), the lack of data constraints on SST patterns simulated by fully coupled models (*22*, *44*, *57*), and the behavior of intermediate-complexity models with simplified cloud feedbacks (*42*, *43*). Because efficacy is equivalent to the ratio of feedbacks λ_2x_/λ_LGM_ (*58*, *59*), our results could be framed as a median LGM-forcing efficacy of 1.7 (Materials and Methods; tables S1 and S2), consistent with recent studies that find LGM-forcing efficacy greater than 1 using ERF and fully coupled models (*23*, *48*, *49*). However, the pattern effect framework we use replaces the need for forcing efficacy (text S1) (*59*), aligns with modern AGCM methods of quantifying feedbacks (*60*) and ERF (*61*), and incorporates data from the latest reconstructions of the LGM.

To demonstrate the impact of LGM pattern effects, we follow methods in WCRP20 (*1*) and focus on the 150-year timescale of climate sensitivity (*S*) applicable to modern warming (Materials and Methods) (*1*, *2*). We use WCRP20 because that assessment uniquely allows updates of individual parameters and quantitatively combines lines of evidence, but our results would have the same directional impact on other assessments (*2*, *3*). We use forcing values from WCRP20 to estimate the unadjusted LGM feedback, $\lambda_{\text{LGM}}^{*}$ in Eq. 4. However, given emerging evidence (*2*, *3*, *32*, *62*, *63*) after WCRP20, we report results using a global temperature anomaly for the LGM of Δ*T*_LGM_ = −6 ± 1 K in addition to WCRP20’s value of −5 ± 1 K. We implement our key finding by revising the LGM Δλ to now include LGM pattern effects. We assign a normal distribution to Δλ, *N*(μ = −0.37, σ = 0.23) Wm^−2^ K^−1^, reflecting spread across AGCMs and SST reconstructions (Materials and Methods). Our assessment of Δλ and its uncertainty relies on AGCMs to estimate feedbacks from prescribed SST/SIC patterns. We include additional uncertainty tests in figs. S4 and S9, demonstrating that our general conclusions hold if the assumed σ for Δλ is doubled.

**Fig. 4.**
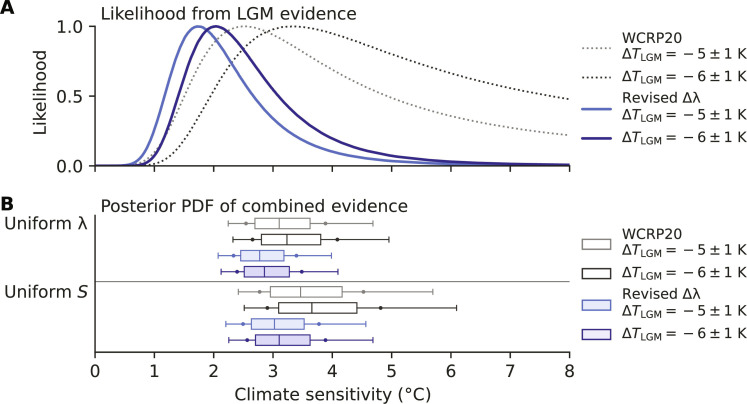
Inference of modern-day climate sensitivity including the LGM pattern effect. Results from WCRP20 (*1*) with no LGM pattern effects and original assumption of Δ*T*_LGM_ ~ *N*(μ = −5, σ = 1) K (gray) and with revised Δ*T*_LGM_ ~ *N*(−6, 1) K (black) based on IPCC AR6 (*2*). Revised climate sensitivity including LGM pattern effects from this study (light and dark blue) assuming Δλ ~ *N*(μ = −0.37, σ = 0.23) Wm^−2^ K^−1^. Climate sensitivity shown is effective sensitivity (*S*) representing 150-year response, as in WCRP20 (*1*). (**A**) Likelihood functions for *S* based on only the LGM line of evidence. (**B**) Posterior probability density function (PDF) after combining LGM with other lines of evidence, assuming a uniform-λ prior (top) or a uniform-*S* prior (bottom). Outlier lines indicate 5th to 95th percentiles, dots indicate 66% “likely” range, and box indicates 25th to 75th percentiles and median.

Accounting for the LGM pattern effect reduces climate sensitivity inferred from the LGM evidence (Fig. 4). With Δ*T*_LGM_ ≈ −6 K, maximum likelihood for *S* from the LGM evidence alone becomes 2.0 K (change of −1.3 K). Assuming a prior that is uniform in *S* from 0 to 20 K (Materials and Methods) for the LGM evidence alone (table S4), we find a posterior median for modern-day ECS of 2.4 K, 66% “likely” range 1.7 to 3.5 K (1.4 to 5.0 K, 5 to 95%). Combining the updated LGM evidence with existing likelihoods for the other lines of evidence (process understanding, historical record, and Pliocene) yields revised Bayesian probability distributions for the two priors in WCRP20: uniform in λ (WCRP20’s “Baseline”) and uniform in *S* (a robustness test).

The impact of the LGM pattern effect on the combined evidence is most pronounced on the upper bound of *S*, which has been notoriously difficult to constrain (*64*). Assuming that Δ*T*_LGM_ ≈ −6 ± 1 K, the median and 66% range from combining lines of evidence for *S* becomes 2.9 K (2.4 to 3.5 K) with a uniform-λ prior or 3.1 K (2.6 to 3.9 K) with a uniform-*S* prior. Corresponding 5 to 95% ranges are 2.1 to 4.1 K with uniform-λ and 2.3 to 4.7 K with uniform-*S*. Accounting for pattern effects in Δλ for the LGM thus reduces the central estimate of modern-day ECS by approximately 0.5 K and reduces the 66% range’s upper bound by 0.6 and 0.9 K for the uniform-λ and uniform-*S* priors, respectively, indicating substantially stronger constraints than WCRP20 (*1*) even after allowing for more glacial cooling. While the qualitative assessment in IPCC AR6 (*2*) cannot be quantitatively updated, these results suggest stronger constraints on modern-day ECS than assessed there, as well.

Accounting for LGM pattern effects—enabled by recent advances in LGM SST reconstruction using paleoclimate data assimilation and in quantifying pattern effects using atmospheric models—provides a tighter upper bound on modern-day ECS. While each line of evidence will surely evolve as scientific understanding improves, the results presented here demonstrate that pattern effects must be accounted for when inferring modern-day climate sensitivity from paleoclimate periods that are substantially affected by non-CO_2_ forcing.

## MATERIALS AND METHODS

### Data assimilation reconstructions of the LGM

We use four LGM reconstructions to quantify the LGM pattern effect, sampling uncertainty across data assimilation methods and model priors (*35*, *36*). Osman *et al.* (*32*) produced the time-dependent “LGMR” spanning the past 24,000 years; the SST and SIC fields that represent the LGM in their reanalysis are time means spanning 19,000 to 23,000 years ago. Tierney *et al.* (*3*) produced the state estimate “lgmDA” dataset. Both the LGMR and lgmDA use priors from isotope-enabled simulations in iCESM1.2 and iCESM1.3 with assimilation of seasonal and annual SST proxies in an ensemble Kalman filter; there are differences in the proxy databases and methods between the two reconstructions. Annan *et al.* (*33*) also used an ensemble Kalman filter but with a multimodel prior, including 19 ensemble members from a wide array of climate models spanning PMIP2 (launched in 2002) to PMIP4 (launched in 2017); they assimilated annual SST proxies and land-temperature proxies; they also applied an adjustment to the prior ensemble to pre-center the prior around available proxy data. Amrhein *et al.* (*34*) fit the MITgcm ocean model to seasonal and annual SST proxies (*65*) using least squares with Lagrange multipliers by adjusting prior atmospheric fields from a CCSM4 LGM simulation (*66*). While these approaches use a diversity of DA methods, versions of CESM1-CAM5 form the prior for two of the reconstructions (*3*, *32*), and the prior covariances could be biased by model errors. Moreover, archived proxy data are geographically inhomogeneous with strong preferences for the NH and tropics; additional data could lead to greater SST agreement across reconstructions outside of the NH.

### Simulations with AGCMs

SST/SIC boundary conditions for the LGM, Late Holocene baseline, and 2xCO_2_ are prepared to maintain constant forcing, i.e., Δ*F* = 0 in Eq. 1, across simulations. Topography is held constant, i.e., the LGM ice sheets are not present in AGCM simulations because their impact is already included as a forcing, and we are isolating feedbacks from changing SST/SIC. For the LGM and Late Holocene datasets, we adjust for differences relative to modern coastlines using kriging and extrapolation in polar regions. Details of sea-level adjustments are provided in text S3.

The 2xCO_2_ SST/SIC is the multimodel mean of 200 years from the end of six 2xCO_2_ simulations, initialized from preindustrial control states, in LongRunMIP (*39*): CESM1.0.4 (years 2300 to 2500), CNRM-CM6-1 (years 550 to 750), HadCM3L (years 500 to 700), MPI-ESM-1.2 (years 800 to 1000), GFDL-ESM2M (years 4300 to 4500), and MIROC3.2 (years 1803 to 2003). These simulations are near equilibrium but only represent an estimate of the true equilibrium SST response to 2xCO_2_.

The Late Holocene, defined as the climatological mean of 0 to 4000 years ago in the LGMR (*32*), is used as the baseline SST/SIC for all feedback calculations. This baseline represents a long-term mean of the preindustrial climate, constrained by assimilation of proxy data. After adjusting for modern sea level, the four LGM boundary conditions and the 2xCO_2_ boundary condition for SST are prepared by adding the SST anomalies from each of the four reconstructions to the Late Holocene baseline SST. Because of nonlinear behavior of sea ice, the LGM and 2xCO_2_ boundary conditions for SIC are not added to the baseline as anomalies but rather are used directly (fig. S2).

We run simulations with the Late Holocene baseline, 2xCO_2_, and LGMR in each of five AGCMs. We run simulations with all four of the LGM reconstructions (LGMR, lgmDA, Amrhein, and Annan) in CAM4 and CAM5, sampling the spread in LGM feedbacks from different reconstructions in two AGCMs that have distinct relationships linking SST patterns to radiative feedbacks based on their respective Green’s functions (*12*, *18*). Spin-up/analysis period/climatological forcing for each AGCM is as follows: 5 years/25 years/2000 for CESM1.2.2.1-CAM4 (*67*), CESM1.2.2.1-CAM5 (*68*), and CESM2.1-CAM6 (*69*) at 1.9° × 2.5° latitude-by-longitude resolution; 5 years/25 years/2014 for HadGEM3-GC3.1-LL (*70*) at N96, ~135-km resolution; and 1 year/30 years/2001 for GFDL-AM4 (*71*) at C96, ~100-km resolution. Parent coupled models of the AGCMs considered here sample a wide range of climate sensitivities, from 2.95 to 5.54 K, and the AGCMs span a wide range of pattern effects in the historical record, from 0.38 to 0.84 Wm^−2^ K^−1^ (*17*).

To compute λ, we take global means over the analysis periods for net top-of-atmosphere radiative imbalance (*N*) and near-surface air temperature (*T*). Differences are taken relative to the Late Holocene baseline, yielding effective feedbacks (*72*) as λ = Δ*N*/Δ*T* for LGM and 2xCO_2_ simulations, given that Δ*F* = 0 in Eq. 1 by design.

To evaluate the impact of uncertainty in the 2xCO_2_ pattern, we also consider existing simulations of abrupt-4xCO_2_ with 150-year regressions (*73*) of Δ*N* versus Δ*T*, denoted as λ_4x(150yr)_, to estimate λ_2x_ (results in figs. S3 and S4 and tables S1 and S2). Results are consistent using either method of estimating λ_2x_. To compute Δλ using λ_4x(150yr)_, we apply a timescale adjustment (ζ) to reconcile feedbacks from equilibrium paleoclimate data with the feedback that applies to 150-year effective sensitivity (*S*), as in WCRP20. We use the central estimate from WCRP20 of ζ = 0.06, and Eq. 3 is modified to Δλ = λ_4x(150yr)_/(1 + ζ) − λ_LGM_.

To investigate how spread across the ensemble members from the two most recent LGM reconstructions affects our results, we run additional simulations using CAM4 and CAM5 with the quartiles of ensemble members that produce the most negative and most positive λ_LGM_ in the LGMR (*32*) and Annan (*33*) reconstructions (error bars in Fig. 2). To determine the SST/SIC boundary conditions for these experiments, ensemble members in each dataset are initially ranked by estimating λ_LGM_ with CAM5 Green’s functions (*18*) applied to SST anomalies from each ensemble member. CAM4 Green’s functions (*12*) produce similar rankings. Green’s functions are only used for ranking and discarded thereafter. We group the ensemble members into quartiles based on rank, and the mean SST/SIC (only SST for the Annan reconstruction) is computed across ensemble members in each quartile. Mean SST anomalies representing the first and fourth quartiles, the most and least negative feedbacks, are used in the additional AGCM simulations. Note that CAM5 with the Annan ensemble’s extreme negative λ_LGM_ produces Δλ > 0. In this quartile, most ensemble members have warming at the LGM over substantial portions of the Southern Ocean (fig. S10). This suggests that Δλ could be positive if the Southern Ocean experienced warming at the LGM, which appears unlikely based on SST proxies (*3*, *32*, *65*), reconstructed deep-ocean temperatures (*74*), and proxy data indicating increased Antarctic sea ice at the LGM (*75*).

### Pattern-only simulations separating pattern and temperature dependence

Feedback differences can be attributed to differences in SST patterns and in global mean near-surface air temperature (*1*) such that Δλ ≈ Δλ_PatternOnly_ + Δλ*_T_*. To separate pattern and temperature impacts on Δλ, we conduct additional pattern-only simulations in CAM4, CAM5, and CAM6 with the LGMR and 2xCO_2_ patterns. For these simulations, we multiply local SST anomalies by constant scale factors, *k*, which are determined for each pattern so that the global mean Δ*SST* is reduced to −0.5 K for both simulations. The constant scale factor for a given pattern of anomalies is calculated from the global mean Δ*SST* as $k=\frac{-0.5\;K}{{\bar{\text{Δ}\text{SST}}}_{\text{global}}}$ , and scaled patterns are then created as Δ*SST*_scaled_ = *k*Δ*SST* at each grid cell. We hold SIC constant at the Late Holocene baseline.

SST scaling preserves the spatial pattern of anomalies but forces global mean Δ*T* to be small enough that feedback changes due to temperature dependence are negligible (Δλ*_T_* ≈ 0). We repeat the feedback calculations, computing Δλ_PatternOnly_ ≈ $\lambda_{2x}^{-0.5K}-\lambda_{\text{LGM}}^{-0.5K}$ as in Eq. 3. While there is no existing method that directly isolates temperature dependence in AGCM simulations, the temperature dependence can be approximated as the residual difference between our main and pattern-only simulations, rearranging Eq. 5 to Δλ*_T_* ≈ Δλ − Δλ_PatternOnly_. In this framework, feedback changes due to sea ice are included in temperature dependence.

We use this pattern-scaling method because it aligns with intuition for pattern effects captured by Green’s functions (*12*, *18*). We do not use Green’s functions to calculate the pattern-only feedbacks, but we briefly discuss the Green’s functions framework here to explain the pattern-only AGCM simulations. In the linear framework of Green’s functions

$$\Delta N=\sum_{j}\frac{\partial N}{\partial SST_{j}}\Delta SST_{j}+\epsilon_{N}$$

$$\Delta T=\sum_{j}\frac{\partial T}{\partial SST_{j}}\Delta SST_{j}+\epsilon_{T}$$

where *j* represents each grid cell, Δ*SST_j_* represents the full SST anomaly at grid cell *j*, ∂*N*/∂*SST_j_* represents the global mean top-of-atmosphere radiative response to a unit increase in local SST at grid cell *j*, ∂*T*/∂*SST_j_* similarly represents the response of global mean near-surface air temperature, and ϵ represents changes that are independent of SST. Because the feedback λ = Δ*N*/Δ*T*, constant scale factors, applied as *k*Δ*SST*, appear in the feedback calculation as λ = (*k*Δ*N*)/(*k*Δ*T*) if ϵ_*N*_ = ϵ_*T*_ = 0 and SST patterns determine λ. In this case, where SST patterns are the sole control on λ, scale factors cancel and have no effect on feedbacks or pattern effects. By comparing feedbacks from scaled pattern-only simulations with feedbacks from simulations with full SST anomalies, we quantify feedback changes that cannot be explained by SST patterns, which we attribute to feedback dependence on global mean temperature. For example, temperature dependence could arise from ∂*N*/∂*SST_j_* changing with global mean temperature or from sea ice appearing at lower latitudes as temperature decreases.

### Feedback decomposition using model fields and radiative kernels

Net λ is calculated from changes in top-of-atmosphere radiation (Δ*N*) divided by changes in global mean temperature (Δ*T*). Δ*N* can be separated into shortwave clear-sky (SWcs), longwave clear-sky (LWcs), and cloud radiative effect (CRE)

$$\Delta N=\Delta N_{\text{SWcs}}+\Delta N_{\text{LWcs}}+\Delta N_{\text{CRE}}$$

Each component of the radiation is available from AGCM output, and dividing all terms by Δ*T* yields feedbacks for each component, which sum to the net feedback. The total clear-sky feedback is the sum of shortwave and longwave components. Because CRE is calculated as all-sky radiation (*N*) minus clear-sky radiation, CRE is affected by changes in noncloud variables.

With radiative kernels (*51*, *76*), feedbacks can be decomposed into contributions from temperature, moisture, and surface albedo. Cloud feedbacks can be estimated by controlling for changes in noncloud variables, and feedbacks from changing surface albedo can be adjusted to account for overlying cloud cover, which we do here following past studies (*51*). Radiative kernels are linearized around a specific climate in a specific model, however, and are prone to errors when applied to different climates and models. We use CAM5 kernels (*77*), convolving them with the monthly mean climatology of anomalies in each AGCM simulation to produce feedbacks in figs. S6 and S7 and zonal means in figs. S12 to S22 (described in text S5). HadGEM3-GC3.1-LL is not included in kernel analysis due to model output limitations. GFDL-AM4’s 2xCO_2_ simulation has error in the kernel-derived clear-sky feedback equal to 15.6% of the actual feedback, exceeding the 15% threshold commonly used as a test of clear-sky linearity (*15*, *76*); all other simulations have clear-sky feedback errors less than 10%. Residuals shown in fig. S6 are based on total (all-sky) radiation: λ_Residual_ = λ_Net_ − Σλ*_j_*, where λ_Net_ is the net feedback from model output and Σλ*_j_* is the sum of each of the following kernel-derived feedbacks: Planck, lapse rate, water vapor, surface albedo, shortwave cloud, and longwave cloud.

### Bayesian estimate of modern-day climate sensitivity

We follow methods (*1*) and code (*78*) provided by WCRP20 for calculating climate sensitivity, but we provide a summary of relevant methods here. ECS is the steady-state change in global mean temperature (*T*) from a doubling of CO_2_, traditionally with ice sheets and vegetation assumed fixed. When inferring climate sensitivity that is relevant to modern warming from paleoclimate evidence, changes in the paleoclimate radiative budget that are distinct from feedback processes in modern-day 2xCO_2_ are treated as forcings; this is typically accomplished by separating “slow” timescale changes as forcings (e.g., ice sheets) from “fast” timescale changes as feedbacks (*5*). WCRP20 applies this framework by focusing on effective climate sensitivity (*S*), i.e., the 150-year system response.

Relative to WCRP20, our key update only affects ∆λ for the LGM. However, given evidence (*2*, *3*, *32*, *62*, *63*) published after WCRP20 showing LGM cooling centered on −6°C instead of −5°C, we report our main results using both assumptions for Δ*T*_LGM_ (Fig. 4 and fig. S4).

To estimate *S*, we use a modified version of WCRP20’s energy balance for the LGM

$$\Delta T_{\text{LGM}}=\frac{-\left(-0.57\Delta F_{2x}+\Delta F\prime \right)}{\frac{\lambda_{2x}}{1+\zeta }-\Delta \lambda }$$ (6)

which determines λ_2x_ and *S* = −Δ*F*_2x_/λ_2x_. We substitute our Δλ, which includes pattern and temperature dependence. Other than testing a colder Δ*T*_LGM_, the parameters are unchanged from WCRP20 with the following normal distributions: modern-day forcing from 2xCO_2_ Δ*F*_2x_ ~ *N*(μ = 4.0, σ = 0.3) Wm^−2^; total non-CO_2_ LGM forcing of Δ*F*′ ~ *N*(−6.15, 2) Wm^−2^ (consisting of −3.2 Wm^−2^ from ice sheets, −1.1 from vegetation, −1.0 from dust aerosols, −0.28 from N_2_O, and −0.57 from CH_4_); the timescale transfer parameter from ECS to the 150-year feedback of ζ ~ *N*(0.06, 0.2); and LGM temperature change Δ*T*_LGM_ ~ *N*(−5, 1) °C, or revised Δ*T*_LGM_ ~ *N*(−6, 1) °C. In WCRP20, Δλ = Δλ*_T_* = −αΔ*T*_LGM_/2, with α ~ *N*(μ = 0.1, σ = 0.1) Wm^−2^ K^−2^.

Quantification of non-CO_2_ ERF from ice sheets (including sea level), dust and other aerosols, vegetation, and other greenhouse gases represents substantial uncertainty. As noted in (*23*), estimates of the ERF for each component of LGM forcing still need to be constrained, and the uncertainty in radiative effects especially due to dust/aerosols (*79*, *80*) and vegetation changes may be underestimated in WCRP20. Future paleoclimate research on dust and other aerosols (*81*–*83*) and vegetation (*84*, *85*) could improve the estimates used here and in paleoclimate modeling (*86*, *87*). Recent assessments (*1*–*3*) discuss how dust and other aerosols, vegetation, and non-CO_2_ greenhouse gases also act as feedbacks on fast timescales, and some studies (*3*, *54*) have calculated a version of climate sensitivity that assumes equivalency in these feedbacks (and in feedbacks from SST patterns) between the LGM and modern-day CO_2_, leading to higher values of ECS (*3*). In the IPCC AR6 (*2*) framework for modern-day ECS, these biogeophysical and non-CO_2_ biogeochemical changes are presented as feedbacks (central value of −0.01 Wm^−2^ K^−1^). However, AR6 does not address how to account for the LGM’s distinct dust/aerosol and vegetation changes when estimating modern-day ECS from LGM evidence, and this accounting should be a topic of future research.

From the AGCM results in this study, we incorporate pattern effects in Δλ of Eq. 6, assigning a revised ∆λ ~ *N*(−0.37, 0.23) Wm^−2^ K^−1^. The revised distribution for ∆λ in our study is based on propagating uncertainty, estimated as spread across AGCMs and LGM reconstructions. To combine uncertainty, we assume that within CAM6, GFDL-AM4, and HadGEM3, the spread in Δλ from different LGM reconstructions would be the same as in CAM4 and CAM5. We add the differences in Δλ from each pattern in CAM4 and CAM5, where differences are computed relative to Δλ using the LGMR pattern, to the results from the remaining three AGCMs. The effect is to treat errors as arising independently in reconstructions and AGCMs. We include Δλ from extreme quartile simulations using ensemble members from Annan and LGMR as part of the combined sample. There are eight simulations from CAM4 and eight from CAM5 that determine spread from LGM patterns. Note that the spread from LGM patterns is similar between CAM4 and CAM5 (Fig. 2).

With the combined sample, we perform bootstrap resampling (described in text S4) with 10^5^ iterations and a sample size of 19 (equal to the number of actual AGCM simulations). The mean over all iterations is $\bar{\Delta \lambda }$ = −0.37 (95% range: −0.47 to −0.26) Wm^−2^ K^−1^, and mean sample standard deviation (SD) = 0.23 (95% range: 0.15 to 0.31) Wm^−2^ K^−1^, which informs our assigned μ and σ, respectively. In fig. S4, we include an uncertainty test by doubling σ to 0.46 Wm^−2^ K^−1^. Using the same bootstrap method, we calculate forcing efficacy (*55*) for the LGM, which is equivalent to the ratio of feedbacks λ_2x_/λ_LGM_, to have a median value of 1.7 (95% range: 1.5 to 2.0), mean value of 2.1 (95% range: 1.6 to 2.6), and sample SD of 1.1 (95% range: 0.6 to 1.4). Efficacy is strongly affected by division of small values of λ_LGM_; hence, CAM6 becomes an outlier in the efficacy calculation. We report the median in the main text to reduce the outlier impact.

Calculations for LGM likelihoods and Bayesian probability density functions (PDFs) for *S* follow the Monte Carlo methods in WCRP20 (*1*, *78*). Likelihoods are independent of the prior, but combining the likelihoods with a prior is required to create posterior PDFs that combine lines of evidence. We show results for both priors in WCRP20: the Uniform(−10, 10) Wm^−2^ K^−1^ prior on λ (their Baseline) and the Uniform(0, 20) °C prior on *S* (robustness test, using a prior that is more conservative regarding the possibility of high climate sensitivity).

## Supplementary Materials

### This PDF file includes:

Supplementary Text Figs. S1 to S22 Tables S1 to S4 References

Correction (26 April 2024):
Due to a production error, the incorrect version of the reference list was attached to the end of the Supplementary Materials PDF. The Supplementary Materials have been updated to include the correct reference list. The original version is available here:

## References

[1] S. C. Sherwood, M. J. Webb, J. D. Annan, K. C. Armour, P. M. Forster, J. C. Hargreaves, G. Hegerl, S. A. Klein, K. D. Marvel, E. J. Rohling, M. Watanabe, T. Andrews, P. Braconnot, C. S. Bretherton, G. L. Foster, Z. Hausfather, A. S. von der Heydt, R. Knutti, T. Mauritsen, J. R. Norris, C. Proistosescu, M. Rugenstein, G. A. Schmidt, K. B. Tokarska, M. D. Zelinka, An assessment of Earth’s climate sensitivity using multiple lines of evidence. Rev. Geophys. 58, e2019RG000678 (2020). 10.1029/2019RG000678 PMC7524012 33015673

[2] P. Forster, T. Storelvmo, K. Armour, W. Collins, J.-L. Dufresne, D. Frame, D. J. Lunt, T. Mauritsen, M. D. Palmer, M. Watanabe, M. Wild, H. Zhang, 2021: The Earth’s energy budget, climate feedbacks, and climate sensitivity, in *Climate Change 2021: The Physical Science Basis. Contribution of Working Group I to the Sixth Assessment Report of the Intergovernmental Panel on Climate Change*, V. Masson-Delmotte, P. Zhai, A. Pirani, S. L. Connors, C. Péan, S. Berger, N. Caud, Y. Chen, L. Goldfarb, M. I. Gomis, M. Huang, K. Leitzell, E. Lonnoy, J. B. R. Matthews, T. K. Maycock, T. Waterfield, O. Yelekçi, R. Yu, B. Zhou, Eds. (Cambridge Univ. Press, 2021).

[3] J. E. Tierney, J. Zhu, J. King, S. B. Malevich, G. J. Hakim, C. J. Poulsen, Glacial cooling and climate sensitivity revisited. Nature 584, 569–573 (2020). 32848226 10.1038/s41586-020-2617-x

[4] S. Manabe, K. Bryan, CO_2_-induced change in a coupled ocean-atmosphere model and its paleoclimatic implications. J. Geophys. Res. 90, 11689–11707 (1985).

[5] PALAEOSENS project members, Making sense of palaeoclimate sensitivity. Nature 491, 683–691 (2012). 23192145 10.1038/nature11574

[6] P. Köhler, B. de Boer, A. S. von der Heydt, L. B. Stap, R. S. W. van de Wal, On the state dependency of the equilibrium climate sensitivity during the last 5 million years. Clim. Past 11, 1801–1823 (2015).

[7] A. S. von der Heydt, H. A. Dijkstra, R. S. W. van de Wal, R. Caballero, M. Crucifix, G. L. Foster, M. Huber, P. Köhler, E. Rohling, P. J. Valdes, P. Ashwin, S. Bathiany, T. Berends, L. G. J. van Bree, P. Ditlevsen, M. Ghil, A. M. Haywood, J. Katzav, G. Lohmann, J. Lohmann, V. Lucarini, A. Marzocchi, H. Pälike, I. R. Baroni, D. Simon, A. Sluijs, L. B. Stap, A. Tantet, J. Viebahn, M. Ziegler, Lessons on climate sensitivity from past climate changes. Curr. Clim. Change Rep. 2, 148–158 (2016). 32025471 10.1007/s40641-016-0049-3 PMC6979625

[8] T. Friedrich, A. Timmermann, M. Tigchelaar, O. E. Timm, A. Ganopolski, Nonlinear climate sensitivity and its implications for future greenhouse warming. Sci. Adv. 2, e1501923 (2016). 28861462 10.1126/sciadv.1501923 PMC5569956

[9] E. J. Rohling, G. Marino, G. L. Foster, P. A. Goodwin, A. S. von der Heydt, P. Köhler, Comparing climate sensitivity, past and present. Ann. Rev. Mar. Sci. 10, 261–288 (2018). 10.1146/annurev-marine-121916-063242 28938079

[10] K. C. Armour, C. M. Bitz, G. H. Roe, Time-varying climate sensitivity from regional feedbacks. J. Clim. 26, 4518–4534 (2013).

[11] C. Zhou, M. D. Zelinka, S. A. Klein, Impact of decadal cloud variations on the Earth’s energy budget. Nat. Geosci. 9, 871–874 (2016).

[12] Y. Dong, C. Proistosescu, K. C. Armour, D. S. Battisti, Attributing historical and future evolution of radiative feedbacks to regional warming patterns using a Green’s function approach: The preeminence of the western Pacific. J. Clim. 32, 5471–5491 (2019).

[13] T. Andrews, M. J. Webb, The dependence of global cloud and lapse rate feedbacks on the spatial structure of tropical Pacific warming. J. Clim. 31, 641–654 (2018).

[14] S. Fueglistaler, Observational evidence for two modes of coupling between sea surface temperatures, tropospheric temperature profile, and shortwave cloud radiative effect in the tropics. Geophys. Res. Lett. 46, 9890–9898 (2019).

[15] P. Ceppi, J. M. Gregory, Relationship of tropospheric stability to climate sensitivity and Earth’s observed radiation budget. Proc. Natl. Acad. Sci. U.S.A. 114, 13126–13131 (2017). 29183969 10.1073/pnas.1714308114 PMC5740654

[16] T. Andrews, J. M. Gregory, D. Paynter, L. G. Silvers, C. Zhou, T. Mauritsen, M. J. Webb, K. C. Armour, P. M. Forster, H. Titchner, Accounting for changing temperature patterns increases historical estimates of climate sensitivity. Geophys. Res. Lett. 45, 8490–8499 (2018).

[17] T. Andrews, A. Bodas-Salcedo, J. M. Gregory, Y. Dong, K. C. Armour, D. Paynter, P. Lin, A. Modak, T. Mauritsen, J. N. S. Cole, B. Medeiros, J. J. Benedict, H. Douville, R. Roehrig, T. Koshiro, H. Kawai, T. Ogura, J.-L. Dufresne, R. P. Allan, C. Liu, On the effect of historical SST patterns on radiative feedback. J. Geophys. Res. Atmos. 127, e2022JD036675 (2022).

[18] C. Zhou, M. D. Zelinka, S. A. Klein, Analyzing the dependence of global cloud feedback on the spatial pattern of sea surface temperature change with a Green’s function approach. J. Adv. Model. Earth Syst. 9, 2174–2189 (2017).

[19] K. C. Armour, J. Marshall, J. R. Scott, A. Donohoe, E. R. Newsom, Southern Ocean warming delayed by circumpolar upwelling and equatorward transport. Nat. Geosci. 9, 549–554 (2016).

[20] Y. Dong, K. C. Armour, M. D. Zelinka, C. Proistosescu, D. S. Battisti, C. Zhou, T. Andrews, Intermodel spread in the pattern effect and its contribution to climate sensitivity in CMIP5 and CMIP6 models. J. Clim. 33, 7755–7775 (2020).

[21] C. Proistosescu, P. J. Huybers, Slow climate mode reconciles historical and model-based estimates of climate sensitivity. Sci. Adv. 3, e1602821 (2017). 28695203 10.1126/sciadv.1602821 PMC5498107

[22] M. Renoult, N. Sagoo, J. Zhu, T. Mauritsen, Causes of the weak emergent constraint on climate sensitivity at the Last Glacial Maximum. Clim. Past 19, 323–356 (2023).

[23] J. Zhu, C. J. Poulsen, Last Glacial Maximum (LGM) climate forcing and ocean dynamical feedback and their implications for estimating climate sensitivity. Clim Past 17, 253–267 (2021).

[24] P. Braconnot, M. Kageyama, Shortwave forcing and feedbacks in Last Glacial Maximum and Mid-Holocene PMIP3 simulations. Philos. Trans. R. Soc. A Math. Phys. Eng. Sci. 373, 20140424 (2015). 10.1098/rsta.2014.0424 26438281

[25] S. Manabe, A. J. Broccoli, The influence of continental ice sheets on the climate of an ice age. J. Geophys. Res. 90, 2167–2190 (1985).

[26] K. H. Cook, I. M. Held, Stationary waves of the ice age climate. J. Clim. 1, 807–819 (1988).

[27] S.-Y. Lee, J. C. H. Chiang, P. Chang, Tropical Pacific response to continental ice sheet topography. Clim. Dyn. 44, 2429–2446 (2015).

[28] P. N. DiNezio, J. E. Tierney, B. L. Otto-Bliesner, A. Timmermann, T. Bhattacharya, N. Rosenbloom, E. Brady, Glacial changes in tropical climate amplified by the Indian Ocean. Sci. Adv. 4, eaat9658 (2018). 30547084 10.1126/sciadv.aat9658 PMC6291310

[29] W. H. G. Roberts, C. Li, P. J. Valdes, The mechanisms that determine the response of the Northern Hemisphere’s stationary waves to North American ice sheets. J. Clim. 32, 3917–3940 (2019).

[30] D. J. Amaya, A. M. Seltzer, K. B. Karnauskas, J. M. Lora, X. Zhang, P. N. DiNezio, Air-sea coupling shapes North American hydroclimate response to ice sheets during the Last Glacial Maximum. Earth Planet. Sci. Lett. 578, 117271 (2022).

[31] G. J. Hakim, J. Emile-Geay, E. J. Steig, D. Noone, D. M. Anderson, R. Tardif, N. Steiger, W. A. Perkins, The last millennium climate reanalysis project: Framework and first results. J. Geophys. Res. Atmos. 121, 6745–6764 (2016).

[32] M. B. Osman, J. E. Tierney, J. Zhu, R. Tardif, G. J. Hakim, J. King, C. J. Poulsen, Globally resolved surface temperatures since the Last Glacial Maximum. Nature 599, 239–244 (2021). 34759364 10.1038/s41586-021-03984-4

[33] J. D. Annan, J. C. Hargreaves, T. Mauritsen, A new global surface temperature reconstruction for the Last Glacial Maximum. Clim. Past 18, 1883–1896 (2022).

[34] D. E. Amrhein, C. Wunsch, O. Marchal, G. Forget, A global Glacial Ocean state estimate constrained by upper-ocean temperature proxies. J. Clim. 31, 8059–8079 (2018).

[35] D. E. Amrhein, G. J. Hakim, L. A. Parsons, Quantifying structural uncertainty in paleoclimate data assimilation with an application to the Last Millennium. Geophys. Res. Lett. 47, e2020GL090485 (2020).

[36] L. A. Parsons, D. E. Amrhein, S. C. Sanchez, R. Tardif, M. K. Brennan, G. J. Hakim, Do multi-model ensembles improve reconstruction skill in paleoclimate data assimilation? Earth Space Sci. 8, e2020EA001467 (2021).

[37] S. M. Kang, S.-P. Xie, Dependence of climate response on meridional structure of external thermal forcing. J. Clim. 27, 5593–5600 (2014).

[38] B. E. J. Rose, K. C. Armour, D. S. Battisti, N. Feldl, D. D. B. Koll, The dependence of transient climate sensitivity and radiative feedbacks on the spatial pattern of ocean heat uptake. Geophys. Res. Lett. 41, 1071–1078 (2014).

[39] M. Rugenstein, J. Bloch-Johnson, A. Abe-Ouchi, T. Andrews, U. Beyerle, L. Cao, T. Chadha, G. Danabasoglu, J.-L. Dufresne, L. Duan, M.-A. Foujols, T. Frölicher, O. Geoffroy, J. Gregory, R. Knutti, C. Li, A. Marzocchi, T. Mauritsen, M. Menary, E. Moyer, L. Nazarenko, D. Paynter, D. Saint-Martin, G. A. Schmidt, A. Yamamoto, S. Yang, LongRunMIP: Motivation and design for a large collection of millennial-length AOGCM simulations. Bull. Am. Meteorol. Soc. 100, 2551–2570 (2019).

[40] M. Crucifix, Does the Last Glacial Maximum constrain climate sensitivity? Geophys. Res. Lett. 33, L18701 (2006).

[41] M. Yoshimori, J. C. Hargreaves, J. D. Annan, T. Yokohata, A. Abe-Ouchi, Dependency of feedbacks on forcing and climate state in physics parameter ensembles. J. Clim. 24, 6440–6455 (2011).

[42] L. B. Stap, P. Köhler, G. Lohmann, Including the efficacy of land ice changes in deriving climate sensitivity from paleodata. Earth Syst. Dynam. 10, 333–345 (2019).

[43] J. D. Shakun, Modest global-scale cooling despite extensive early Pleistocene ice sheets. Quat. Sci. Rev. 165, 25–30 (2017).

[44] P. O. Hopcroft, P. J. Valdes, How well do simulated Last Glacial Maximum tropical temperatures constrain equilibrium climate sensitivity? Geophys. Res. Lett. 42, 5533–5539 (2015).

[45] R. P. Allan, C. Liu, N. G. Loeb, M. D. Palmer, M. Roberts, D. Smith, P.-L. Vidale, Changes in global net radiative imbalance 1985–2012. Geophys. Res. Lett. 41, 5588–5597 (2014). 25821270 10.1002/2014GL060962 PMC4373161

[46] N. G. Loeb, H. Wang, R. P. Allan, T. Andrews, K. Armour, J. N. S. Cole, J.-L. Dufresne, P. Forster, A. Gettelman, H. Guo, T. Mauritsen, Y. Ming, D. Paynter, C. Proistosescu, M. F. Stuecker, U. Willén, K. Wyser, New generation of climate models track recent unprecedented changes in Earth’s radiation budget observed by CERES. Geophys. Res. Lett. 47, e2019GL086705 (2020).

[47] C. M. Bitz, K. M. Shell, P. R. Gent, D. A. Bailey, G. Danabasoglu, K. C. Armour, M. M. Holland, J. T. Kiehl, Climate sensitivity of the Community Climate System Model, version 4. J. Clim. 25, 3053–3070 (2012).

[48] J. Zhu, B. L. Otto-Bliesner, E. C. Brady, C. J. Poulsen, J. E. Tierney, M. Lofverstrom, P. DiNezio, Assessment of equilibrium climate sensitivity of the community Earth System Model version 2 through simulation of the Last Glacial Maximum. Geophys. Res. Lett. 48, e2020GL091220 (2021).

[49] J. Zhu, B. L. Otto-Bliesner, E. C. Brady, A. Gettelman, J. T. Bacmeister, R. B. Neale, C. J. Poulsen, J. K. Shaw, Z. S. McGraw, J. E. Kay, LGM paleoclimate constraints inform cloud parameterizations and equilibrium climate sensitivity in CESM2. J. Adv. Model. Earth Syst. 14, e2021MS002776 (2022).

[50] G. H. Roe, R. S. Lindzen, The mutual interaction between continental-scale ice sheets and atmospheric stationary waves. J. Clim. 14, 1450–1465 (2001).

[51] B. J. Soden, I. M. Held, R. Colman, K. M. Shell, J. T. Kiehl, C. A. Shields, Quantifying climate feedbacks using radiative kernels. J. Clim. 21, 3504–3520 (2008).

[52] S. P. Raghuraman, D. Paynter, R. Menzel, V. Ramaswamy, Forcing, cloud feedbacks, cloud masking, and internal variability in the cloud radiative effect satellite record. J. Clim. 36, 4151–4167 (2023).

[53] J. Bloch-Johnson, M. Rugenstein, M. B. Stolpe, T. Rohrschneider, Y. Zheng, J. M. Gregory, Climate sensitivity increases under higher CO_2_ levels due to feedback temperature dependence. Geophys. Res. Lett. 48, e2020GL089074 (2021).

[54] J. E. Hansen, M. Sato, L. Simons, L. S. Nazarenko, I. Sangha, P. Kharecha, J. C. Zachos, K. von Schuckmann, N. G. Loeb, M. B. Osman, Q. Jin, G. Tselioudis, E. Jeong, A. Lacis, R. Ruedy, G. Russell, J. Cao, J. Li, Global warming in the pipeline. Oxford Open Clim. Change 3, kgad008 (2023).

[55] J. Hansen, M. Sato, R. Ruedy, L. Nazarenko, A. Lacis, G. A. Schmidt, G. Russell, I. Aleinov, M. Bauer, S. Bauer, N. Bell, B. Cairns, V. Canuto, M. Chandler, Y. Cheng, A. Del Genio, G. Faluvegi, E. Fleming, A. Friend, T. Hall, C. Jackman, M. Kelley, N. Kiang, D. Koch, J. Lean, J. Lerner, K. Lo, S. Menon, R. Miller, P. Minnis, T. Novakov, V. Oinas, J. Perlwitz, J. Perlwitz, D. Rind, A. Romanou, D. Shindell, P. Stone, S. Sun, N. Tausnev, D. Thresher, B. Wielicki, T. Wong, M. Yao, S. Zhang, Efficacy of climate forcings. J. Geophys. Res. Atmos. 110, D18104 (2005).

[56] M. Yoshimori, T. Yokohata, A. Abe-Ouchi, A comparison of climate feedback strength between CO_2_ doubling and LGM experiments. J. Clim. 22, 3374–3395 (2009).

[57] M. Kageyama, S. P. Harrison, M.-L. Kapsch, M. Lofverstrom, J. M. Lora, U. Mikolajewicz, S. Sherriff-Tadano, T. Vadsaria, A. Abe-Ouchi, N. Bouttes, D. Chandan, L. J. Gregoire, R. F. Ivanovic, K. Izumi, A. N. LeGrande, F. Lhardy, G. Lohmann, P. A. Morozova, R. Ohgaito, A. Paul, W. R. Peltier, C. J. Poulsen, A. Quiquet, D. M. Roche, X. Shi, J. E. Tierney, P. J. Valdes, E. Volodin, J. Zhu, The PMIP4 Last Glacial Maximum experiments: Preliminary results and comparison with the PMIP3 simulations. Clim. Past 17, 1065–1089 (2021).

[58] T. B. Richardson, P. M. Forster, C. J. Smith, A. C. Maycock, T. Wood, T. Andrews, O. Boucher, G. Faluvegi, D. Fläschner, Ø. Hodnebrog, M. Kasoar, A. Kirkevåg, J.-F. Lamarque, J. Mülmenstädt, G. Myhre, D. Olivié, R. W. Portmann, B. H. Samset, D. Shawki, D. Shindell, P. Stier, T. Takemura, A. Voulgarakis, D. Watson-Parris, Efficacy of climate forcings in PDRMIP models. J. Geophys. Res. Atmos. 124, 12824–12844 (2019). 32025453 10.1029/2019JD030581 PMC6988499

[59] C. Zhou, M. Wang, M. D. Zelinka, Y. Liu, Y. Dong, K. C. Armour, Explaining forcing efficacy with pattern effect and state dependence. Geophys. Res. Lett. 50, e2022GL101700 (2023).

[60] M. J. Webb, T. Andrews, A. Bodas-Salcedo, S. Bony, C. S. Bretherton, R. Chadwick, H. Chepfer, H. Douville, P. Good, J. E. Kay, S. A. Klein, R. Marchand, B. Medeiros, A. P. Siebesma, C. B. Skinner, B. Stevens, G. Tselioudis, Y. Tsushima, M. Watanabe, The Cloud Feedback Model Intercomparison Project (CFMIP) contribution to CMIP6. Geosci. Model Dev. 10, 359–384 (2017).

[61] R. Pincus, P. M. Forster, B. Stevens, The radiative forcing model intercomparison project (RFMIP): Experimental protocol for CMIP6. Geosci. Model Dev. 9, 3447–3460 (2016).

[62] A. M. Seltzer, J. Ng, W. Aeschbach, R. Kipfer, J. T. Kulongoski, J. P. Severinghaus, M. Stute, Widespread six degrees celsius cooling on land during the Last Glacial Maximum. Nature 593, 228–232 (2021). 33981051 10.1038/s41586-021-03467-6

[63] Z. Liu, Y. Bao, L. G. Thompson, E. Mosley-Thompson, C. Tabor, G. J. Zhang, M. Yan, M. Lofverstrom, I. Montanez, J. Oster, Tropical mountain ice core δ^18^O: A Goldilocks indicator for global temperature change. Sci. Adv. 9, eadi6725 (2023). 37939192 10.1126/sciadv.adi6725 PMC10631737

[64] R. Knutti, G. C. Hegerl, The equilibrium sensitivity of the Earth’s temperature to radiation changes. Nat. Geosci. 1, 735–743 (2008).

[65] MARGO Project Members, Constraints on the magnitude and patterns of ocean cooling at the Last Glacial Maximum. Nat. Geosci. 2, 127–132 (2009).

[66] E. C. Brady, B. L. Otto-Bliesner, J. E. Kay, N. Rosenbloom, Sensitivity to glacial forcing in the CCSM4. J. Clim. 26, 1901–1925 (2013).

[67] R. B. Neale, J. Richter, S. Park, P. H. Lauritzen, S. J. Vavrus, P. J. Rasch, M. Zhang, The mean climate of the Community Atmosphere Model (CAM4) in forced SST and fully coupled experiments. J. Clim. 26, 5150–5168 (2013).

[68] R. B. Neale, A. Gettelman, S. Park, C.-C. Chen, P. H. Lauritzen, D. L. Williamson, A. J. Conley, D. Kinnison, D. Marsh, A. K. Smith, F. Vitt, R. Garcia, J.-F. Lamarque, M. Mills, S. Tilmes, H. Morrison, P. Cameron-Smith, W. D. Collins, M. J. Iacono, R. C. Easter, X. Liu, S. J. Ghan, P. J. Rasch, M. A. Taylor, Description of the NCAR Community Atmosphere Model (CAM 5.0) (NCAR/TN-486+STR) (2012); 10.5065/wgtk-4g06.

[69] G. Danabasoglu, J.-F. Lamarque, J. Bacmeister, D. A. Bailey, A. K. DuVivier, J. Edwards, L. K. Emmons, J. Fasullo, R. Garcia, A. Gettelman, C. Hannay, M. M. Holland, W. G. Large, P. H. Lauritzen, D. M. Lawrence, J. T. M. Lenaerts, K. Lindsay, W. H. Lipscomb, M. J. Mills, R. Neale, K. W. Oleson, B. Otto-Bliesner, A. S. Phillips, W. Sacks, S. Tilmes, L. Kampenhout, M. Vertenstein, A. Bertini, J. Dennis, C. Deser, C. Fischer, B. Fox-Kemper, J. E. Kay, D. Kinnison, P. J. Kushner, V. E. Larson, M. C. Long, S. Mickelson, J. K. Moore, E. Nienhouse, L. Polvani, P. J. Rasch, W. G. Strand, The Community Earth System Model Version 2 (CESM2). J. Adv. Model. Earth Syst. 12, e2019MS001916 (2020).

[70] K. D. Williams, D. Copsey, E. W. Blockley, A. Bodas-Salcedo, D. Calvert, R. Comer, P. Davis, T. Graham, H. T. Hewitt, R. Hill, P. Hyder, S. Ineson, T. C. Johns, A. B. Keen, R. W. Lee, A. Megann, S. F. Milton, J. G. L. Rae, M. J. Roberts, A. A. Scaife, R. Schiemann, D. Storkey, L. Thorpe, I. G. Watterson, D. N. Walters, A. West, R. A. Wood, T. Woollings, P. K. Xavier, The Met Office Global Coupled model 3.0 and 3.1 (GC3.0 and GC3.1) configurations. J. Adv. Model. Earth Syst. 10, 357–380 (2017).

[71] I. M. Held, H. Guo, A. Adcroft, J. P. Dunne, L. W. Horowitz, J. Krasting, E. Shevliakova, M. Winton, M. Zhao, M. Bushuk, A. T. Wittenberg, B. Wyman, B. Xiang, R. Zhang, W. Anderson, V. Balaji, L. Donner, K. Dunne, J. Durachta, P. P. G. Gauthier, P. Ginoux, J.-C. Golaz, S. M. Griffies, R. Hallberg, L. Harris, M. Harrison, W. Hurlin, J. John, P. Lin, S.-J. Lin, S. Malyshev, R. Menzel, P. C. D. Milly, Y. Ming, V. Naik, D. Paynter, F. Paulot, V. Ramaswamy, B. Reichl, T. Robinson, A. Rosati, C. Seman, L. G. Silvers, S. Underwood, N. Zadeh, Structure and performance of GFDL’s CM4.0 climate model. J. Adv. Model. Earth Syst. 11, 3691–3727 (2019).

[72] M. A. A. Rugenstein, K. C. Armour, Three flavors of radiative feedbacks and their implications for estimating equilibrium climate sensitivity. Geophys. Res. Lett. 48, e2021GL092983 (2021).

[73] J. M. Gregory, W. J. Ingram, M. A. Palmer, G. S. Jones, P. A. Stott, R. B. Thorpe, J. A. Lowe, T. C. Johns, K. D. Williams, A new method for diagnosing radiative forcing and climate sensitivity. Geophys. Res. Lett. 31, L03205 (2004).

[74] J. F. Adkins, K. McIntyre, D. P. Schrag, The salinity, temperature, and δ^18^O of the glacial deep ocean. Science 298, 1769–1773 (2002). 12459585 10.1126/science.1076252

[75] R. A. Green, L. Menviel, K. J. Meissner, X. Crosta, D. Chandan, G. Lohmann, W. R. Peltier, X. Shi, J. Zhu, Evaluating seasonal sea-ice cover over the Southern Ocean at the Last Glacial Maximum. Clim. Past 18, 845–862 (2022).

[76] K. M. Shell, J. T. Kiehl, C. A. Shields, Using the radiative kernel technique to calculate climate feedbacks in NCAR’s Community Atmospheric Model. J. Clim. 21, 2269–2282 (2008).

[77] A. G. Pendergrass, apendergrass/cam5-kernels (2019); 10.5281/zenodo.3359041.

[78] M. Webb, Code and Data for WCRP Climate Sensitivity Assessment (2020); 10.5281/zenodo.3945275.

[79] J. F. Kok, T. Storelvmo, V. A. Karydis, A. A. Adebiyi, N. M. Mahowald, A. T. Evan, C. He, D. M. Leung, Mineral dust aerosol impacts on global climate and climate change. Nat. Rev. Earth Environ. 4, 71–86 (2023).

[80] N. M. Mahowald, L. Li, S. Albani, D. S. Hamilton, J. F. Kok, Opinion: The importance of historical and paleoclimate aerosol radiative effects. Atmos. Chem. Phys. 24, 533–551 (2024).

[81] N. Sagoo, T. Storelvmo, Testing the sensitivity of past climates to the indirect effects of dust. Geophys. Res. Lett. 44, 5807–5817 (2017).

[82] S. Albani, N. M. Mahowald, Paleodust insights into dust impacts on climate. J. Clim. 32, 7897–7913 (2019).

[83] S. Albani, Y. Balkanski, N. Mahowald, G. Winckler, V. Maggi, B. Delmonte, Aerosol-climate interactions during the Last Glacial Maximum. Curr. Clim. Change Rep. 4, 99–114 (2018).

[84] I. C. Prentice, S. P. Harrison, P. J. Bartlein, Global vegetation and terrestrial carbon cycle changes after the last ice age. New Phytol. 189, 988–998 (2011). 21288244 10.1111/j.1469-8137.2010.03620.x

[85] P. J. Bartlein, S. P. Harrison, S. Brewer, S. Connor, B. A. S. Davis, K. Gajewski, J. Guiot, T. I. Harrison-Prentice, A. Henderson, O. Peyron, I. C. Prentice, M. Scholze, H. Seppä, B. Shuman, S. Sugita, R. S. Thompson, A. E. Viau, J. Williams, H. Wu, Pollen-based continental climate reconstructions at 6 and 21 ka: A global synthesis. Clim. Dyn. 37, 775–802 (2011).

[86] M. Kageyama, S. Albani, P. Braconnot, S. P. Harrison, P. O. Hopcroft, R. F. Ivanovic, F. Lambert, O. Marti, W. R. Peltier, J.-Y. Peterschmitt, D. M. Roche, L. Tarasov, X. Zhang, E. C. Brady, A. M. Haywood, A. N. LeGrande, D. J. Lunt, N. M. Mahowald, U. Mikolajewicz, K. H. Nisancioglu, B. L. Otto-Bliesner, H. Renssen, R. A. Tomas, Q. Zhang, A. Abe-Ouchi, P. J. Bartlein, J. Cao, Q. Li, G. Lohmann, R. Ohgaito, X. Shi, E. Volodin, K. Yoshida, X. Zhang, W. Zheng, The PMIP4 contribution to CMIP6—Part 4: Scientific objectives and experimental design of the PMIP4-CMIP6 Last Glacial Maximum experiments and PMIP4 sensitivity experiments. Geosci. Model Dev. 10, 4035–4055 (2017).

[87] G. A. Schmidt, J. D. Annan, P. J. Bartlein, B. I. Cook, E. Guilyardi, J. C. Hargreaves, S. P. Harrison, M. Kageyama, A. N. LeGrande, B. Konecky, S. Lovejoy, M. E. Mann, V. Masson-Delmotte, C. Risi, D. Thompson, A. Timmermann, L.-B. Tremblay, P. Yiou, Using palaeo-climate comparisons to constrain future projections in CMIP5. Clim. Past 10, 221–250 (2014).

[88] J. Zhu, C. J. Poulsen, J. E. Tierney, Simulation of Eocene extreme warmth and high climate sensitivity through cloud feedbacks. Sci. Adv. 5, eaax1874 (2019). 31555736 10.1126/sciadv.aax1874 PMC6750925

[89] W. R. Peltier, D. F. Argus, R. Drummond, Space geodesy constrains ice age terminal deglaciation: The global ICE-6G-C (VM5a) model. J. Geophys. Res. Solid Earth 120, 450–487 (2014).

[90] D. F. Argus, W. R. Peltier, R. Drummond, A. W. Moore, The Antarctica component of postglacial rebound model ICE-6G_C (VM5a) based on GPS positioning, exposure age dating of ice thicknesses, and relative sea level histories. Geophys. J. Int. 198, 537–563 (2014).

[91] P. N. DiNezio, J. E. Tierney, The effect of sea level on glacial Indo-Pacific climate. Nat. Geosci. 6, 485–491 (2013).

[92] J. W. Hurrell, J. J. Hack, D. Shea, J. M. Caron, J. Rosinski, A new sea surface temperature and sea ice boundary dataset for the community atmosphere model. J. Clim. 21, 5145–5153 (2008).

[93] P. R. Gent, G. Danabasoglu, L. J. Donner, M. M. Holland, E. C. Hunke, S. R. Jayne, D. M. Lawrence, R. B. Neale, P. J. Rasch, M. Vertenstein, P. H. Worley, Z.-L. Yang, M. Zhang, The Community Climate System Model version 4. J. Clim. 24, 4973–4991 (2011).

[94] A. Voldoire, D. Saint-Martin, S. Sénési, B. Decharme, A. Alias, M. Chevallier, J. Colin, J.-F. Guérémy, M. Michou, M.-P. Moine, P. Nabat, R. Roehrig, D. Salas y Mélia, R. Séférian, S. Valcke, I. Beau, S. Belamari, S. Berthet, C. Cassou, J. Cattiaux, J. Deshayes, H. Douville, C. Ethé, L. Franchistéguy, O. Geoffroy, C. Lévy, G. Madec, Y. Meurdesoif, R. Msadek, A. Ribes, E. Sanchez-Gomez, L. Terray, R. Waldman, Evaluation of CMIP6 DECK experiments with CNRM-CM6-1. J. Adv. Model. Earth Syst. 11, 2177–2213 (2019).

[95] P. M. Cox, R. A. Betts, C. D. Jones, S. A. Spall, I. J. Totterdell, Acceleration of global warming due to carbon-cycle feedbacks in a coupled climate model. Nature 408, 184–187 (2000). 11089968 10.1038/35041539

[96] T. Mauritsen, J. Bader, T. Becker, J. Behrens, M. Bittner, R. Brokopf, V. Brovkin, M. Claussen, T. Crueger, M. Esch, I. Fast, S. Fiedler, D. Fläschner, V. Gayler, M. Giorgetta, D. S. Goll, H. Haak, S. Hagemann, C. Hedemann, C. Hohenegger, T. Ilyina, T. Jahns, D. Jimenéz-de-la-Cuesta, J. Jungclaus, T. Kleinen, S. Kloster, D. Kracher, S. Kinne, D. Kleberg, G. Lasslop, L. Kornblueh, J. Marotzke, D. Matei, K. Meraner, U. Mikolajewicz, K. Modali, B. Möbis, W. A. Müller, J. E. M. S. Nabel, C. C. W. Nam, D. Notz, S.-S. Nyawira, H. Paulsen, K. Peters, R. Pincus, H. Pohlmann, J. Pongratz, M. Popp, T. J. Raddatz, S. Rast, R. Redler, C. H. Reick, T. Rohrschneider, V. Schemann, H. Schmidt, R. Schnur, U. Schulzweida, K. D. Six, L. Stein, I. Stemmler, B. Stevens, J.-S. von Storch, F. Tian, A. Voigt, P. Vrese, K.-H. Wieners, S. Wilkenskjeld, A. Winkler, E. Roeckner, Developments in the MPI-M Earth System Model version 1.2 (MPI-ESM1.2) and its response to increasing CO_2_. J. Adv. Model. Earth Syst. 11, 998–1038 (2019). 32742553 10.1029/2018MS001400 PMC7386935

[97] D. Paynter, T. L. Frölicher, L. W. Horowitz, L. G. Silvers, Equilibrium climate sensitivity obtained from multimillennial runs of two GFDL climate models. J. Geophys. Res. Atmos. 123, 1921–1941 (2018).

[98] K-1 Model Developers, K-1 Coupled GCM (MIROC) Description (2004).

[99] A. Yamamoto, A. Abe-Ouchi, M. Shigemitsu, A. Oka, K. Takahashi, R. Ohgaito, Y. Yamanaka, Global deep ocean oxygenation by enhanced ventilation in the Southern Ocean under long-term global warming. Global Biogeochem. Cycles 29, 1801–1815 (2015).

[100] A. G. Pendergrass, A. Conley, F. M. Vitt, Surface and top-of-atmosphere radiative feedback kernels for CESM-CAM5. Earth Syst. Sci. Data 10, 317–324 (2018).

[101] M. D. Zelinka, T. A. Myers, D. T. McCoy, S. Po-Chedley, P. M. Caldwell, P. Ceppi, S. A. Klein, K. E. Taylor, Causes of higher climate sensitivity in CMIP6 models. Geophys. Res. Lett. 47, e2019GL085782 (2020).

[102] B. J. Soden, I. M. Held, An assessment of climate feedbacks in coupled ocean–atmosphere models. J. Clim. 19, 3354–3360 (2006).

