# Supplementary material for: Last Glacial Maximum pattern effects reduce climate sensitivity estimates
Source: Sci Adv. 2024 Apr 17;10(16):eadk9461. doi: 10.1126/sciadv.adk9461 (PMC11023557; doi:10.1126/sciadv.adk9461)
Supplement: Supplementary file 1 — Supplementary Text Figs. S1 to S22 Tables S1 to S4 References [file sciadv.adk9461_sm.v2.pdf]

CORRECTED 26 APRIL 2024; SEE FULL TEXT

Science Advances
AAAS

Supplementary Materials for

Last Glacial Maximum pattern effects reduce climate sensitivity estimates

Vincent T. Cooper *et al.*

Corresponding author: Vincent T. Cooper, vcooper@uw.edu

Sci. Adv. **10**, eadk9461 (2024)
DOI: 10.1126/sciadv.adk9461

This PDF file includes:

Supplementary Text
Figs. S1 to S22
Tables S1 to S4
References

Supplementary Text

Text S1. Forcing Efficacy and Pattern Effects.

In this section, we briefly consider the relationship between "efficacy" and pattern effects, which has been investigated in a recent study (*59*). The efficacy framework (*55*) translates one unit of forcing by a non-CO_2 agent, e.g., ice sheets, into the equivalent amount of CO_2 forcing which would cause the same global-mean ΔT. While past research on forcing efficacy has considered that different forcings have different temperature impacts (*55*), analyses using the efficacy framework for the LGM have produced disparate results (*22*, *23*, *42*, *43*, *48*, *56*), possibly due to simplified physics of intermediate-complexity models (*42*, *43*). Because of these results, WCRP20 inflates uncertainty on LGM forcings.

Efficacy, ε, can be equivalently framed as a ratio of radiative feedbacks (*58*, *59*), e.g., $\varepsilon_{IceSheet}=\lambda_{2x}/\lambda_{IceSheet}$. The negative LGM pattern effect ($\Delta\lambda=\lambda_{2x}-\lambda_{LGM}$, $\Delta\lambda<0$), which we find in AGCM simulations using data-assimilation reconstructions for the LGM, is consistent with an LGM efficacy greater than 1. The efficacy of ice sheets is greater than 1 in the following model-only studies with mixed-layer oceans coupled to atmospheric general circulation models: CESM1-CAM5 (*23*), CESM2 (*48*), and CESM2-PaleoCalibr (*49*) (SI Appendix, Text S2). Some intermediate-complexity models (*42*, *43*), however, have reported ice-sheet efficacy less than 1.

The pattern effect, combined with temperature dependence, can equivalently explain forcing efficacy (*59*). We use the pattern-effect framework rather than efficacy because it allows for quantification of feedback changes in AGCMs using observational constraints on SST patterns from data assimilation and has strong theoretical underpinnings (*12*, *18*, *59*). The pattern-effect framework is oriented around the climate feedback, λ, which is the key uncertain parameter for climate sensitivity. We follow methods in WCRP20 (*1*) to account for Δλ for the LGM in estimates of modern-day climate sensitivity. We refer readers to Zhou et al. (2023) (*59*) for further explanation of the connection between efficacy and pattern-effect frameworks.

Text S2. LGM Pattern Effects in Coupled Models.

Simulations with mixed-layer ocean models coupled to AGCMs (known as slab ocean models (*47*), "SOM" hereafter) in CESM1-CAM5 (*23*), CESM2.1-CAM6 (*48*), and CESM2-PaleoCalibr (*49*) illustrate pattern effects in coupled models. Note that feedbacks from ocean dynamics are excluded in the SOM, and models' SST/SIC patterns are not constrained by proxy data, hence we use the SOM only to support interpretation of the LGM pattern effect. Feedbacks in SOM simulations are calculated as $\lambda=\Delta ERF/\Delta T$, where the effective radiative forcing (ERF) is determined from introducing forcings in separate simulations in the corresponding AGCMs (keeping SST/SIC fixed at pre-industrial values), and ΔT is the equilibrium change in global-mean near-surface air temperature in the SOM (also known as reference-height temperature, or "TREFHT" in CESM name conventions). The ERF is affected by changes in land-surface temperatures, which are not held constant in AGCM simulations due to practical limitations, and an adjustment (*23*, *55*) to the ERF can be made to account for land changes—see Zhu & Poulsen (2021) (*23*) for methods.

This adjustment, which is based on a climate sensitivity parameter (*23*) can also be applied to estimate an "adjusted ERF" for LGM ice sheets, although it is difficult to assess the validity of the adjustment for ice-sheet forcing, which affects not only land temperatures but also topography. Radiative kernels based on modern climate would typically be used to validate the

ERF adjustment (*23*), but they cannot be applied with LGM topography. SI Appendix, Figure S11, shows feedbacks from coupled models using both ERF and adjusted ERF. Note that these values do not affect our quantification of $\Delta\lambda$ for ECS calculations, which comes from AGCM simulations.

Text S3. Preparation of SST/SIC Boundary Conditions.

SST and SIC boundary conditions (BCs) for the LGM, Late Holocene baseline, and $2xCO_2$ are prepared to enable consistent calculation of the net feedback (λ) that is applicable to a modern-day doubling of CO_2. When changing the surface BCs in AGCM simulations to compute λ, $\Delta F=0$ in Eq. 1 only if there are no changes in land-sea distribution or ice-sheets. For the LGM and Late Holocene datasets, we adjust for differences in land-sea distribution, determined from refs. (*89*, *90*), compared to present day using kriging and extrapolation near coastlines in polar regions. While sea-level changes must be neutralized to preserve $\Delta F=0$ in the AGCM simulations, infilling SST over the Sunda Shelf represents a notable uncertainty (*28*, *91*). The alternative option, holding all forcings constant at LGM rather than modern values, would require changing modern topography to include LGM ice sheets and inherit sea level of the LGM. Those changes could introduce more uncertainty in estimates of λ that are relevant to future warming. Here we only consider the framework with constant modern-day forcings.

For SST, kriging is performed across overlapping subset regions of radius≈3000 km spaced around the globe. Results for overlapping subset regions are merged using inverse-distance weighting from the center of each subset region. Kriging results are retained only where no pre-existing SST value exists in a dataset. Over polar regions and inland waters, inverse-distance extrapolation populates the SST field.

For SIC, all values are first required to be no less than the ice-sheet fraction at that location, i.e., modern seas that were covered by ice sheets at the LGM, such as the Hudson Bay, are assigned a minimum SIC that equals the LGM ice fraction at 21,000 years ago (*89*, *90*). For modern seas which were land but not ice sheet at the LGM, SIC is populated based on the SST. This step uses the SIC formula from the CAM boundary condition protocol (*92*), where SIC=100% if SST<–1.8°C, SIC=0% if SST>4.97°C, and otherwise the infilled $SIC=0.729-((SST+1.8)/9.328)^{1/3}$. Gaussian smoothing is applied to the result, reducing any sharp boundaries caused by the infilling. The SIC formula above is also applied to maintain internally consistent values of SST and SIC (*92*) in the Late Holocene baseline. See SI Appendix, Text S4, for uncertainty tests regarding sea ice.

The Annan dataset includes only annual SST and no reconstruction of SIC. Because SIC is required in all AGCMs, we assign the SIC from Amrhein to the Annan data. In a CAM4 test using the LGMR SIC with Annan SSTs (instead of the Amrhein SIC), $\Delta\lambda$ is marginally more negative (λ_{LGM} changes by < 0.1 $Wm^{-2}K^{-1}$). This result suggests that uncertainty from assigning a SIC reconstruction to Annan SSTs is small compared to uncertainty in the SST reconstruction. We assign the Amrhein SIC for the Annan SST in our main results because this choice is more conservative in that it reduces the magnitude of the mean LGM pattern effect. For consistency, the Annan SST is assigned the annual cycle from the Amrhein data for SST/SIC.

For the $2xCO_2$ BC, we use output from LongRunMIP (*39*) simulations of abrupt and transient-1% yr^{-1} doubling of CO_2. We use the mean of 200 years of output from the following six models in to create a multi-model mean SST/SIC BC: CESM1.0.4 (*93*) years 2300–2500, CNRM-CM6-1 (*94*) years 550–750, HadCM3L (*95*) years 500–700, MPI-ESM-1.2 (*96*) years 800–1000, GFDL-ESM2M (*97*) years 4300–4500, and MIROC3.2 (*98*, *99*) years 1803–2003.

HadCM3L results use years 500-700 due to an output error in the pre-industrial control run after year 700. All LongRunMIP results are regridded to a standard 1.9° x 2.5° lat-lon grid. For SIC, monthly output is available, and we compute a 200-yr climatology for each model and then a multi-model-mean climatology. For SST, annual output is available for each model and monthly output from MIROC3.2. We compute the 200-yr mean SST anomaly for each model and then apply the annual cycle from MIROC3.2 to the multi-model mean. We also show results in SI Appendix, Fig. S3–S4, which do not use the LongRunMIP-2xCO_2 BC and instead use 150-year regressions (*73*) of abrupt-4xCO_2 from parent coupled models corresponding to each AGCM used in this study, thereby sampling uncertainty in warming patterns because the 150-year regressions are produced from different models' warming patterns.

BCs are regridded to the 1.9° x 2.5° (latitude x longitude) grid used for CAM4, CAM5, and CAM6. HadGEM3-GC31-LL regrids to N96 (resolution of approximately 135 km) (*70*), and GFDL-AM4 regrids to a C96 cubed sphere (resolution of approximately 100 km) (*71*).

For the "pattern-only" simulations with SST anomalies normalized to −0.5 K, we make the following changes to the LGM and 2xCO_2 BCs. For the LGM, we use the LGMR SST. For 2xCO_2, we use the LongRunMIP SST. We compute the global-mean ΔSST for both datasets as $\overline{\Delta SST}$, and we multiply all local SST anomalies by the scale factor $-0.5/\overline{\Delta SST}$. This scaling causes the resulting global-mean ΔSST to become −0.5 K, but the spatial pattern of the SST anomalies is unchanged. We use −0.5 K for both the LGM and 2xCO_2 so that there is no cooling-warming asymmetry, and ΔT is small enough that temperature dependence of λ is negligible (i.e., $\Delta\lambda_T \approx 0$, and $\Delta\lambda \approx \Delta\lambda_{PatternOnly}$). ΔT is still large enough that we can compute $\lambda = \Delta N/\Delta T$ without requiring an excessively long simulation to overcome noise in the denominator. We use the baseline SIC (Late Holocene) in all of the pattern-only simulations so there are no changes in sea ice, so this set of simulations also serves to check whether Δλ is attributable to SIC rather than SST changes.

To examine whether the pattern-only results are sensitive to the scaling method of separating pattern effects, we tested an alternative subtraction method in CAM4 (using the LGMR pattern for the LGM and the LongRunMIP pattern for 2xCO_2). We ran alternative pattern-only simulations with global-mean SST anomalies set to zero by subtracting the global mean at all locations. These experiments produced consistent results for $\Delta\lambda_{PatternOnly}$ compared to scaling.

An additional simulation was run in HadGEM3-GC3.1-LL with SIC held constant at the Late Holocene baseline while the SST field is varied with the full value of anomalies, using the LongRunMIP-2xCO_2 and LGMR patterns of SST. Results from this simulation are shared in SI Appendix, Text S4.

This concludes the preparation steps for the main simulations (BCs from four data-assimilation reconstructions for the LGM, one Late Holocene, and one 2xCO_2) and the "pattern-only" simulations (two additional BCs: LGMR and LongRunMIP-2xCO_2 scaled to −0.5 K). The final adjustment to each BC follows the standard boundary-condition protocol for CAM, known as "bcgen." This process ensures that SIC and SST are plausibly bounded (e.g., SIC between 0 and 1), and it transfers the monthly climatology to mid-month values which can be linearly interpolated in an AGCM.

Text S4. Uncertainty of Δλ.

To include the LGM pattern effect in the Bayesian framework of WCRP20, we must assign a statistical distribution to Δλ for the LGM (following WCRP20's method for Δλ in the historical

record). In this section we provide additional detail on combining uncertainty from AGCM physics and LGM reconstructions with bootstrapping.

To evaluate the sensitivity of our uncertainty quantification to the size of our sample of AGCMs and reconstructions, we calculate a bootstrap confidence interval (CI) on our estimate, $\widehat{\sigma}$, of the standard deviation of $\Delta\lambda$ as follows. First, we construct a sample where each AGCM is equally weighted and the spread from various LGM reconstructions is included in the sample (as described below). We then use bootstrapping of this sample to provide confidence bounds on our estimate ($\widehat{\sigma}$) of the population standard deviation from the sample standard deviation.

To create the equally weighted sample, we assume that the spread around the LGMR feedback (of the feedbacks from Amrhein, Annan, and lgmDA) would be the same in GFDL-AM4, HadGEM3-GC3.1-LL, and CAM6 as they are in CAM4 or CAM5. We include the simulations using the extreme quartiles from Annan and LGMR in the sample. This assumption yields a sample of 40 values of $\Delta\lambda$ based on (4 LGM patterns + 2 extreme-quartile LGMR patterns + 2 extreme-quartile Annan patterns) x (5 AGCMs). We proceed with bootstrapping by sampling with replacement from the 40 values of $\Delta\lambda$. We generate 10^5 samples of size n=19, choosing this sample size for the bootstrap because there are 19 direct estimates of $\Delta\lambda$ from simulations in the AGCMs. This process yields 10^5 bootstrapped values of $\widehat{\sigma}$ from which we derive the 95% CI: (0.15, 0.31) $Wm^{-2}K^{-1}$. Note that the upper bound of 0.31 $Wm^{-2}K^{-1}$ is much less than two times the population standard deviation of 0.23 $Wm^{-2}K^{-1}$ that we assign to $\Delta\lambda$, indicating that doubling the assumed standard deviation for $\Delta\lambda$ is a more conservative uncertainty test (SI Appendix, Fig. S4) than using the bootstrapped 95% bound.

To determine the distribution of $\Delta\lambda$ in SI Appendix, Figure S4, we repeat the bootstrap estimate using $\lambda_{4x(150yr)}/1.06$ instead of λ_{2x}, where 1.06 represents WCRP20's central estimate (*1*) for the timescale adjustment between the 150-year feedback and the equilibrium feedback; this yields $\overline{\Delta\lambda}$=−0.27 $Wm^{-2}K^{-1}$ and mean sample standard deviation of 0.20 $Wm^{-2}K^{-1}$.

Our method of combining uncertainty gives equal weight to the most-extreme quartiles and to the central estimates, but this overestimate of uncertainty is warranted given that paleoclimate data assimilation may underestimate the true uncertainty (*35*). The uncertainty estimate also gives more weight to the most recent reconstructions, LGMR (*32*) and Annan (*33*), by including three simulations (mean, 1st quartile, and 4th quartile) from these datasets. The weighting influences the bootstrap estimate and the distribution assigned to $\Delta\lambda$ in our calculations of ECS.

Over the range of temperatures between the LGM and $2xCO_2$, all five AGCMs appear to have weaker temperature dependence of feedbacks than WCRP20 assumes, i.e., $\Delta\lambda_T$ appears smaller than in WCRP20. $\Delta\lambda_T$ could be underestimated in all models, so we include an uncertainty test where we use the pattern-only simulations in CAM4, CAM5, and CAM6 to estimate the mean $\Delta\lambda_{PatternOnly}$ contribution to the total $\Delta\lambda$, and we retain WCRP20's estimate of $\Delta\lambda_T$. In this uncertainty test, $\Delta\lambda$ in Eq. 6 is calculated as the sum of $\Delta\lambda_T$ and $\Delta\lambda_{PatternOnly}$: $\Delta\lambda_T = -\alpha\Delta T/2$ with $\alpha \sim N(0.1, 0.1)$ $Wm^{-2}K^{-2}$ as in WCRP20, while $\Delta\lambda_{PatternOnly} \sim N(-0.51, 0.23)$ $Wm^{-2}K^{-1}$ with μ based on CAM4, CAM5, and CAM6 results (SI Appendix, Table S3). The results of this uncertainty test are included in SI Appendix, Figure S9, indicating that accounting for pattern effects causes the dominant change to LGM evidence for ECS, while the revision to WCRP20's temperature dependence contributes a smaller portion of the update.

Sea-ice reconstructions, which are not well constrained, contribute to uncertainty in the LGM pattern effect. However, the uncertainty due to sea ice appears small compared to the uncertainty across AGCM physics and in the SST pattern. In an additional set of simulations with HadGEM3-GC3.1-LL, the SST anomalies are applied in full at the LGMR, Late Holocene,

and LongRunMIP-2xCO_2 values while the SIC is held constant at the Late Holocene values. These simulations make λ_{2x} and λ_{LGM} more negative by eliminating the positive ice-albedo feedback, but the difference in the feedbacks, $\Delta\lambda$, is largely unaffected. Constant SIC produces $\Delta\lambda = -0.28$ $Wm^{-2}K^{-1}$, compared to -0.27 $Wm^{-2}K^{-1}$ in the main simulations for HadGEM3-GC3.1-LL. SIC is also held constant in the pattern-only simulations, which produce $\Delta\lambda<0$. While our results appear robust despite uncertainty in SIC, substantially different LGM reconstructions or SIC responses to modern-day 2xCO_2 could change the resulting $\Delta\lambda$. Future work should continue investigating the role of sea ice in paleoclimate pattern effects.

Text S5. Zonal-mean Feedbacks.

SI Appendix, Figures S12–S22 show zonal means (indicated by brackets as [λ]) of the global-mean feedbacks that appear in SI Appendix, Figure S6. The net feedback, clear-sky shortwave (SW), clear-sky longwave (LW), and cloud radiative effect are calculated directly from model output. The remaining feedbacks are from radiative kernel decomposition (Materials and Methods) using CAM5 kernels (*77*, *100*). GFDL-AM4's 2xCO_2 simulation has error in the kernel-derived clear-sky feedback equal to 15.6% of the actual feedback, exceeding the 15% threshold commonly used as a test of clear-sky linearity (*15*, *76*, *101*); all other simulations have clear-sky feedback errors less than 10%. Total cloud feedback is also shown as the sum of kernel-derived SW and LW components.

Each of the zonal-mean figures consists of: (A) In CAM5, mean and range of feedbacks across four LGM reconstructions and 2xCO_2 from LongRunMIP. (B) In CAM5, mean and range of the difference in feedbacks ($\Delta\lambda = \lambda_{2x} - \lambda_{LGM}$) across four LGM reconstructions from results in panel A. (C) Feedbacks across various AGCMs, using the LGMR reconstruction of the LGM and 2xCO_2 from LongRunMIP. (D) Mean and range of $\Delta\lambda$ across various AGCMs from results in panel C. Note that HadGEM3 is not included in the kernel-derived feedbacks due to limited availability of model output.

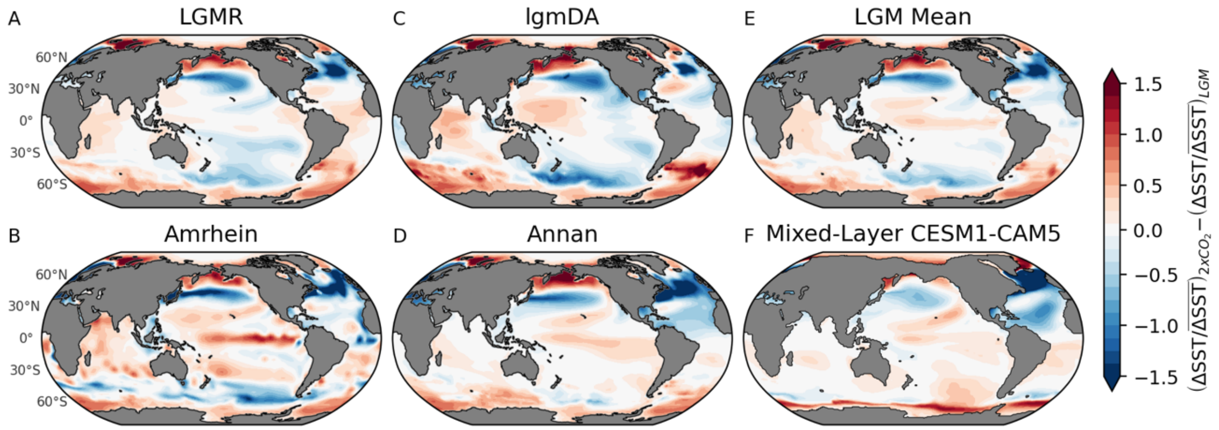

Fig. S1. Differences in LGM sea-surface temperature (SST) patterns compared to $2xCO_2$ reference pattern.

All local anomalies are normalized through division by global-mean anomaly, then differences between the $2xCO_2$ pattern and LGM pattern are taken. Red regions indicate where SST anomalies are relatively more amplified in $2xCO_2$, while blue regions indicate where SST anomalies are relatively more amplified at the LGM. (**A–E**), LGM patterns corresponding to Fig. 1A–E, and $2xCO_2$ reference pattern is Fig. 1F from LongRunMIP-$2xCO_2$. (**F**) In CESM1-CAM5 (*23*) mixed-layer ocean model without data assimilation, difference between $2xCO_2$ and LGM patterns (shown in Fig. S5C–D).

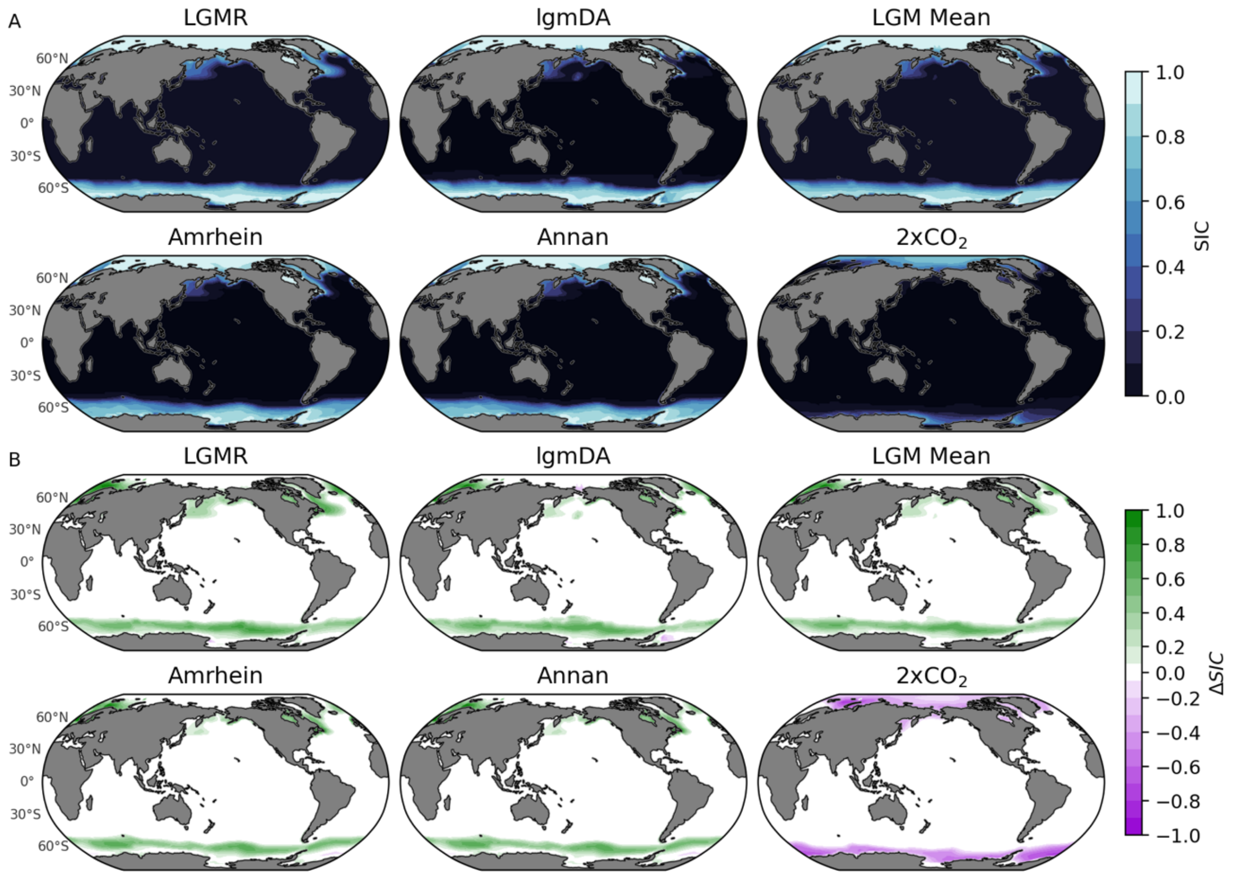

Fig. S2. Sea-ice concentration (SIC) from data-assimilation reconstructions of the Last Glacial Maximum (LGM) compared to 2xCO_2.

(**A**) SIC from LGM Reanalysis (LGMR) (*32*), Amrhein (*34*), lgmDA (*3*), Annan (*33*) (assigned SIC from Amrhein); mean of three LGM reconstructions (LGMR, Amrhein, and lgmDA); and multi-model mean from near-equilibrium simulations of 2xCO_2 in LongRunMIP (*39*), where each of six models is averaged over final 200 years of simulation. (**B**) Difference in sea-ice concentration relative to Late Holocene baseline (LGMR reconstruction). All panels show annual mean. Reconstructions are infilled to modern coastlines (Materials and Methods).

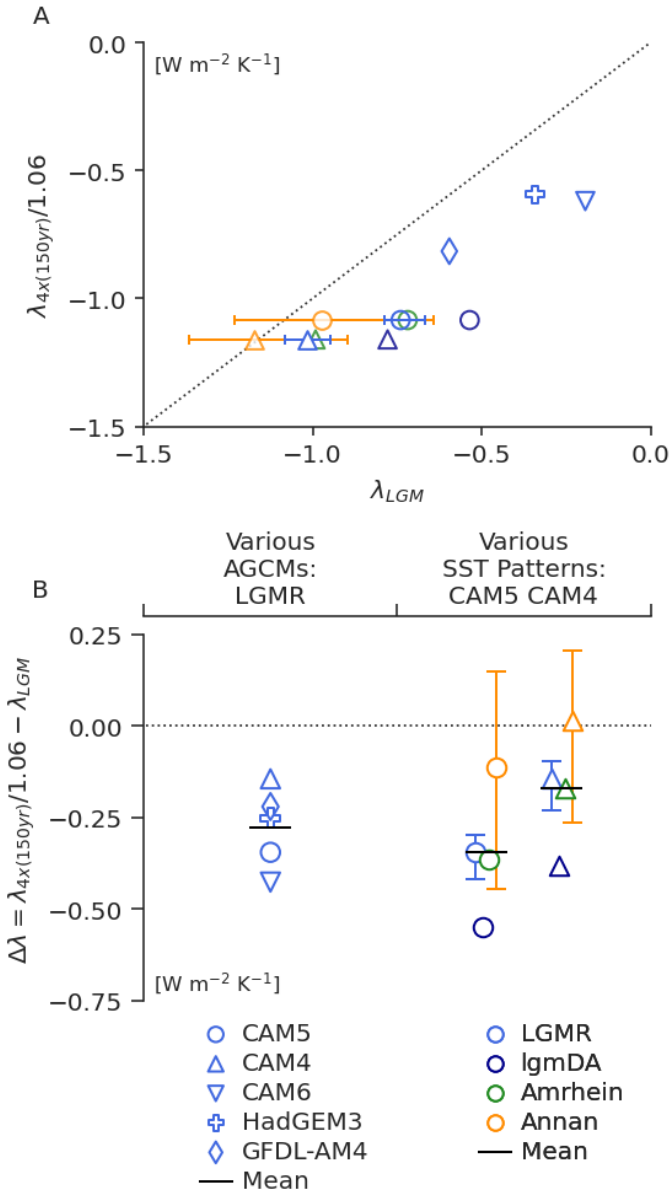

Fig. S3. LGM pattern effect (Δλ) based on LGM climate feedbacks in AGCMs and CO_2 climate feedbacks from 150-yr regression of abrupt-4xCO_2 in coupled models.

Similar to Fig. 2, except λ_{2x} is replaced by $\lambda_{4x(150yr)}/1.06$, the feedback from regression in abrupt-4xCO_2 simulations (*73*) using parent coupled models corresponding to each AGCM; a timescale adjustment of 1/1.06 is applied based on the WCRP20 central estimate (*1*) to make 150-year 4xCO_2 feedbacks comparable with λ_{LGM} equilibrium feedbacks. Different models (all using the LGMR pattern for the LGM) are indicated by symbols. Different LGM patterns (in CAM5 and CAM4) are indicated by colors. (**A**) Scatter plot of 4xCO_2 feedbacks (including adjustment factor of 1/1.06) versus LGM feedbacks, with $\lambda_{4x(150yr)}/1.06=\lambda_{LGM}$ shown as dashed line. (**B**) LGM pattern effect, $\Delta\lambda= \lambda_{4x(150yr)}/1.06-\lambda_{LGM}$, using feedbacks shown in (A), with $\Delta\lambda=0$ shown as dashed line. Note that Δλ includes SST pattern effects and contributions from temperature dependence.

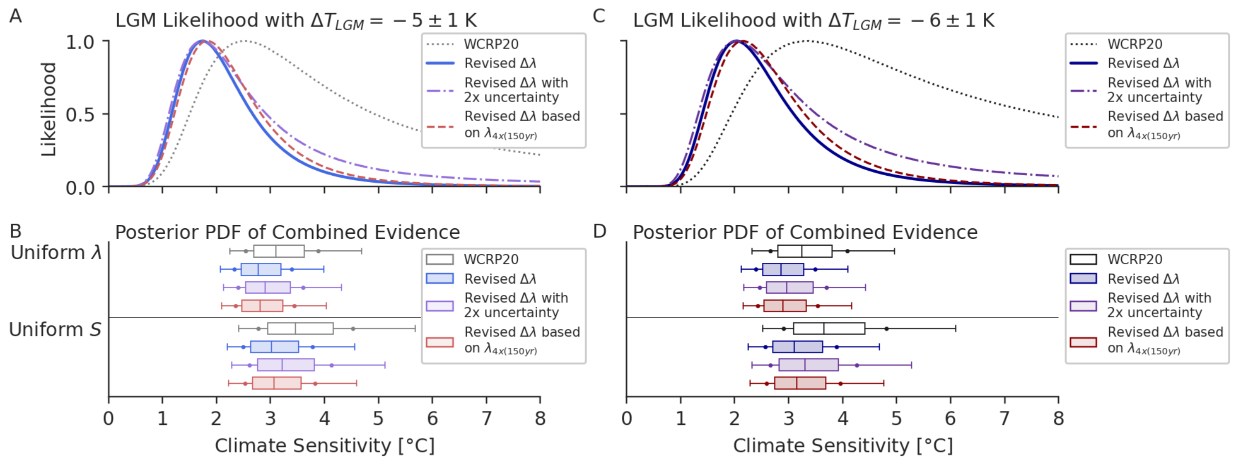

Fig. S4. Uncertainty tests for modern-day climate sensitivity including LGM pattern effects.

Following Fig. 4, showing WCRP20 original (*1*) LGM $\Delta T_{LGM} \sim N(\mu=-5, \sigma=1)$ K in left column and revised LGM $\Delta T_{LGM} \sim N(-6, 1)$ K based on IPCC AR6 (*2*) in right column, including two uncertainty tests. Results from WCRP20 (*1*) with no LGM pattern effect (gray and black) and our base assumption (light and dark blue) for revised $\Delta\lambda \sim N(-0.37, 0.23)$ $Wm^{-2}K^{-1}$ from Fig. 4 are repeated here for comparison. First uncertainty test (light and dark purple) increases the σ assumption by a factor of two: $\Delta\lambda \sim N(-0.37, 0.46)$ $Wm^{-2}K^{-1}$. Second uncertainty test (light and dark red) concerns the $2xCO_2$ pattern and feedback: a different distribution, $\Delta\lambda \sim N(-0.27, 0.20)$ $Wm^{-2}K^{-1}$, is assigned based on results shown in Ext. Data Fig. 3 using $\lambda_{4x(150yr)}/1.06$, the feedback derived from 150-year regressions (*73*) of abrupt-$4xCO_2$ using parent coupled models corresponding to each AGCM, including a timescale-adjustment factor of 1/1.06 from WCRP20's central estimate (*1*). Climate sensitivity shown is effective sensitivity (*S*) from 150-year response, as in WCRP20 (*1*). (**A**) Likelihood functions for *S* based on only the LGM line of evidence. (**B**) Posterior PDF after combining LGM with other lines of evidence in WCRP20 (*1*), assuming a uniform-λ prior (upper panel) or a uniform-*S* prior (lower panel). Outlier lines indicate 5–95th percentiles, dots indicate 66% *likely* range, and box indicates 25–75th percentiles and median.

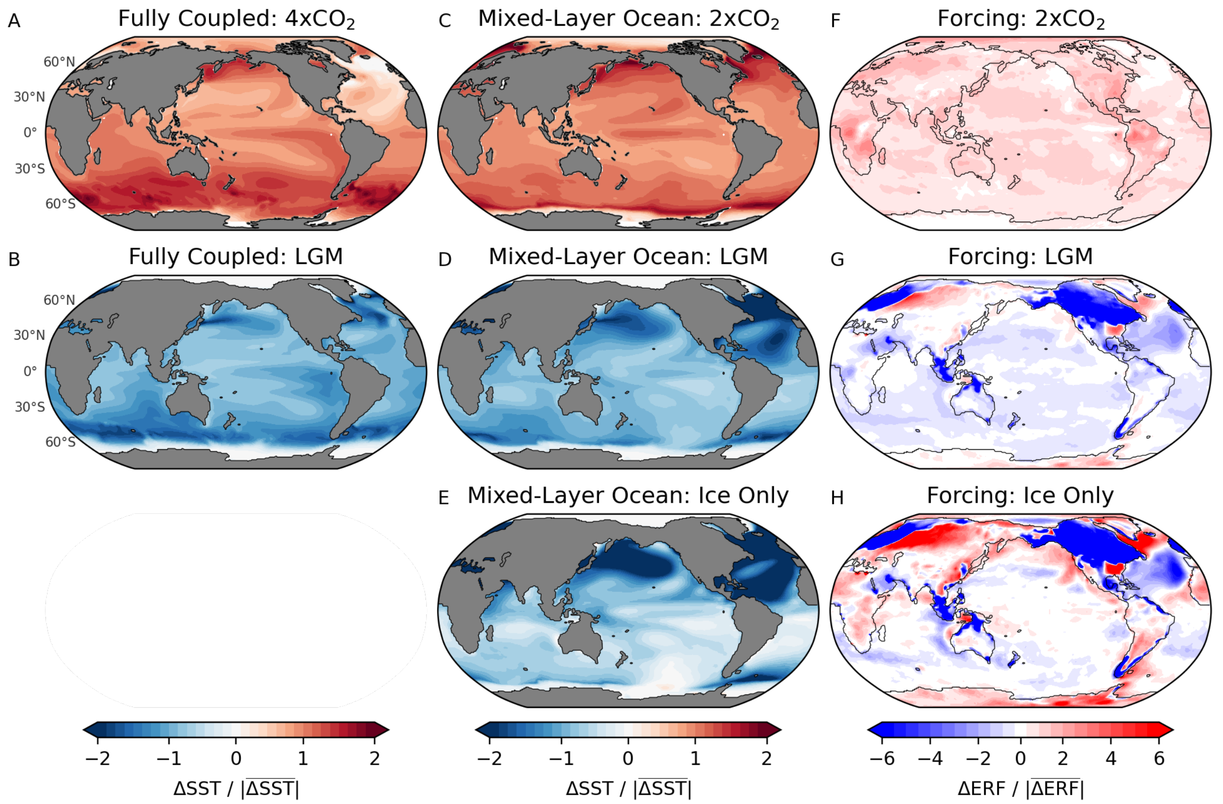

Fig. S5. Spatial patterns of sea-surface temperature (SST) response and effective radiative forcing (ERF) in CESM1-CAM5 model simulations from Zhu & Poulsen (*23*).

Spatial patterns here are shown as zonal means in Fig. 2. All local anomalies are normalized through division by absolute value of global-mean anomaly. (**A–B**) SST patterns in quasi-equilibrium from fully coupled atmosphere-ocean model with LGM ice-sheet and greenhouse-gas forcings (*23*) compared to abrupt-4xCO_2. (**C–E**) Equilibrium SST patterns from mixed-layer ocean model coupled to CAM5, including a simulation with only LGM ice-sheet forcing (*23*). (**F–H**) ERF patterns from corresponding AGCM simulations in CAM5.

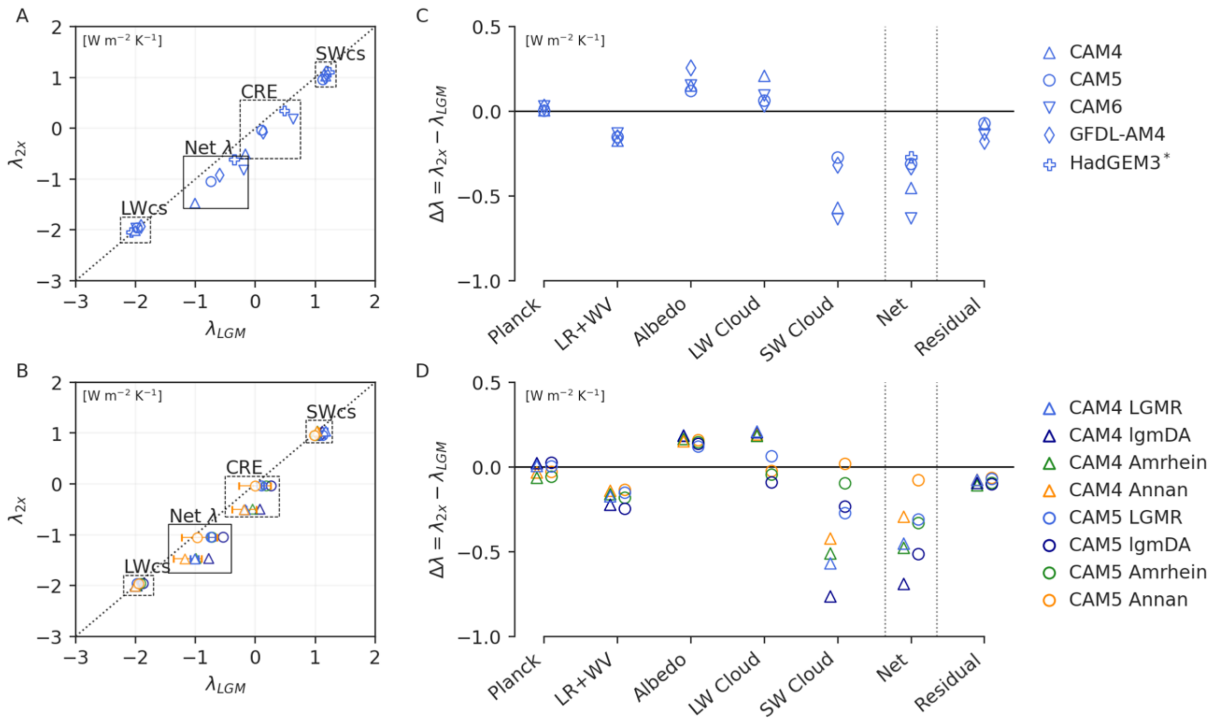

Fig. S6. Feedback decomposition of Last Glacial Maximum (LGM) and 2xCO_2 climate feedbacks in atmospheric general circulation models (AGCMs).

Left column uses direct model outputs in scatter plots of 2xCO_2 feedbacks (λ_{2x}) versus LGM feedbacks (λ_{LGM}), with λ_{2x}=λ_{LGM} denoted by dashed line. Cloud radiative effect (CRE), shortwave clear-sky (SWcs), longwave clear-sky (LWcs), and net feedbacks are shown. (**A**) Results from various AGCMs, all using the LGMR reconstruction for the LGM. (**B**) Results from various LGM reconstructions in CAM4 and CAM5, with different reconstructions indicated by colors. Right column shows decomposition of $\Delta\lambda$ using CAM5 radiative kernels (*100*), with residual equal to the net feedback in models minus the sum of kernel-derived feedbacks. (**C**) Results from various AGCMs (note that only net λ is available for HadGEM3). (**D**) Results from various LGM reconstructions in CAM4 and CAM5. Lapse rate and water vapor feedbacks are combined (LR+WV) given their anti-correlation across models (*102*). Note that $\Delta\lambda$ includes SST pattern effects and contributions from temperature dependence.

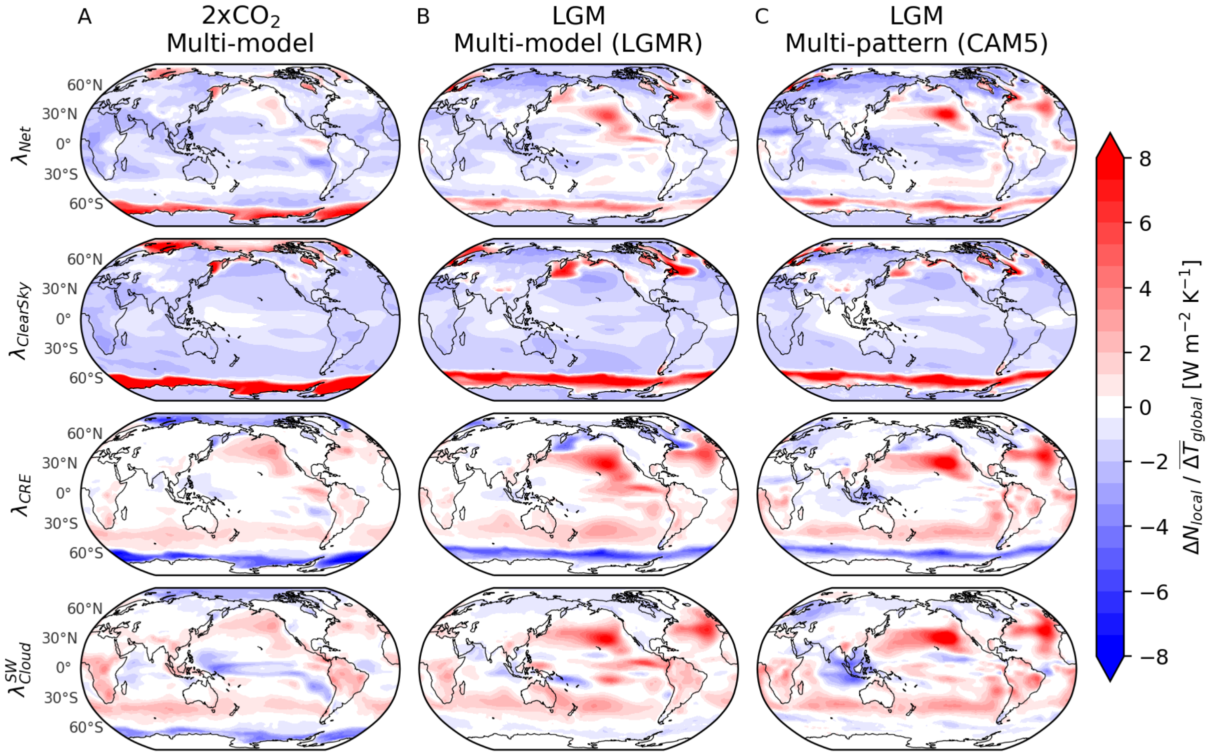

Fig. S7. Spatial decomposition of Last Glacial Maximum (LGM) and 2xCO_2 local climate feedbacks in atmospheric general circulation models (AGCMs).

Local feedbacks represent local change in top-of-atmosphere radiation (ΔN_{local}) divided by global-mean change in near-surface air temperature (ΔT_{global}); global integrals of the local feedbacks equal the global-mean feedbacks. Top row shows net feedback (λ_{Net}) from total all-sky changes in ΔN, second row shows $\lambda_{ClearSky}$ from changes in ΔN attributable to clear-sky radiation, third row shows cloud radiative effects (λ_{CRE}); rows 1–3 use direct model output. Fourth row shows radiative-kernel estimates of shortwave cloud feedbacks (λ_{Cloud}^{SW}). (**A**) 2xCO_2 multi-model mean based on five AGCM simulations using LongRunMIP (*39*) pattern. (**B**) LGM multi-model mean based on five AGCM simulations using LGMR (*32*) pattern. (**C**) LGM multi-pattern mean in CAM5 using four LGM reconstructions. Note that radiative-kernel results for λ_{Cloud}^{SW} exclude HadGEM3 due to output limitations.

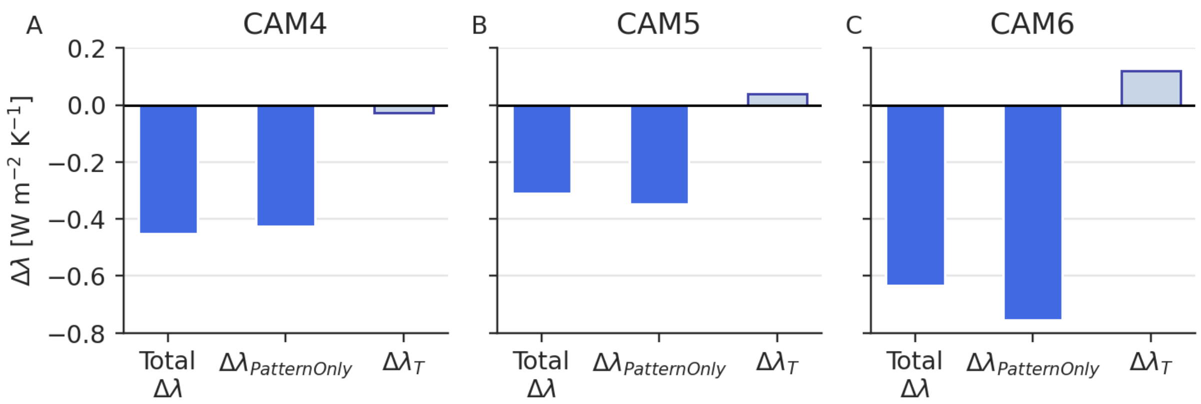

Fig. S8. Separating pattern and temperature dependence of feedback changes as total $\Delta\lambda \approx \Delta\lambda_{PatternOnly} + \Delta\lambda_T$.

First column shows total $\Delta\lambda = \lambda_{2x} - \lambda_{LGM}$ from Figure 2, calculated in main simulations with full SST anomalies and SIC for 2xCO_2 and LGM (using LGMR reconstruction). Second column shows pattern-only simulations with global-mean ΔSST scaled to −0.5 K, where $\Delta\lambda_{PatternOnly} \approx \lambda_{2x}^{-0.5K} - \lambda_{LGM}^{-0.5K}$. Third column shows temperature dependence, $\Delta\lambda_T$, approximated as the residual difference between the main and pattern-only simulations, $\Delta\lambda_T \approx \Delta\lambda - \Delta\lambda_{PatternOnly}$. Results in (**A**) CAM4, (**B**) CAM5, and (**C**) CAM6.

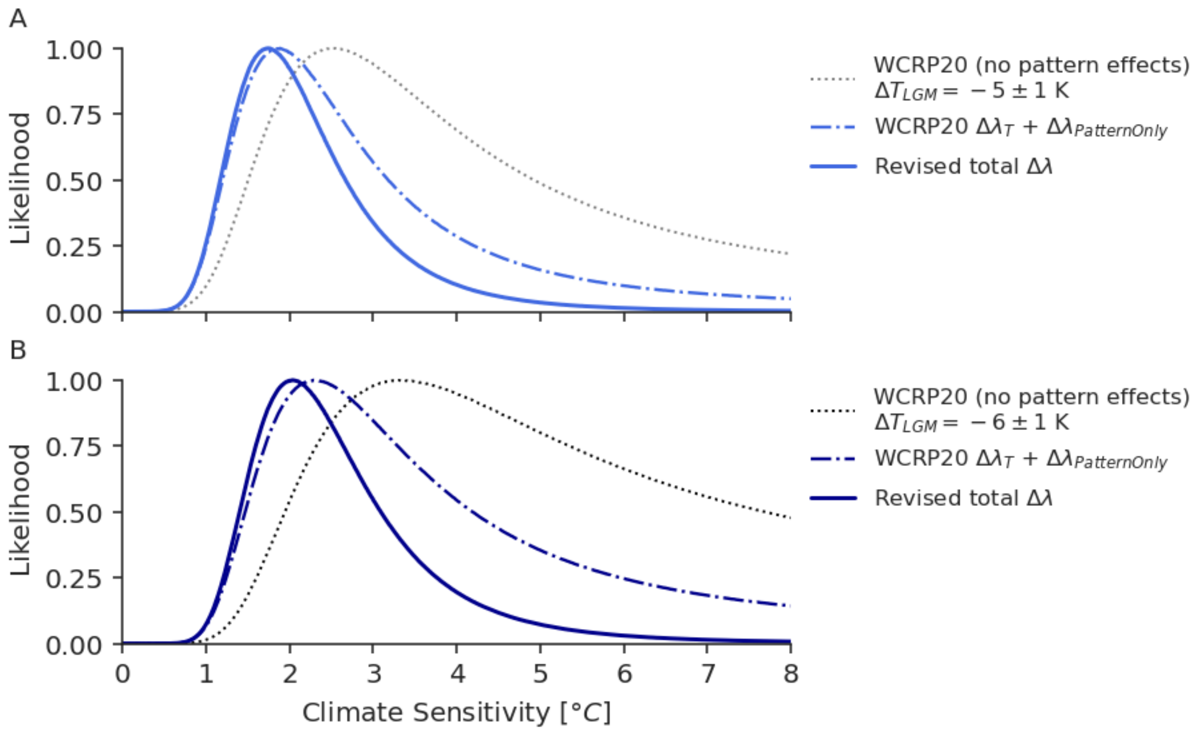

Fig. S9. Likelihoods for LGM line of evidence with separate updates for SST pattern effects and temperature dependence of feedbacks.

(Dotted) WCRP20 LGM likelihood (*1*), which includes an estimate of $\Delta\lambda_T$ for the LGM but no adjustment for pattern effects. (Dash-dot) Revised likelihood using WCRP20 estimate of $\Delta\lambda_T$ but including feedback changes from SST patterns based on pattern-only simulations in this study, assuming $\Delta\lambda_{PatternOnly} \sim N(\mu=-0.51, \sigma=0.23)$ $Wm^{-2}K^{-1}$. (Solid) Revised likelihood using total revised $\Delta\lambda$ from this study, as shown in Fig. 4, which includes both pattern effects and temperature dependence, assuming $\Delta\lambda \sim N(-0.37, 0.23)$ $Wm^{-2}K^{-1}$. (**A**) All likelihoods assume $\Delta T_{LGM} \sim N(-5, 1)$ K as in original WCRP20 results (*1*). (**B**) All likelihoods assume $\Delta T_{LGM} \sim N(-6, 1)$ K, using the updated central estimate from IPCC AR6 (*2*).

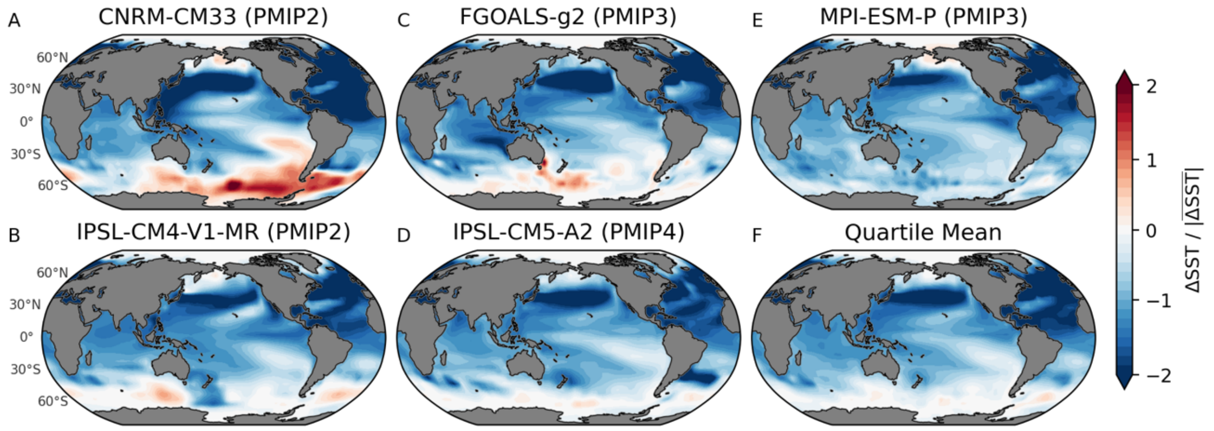

Fig. S10. Patterns of SST anomalies from Annan (*33*) ensemble members in quartile with strongest negative climate feedback (λ).

19 ensemble members are ranked by estimated λ, which is produced from CAM5 Green's functions (*18*), and 5 members shown comprise the quartile with most-negative estimated λ. (**A–E**) Data-assimilation posterior SST using model priors specified in subtitles. (**F**) Pattern of the quartile-mean SST. To show SST patterns, local SST anomalies are normalized into patterns through division by absolute value of global-mean SST anomaly (consistent with feedbacks being radiative responses divided by global-mean temperature anomalies). All panels show annual means. LGM reconstructions are infilled to modern coastlines (Materials and Methods).

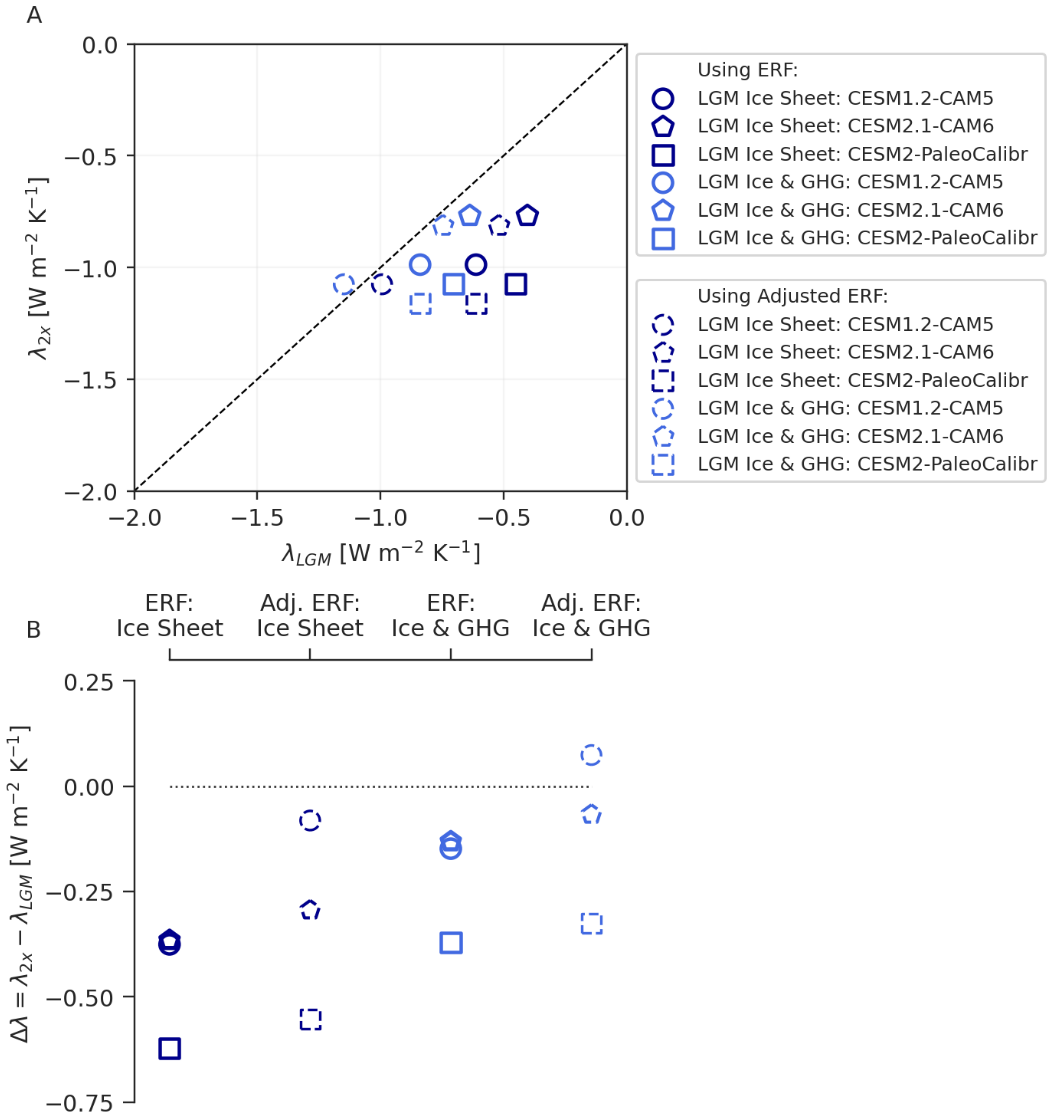

Fig. S11. Feedbacks and Δλ using either effective radiative forcing (ERF) or adjusted ERF from previously published simulations in mixed-layer ocean models.

(**A**) Scatter plot of λ_{2x} vs. λ_{LGM} in mixed-layer ocean models; λ_{LGM} is shown for simulations using only the LGM ice-sheet forcing (dark blue), which includes LGM sea-level changes, and for simulations using LGM ice-sheet forcing and greenhouse-gas (GHG) forcings (royal blue). Dashed markers indicate corresponding results using "adjusted ERF" to calculate feedbacks. (**B**) $\Delta\lambda$ based on feedbacks shown in panel A. Note that in LGM simulations using CESM2.1-CAM6 (*48*) and CESM2-PaleoCalibr (*49*), the LGM ice-sheet forcing and GHG forcing are applied in separate simulations, and their sums are shown as LGM Ice & GHG. This linearity assumption was validated in CESM1-CAM5 (*23*).

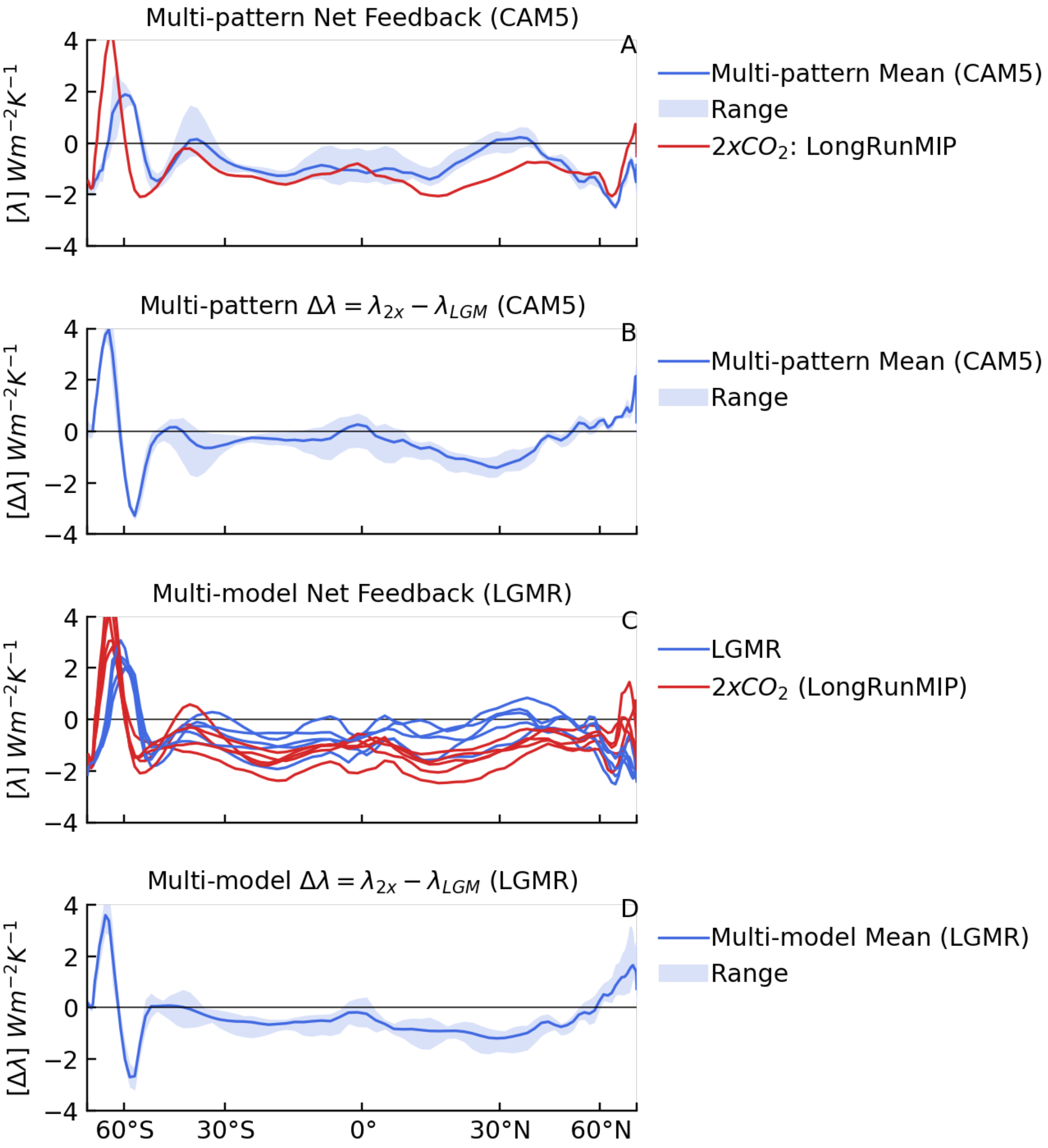

Fig. S12. Zonal-mean net feedback and Δλ.

(**A**) In CAM5, mean and range of feedbacks across four LGM reconstructions and $2xCO_2$ from LongRunMIP. (**B**) In CAM5, mean and range of the difference in feedbacks ($\Delta\lambda = \lambda_{2x} - \lambda_{LGM}$) across four LGM reconstructions from results in (A). (**C**) Feedbacks across various AGCMs, using the LGMR reconstruction of the LGM and $2xCO_2$ from LongRunMIP. (**D**) Mean and range of Δλ across various AGCMs from results in (C). Note that HadGEM3 is not included in the kernel-derived feedbacks due to limited model output.

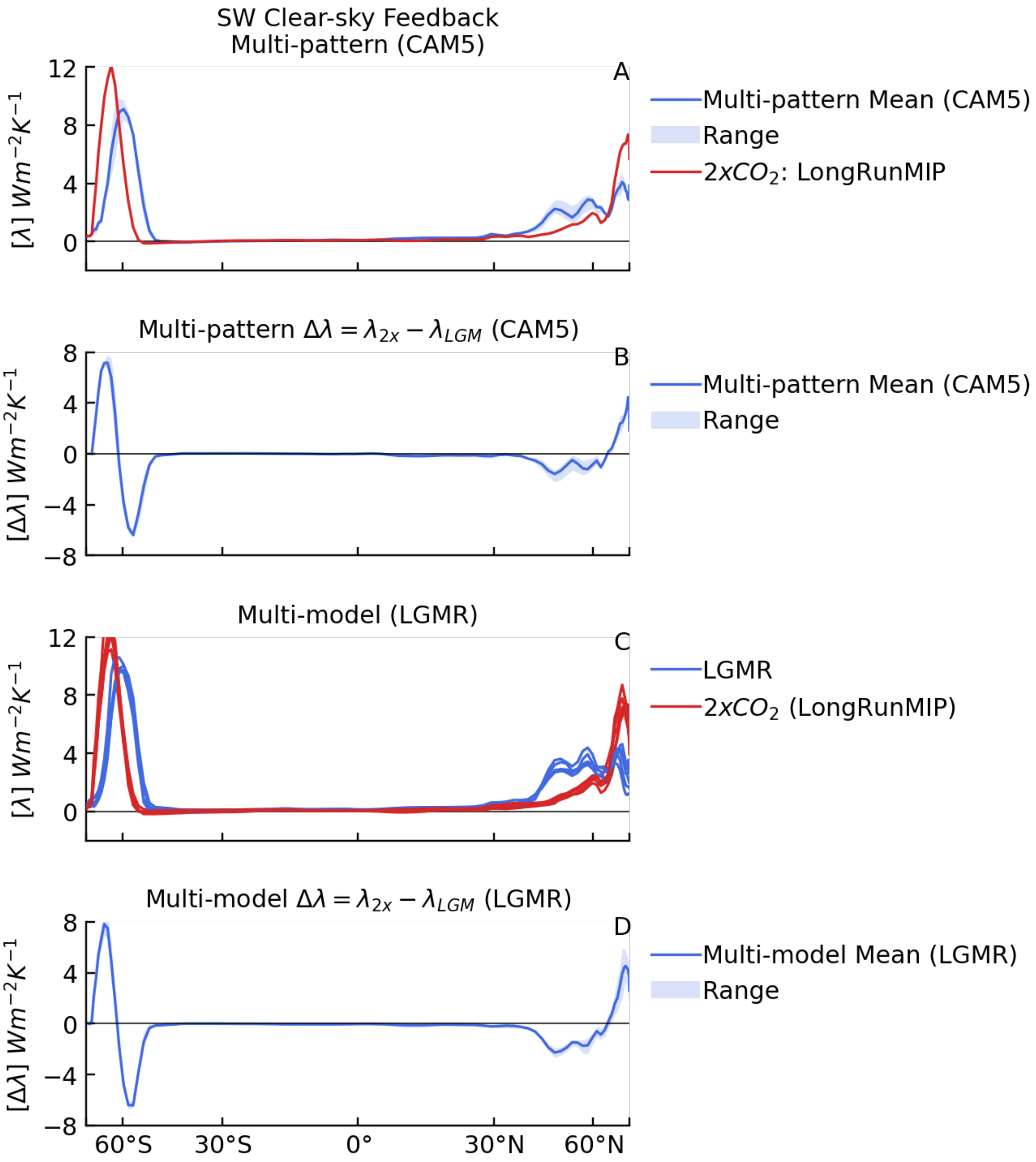

Fig. S13. Zonal-mean shortwave clear-sky feedback and Δλ.

(**A**) In CAM5, mean and range of feedbacks across four LGM reconstructions and $2xCO_2$ from LongRunMIP. (**B**) In CAM5, mean and range of the difference in feedbacks ($\Delta\lambda = \lambda_{2x} - \lambda_{LGM}$) across four LGM reconstructions from results in (A). (**C**) Feedbacks across various AGCMs, using the LGMR reconstruction of the LGM and $2xCO_2$ from LongRunMIP. (**D**) Mean and range of Δλ across various AGCMs from results in (C). Note that HadGEM3 is not included in the kernel-derived feedbacks due to limited model output.

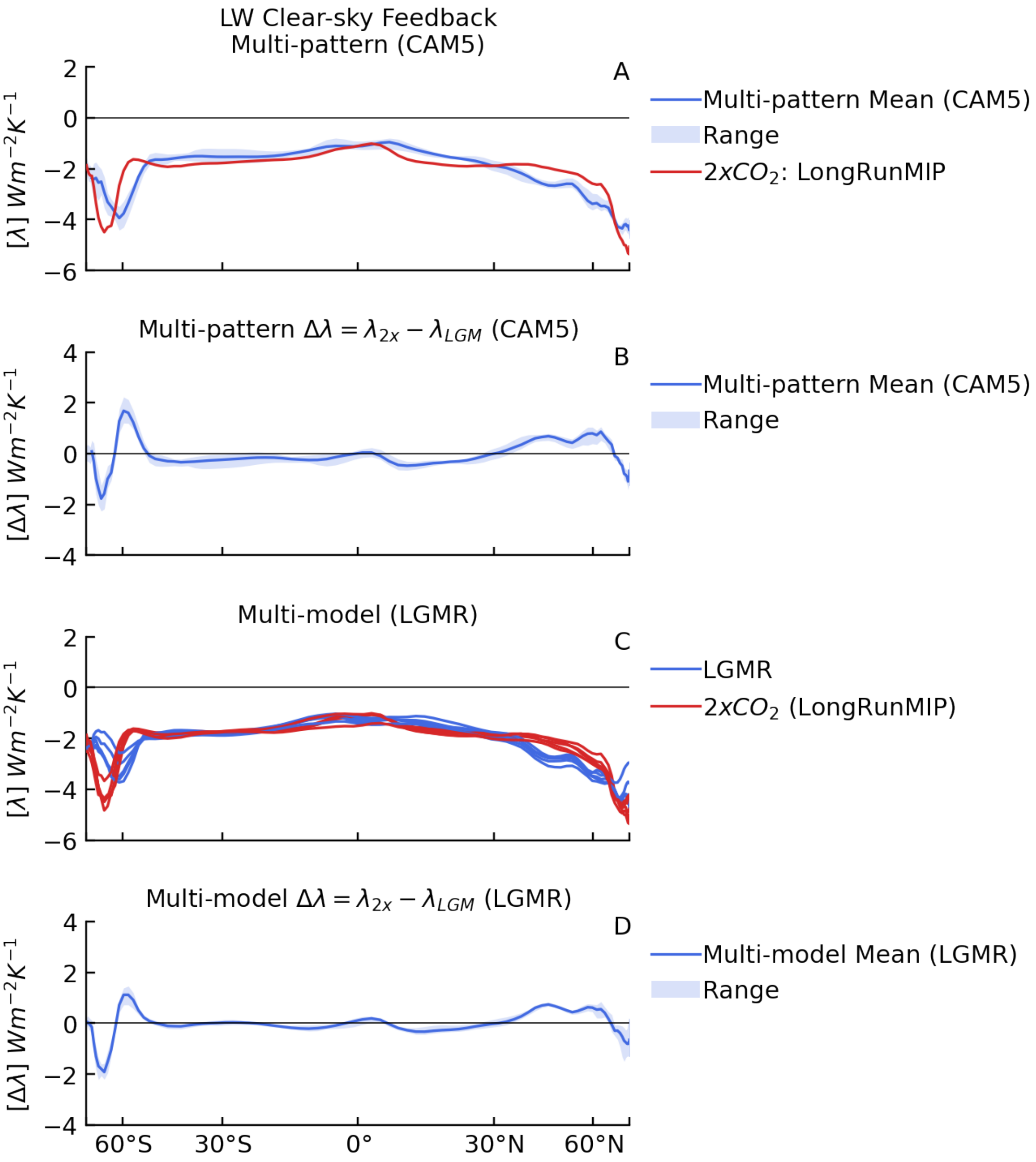

Fig. S14. Zonal-mean longwave clear-sky feedback and Δλ.

(**A**) In CAM5, mean and range of feedbacks across four LGM reconstructions and $2xCO_2$ from LongRunMIP. (**B**) In CAM5, mean and range of the difference in feedbacks ($\Delta\lambda = \lambda_{2x} - \lambda_{LGM}$) across four LGM reconstructions from results in (A). (**C**) Feedbacks across various AGCMs, using the LGMR reconstruction of the LGM and $2xCO_2$ from LongRunMIP. (**D**) Mean and range of Δλ across various AGCMs from results in (C). Note that HadGEM3 is not included in the kernel-derived feedbacks due to limited model output.

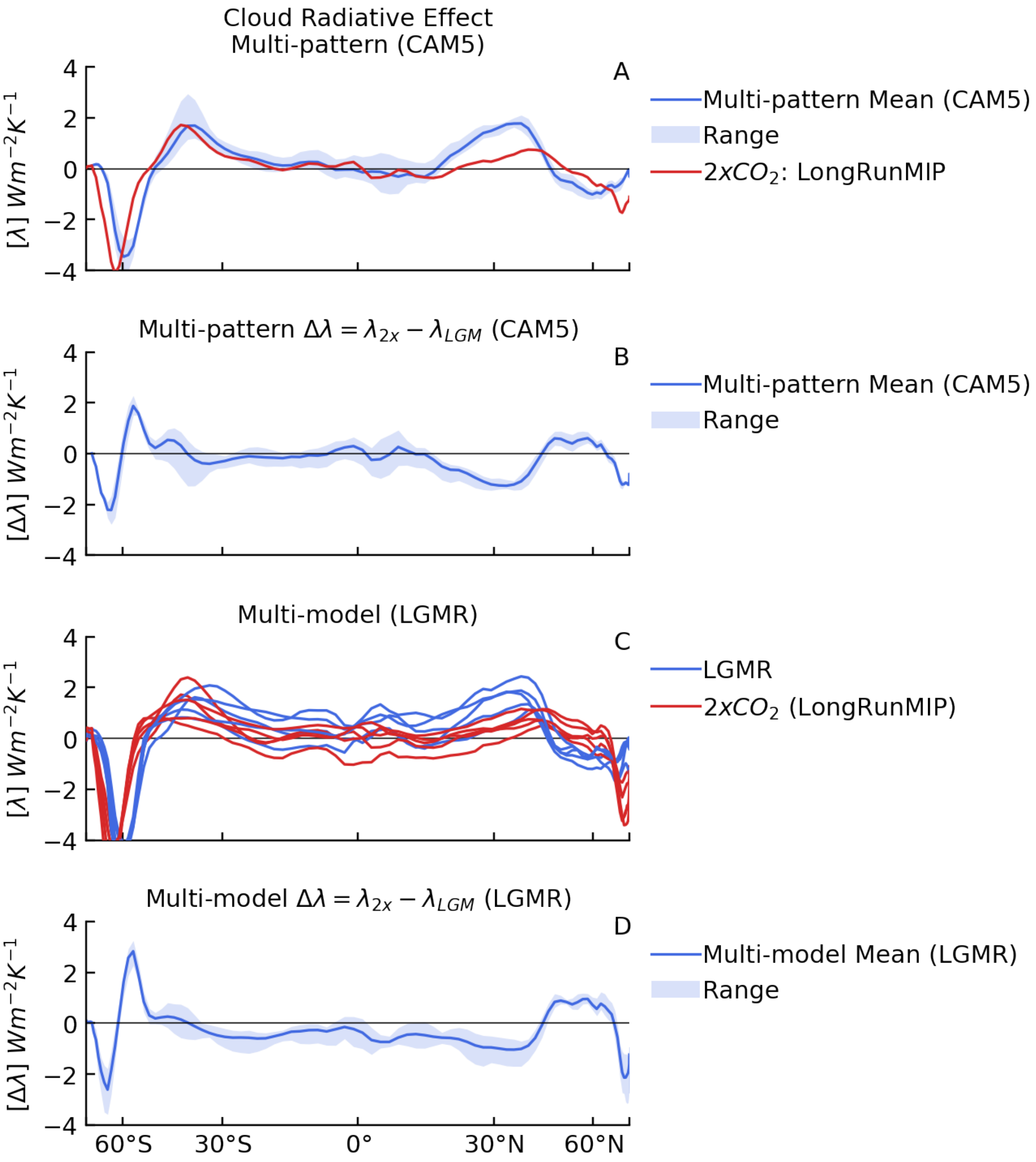

Fig. S15. Zonal-mean cloud radiative effect and Δλ.

(**A**) In CAM5, mean and range of feedbacks across four LGM reconstructions and $2xCO_2$ from LongRunMIP. (**B**) In CAM5, mean and range of the difference in feedbacks ($\Delta\lambda = \lambda_{2x} - \lambda_{LGM}$) across four LGM reconstructions from results in (A). (**C**) Feedbacks across various AGCMs, using the LGMR reconstruction of the LGM and $2xCO_2$ from LongRunMIP. (**D**) Mean and range of Δλ across various AGCMs from results in (C). Note that HadGEM3 is not included in the kernel-derived feedbacks due to limited model output.

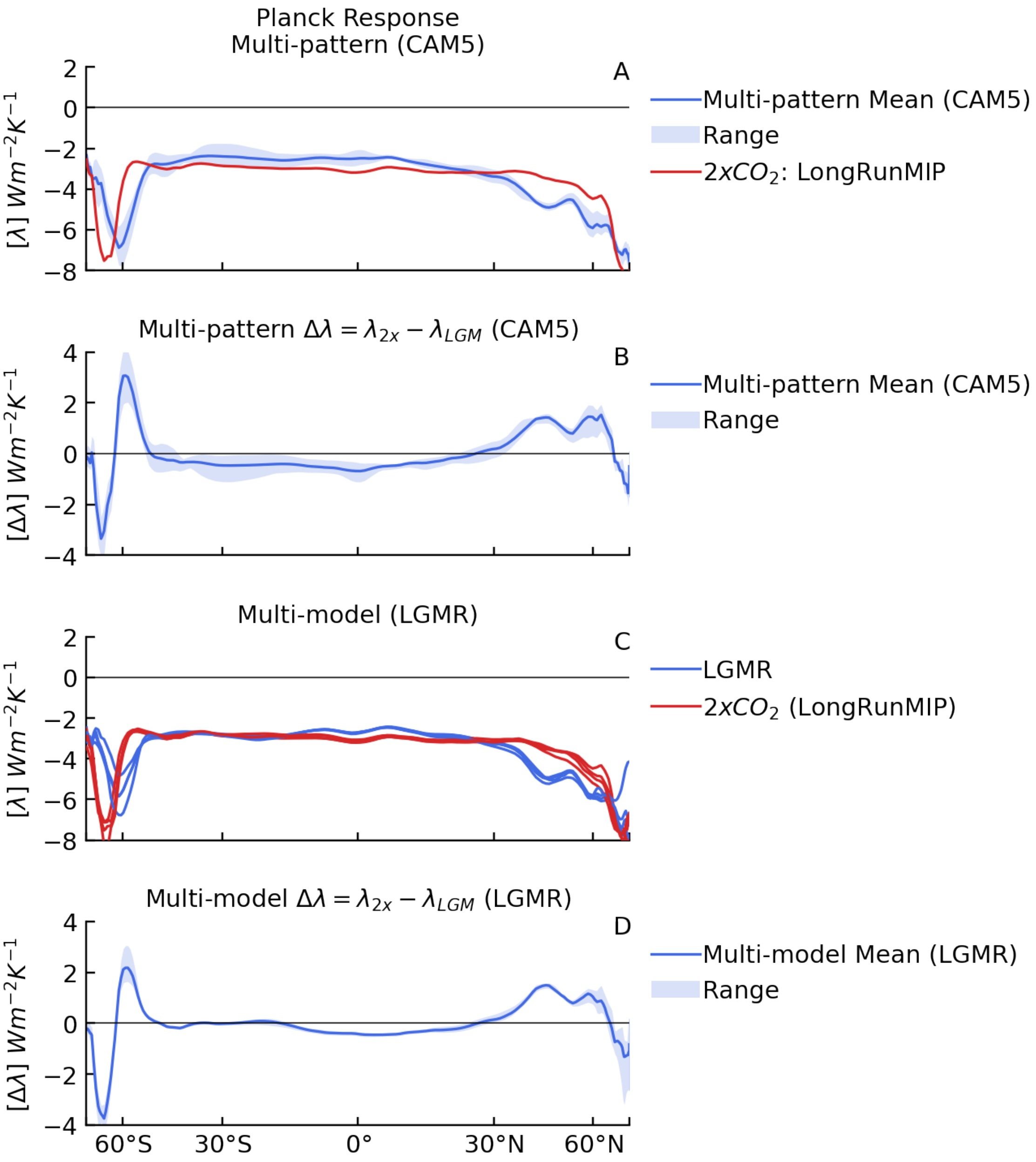

Fig. S16. Zonal-mean Planck response and Δλ.

(**A**) In CAM5, mean and range of feedbacks across four LGM reconstructions and $2xCO_2$ from LongRunMIP. (**B**) In CAM5, mean and range of the difference in feedbacks ($\Delta\lambda = \lambda_{2x} - \lambda_{LGM}$) across four LGM reconstructions from results in (A). (**C**) Feedbacks across various AGCMs, using the LGMR reconstruction of the LGM and $2xCO_2$ from LongRunMIP. (**D**) Mean and range of Δλ across various AGCMs from results in (C). Note that HadGEM3 is not included in the kernel-derived feedbacks due to limited model output.

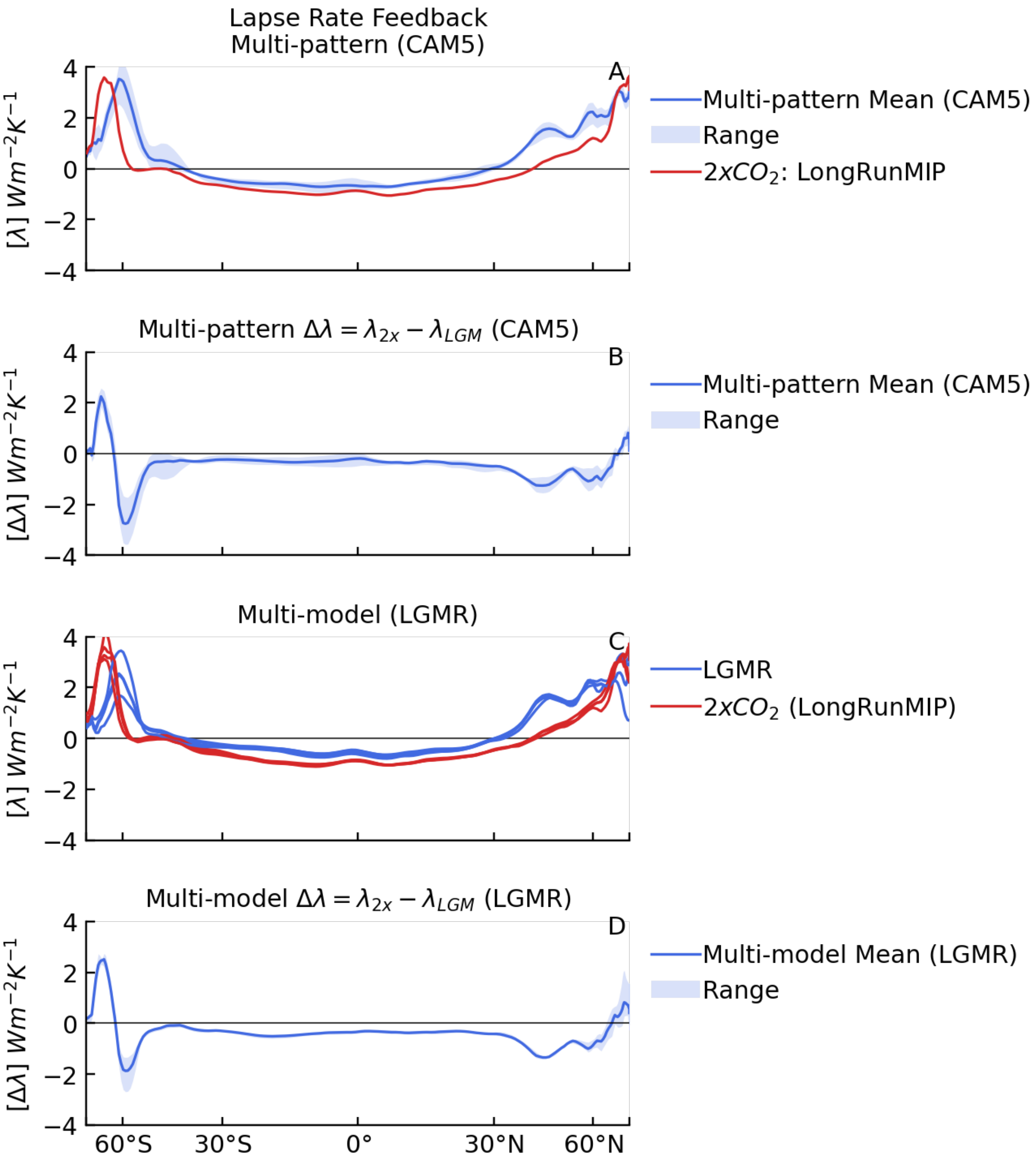

Fig. S17. Zonal-mean lapse rate feedback and Δλ.

(**A**) In CAM5, mean and range of feedbacks across four LGM reconstructions and $2xCO_2$ from LongRunMIP. (**B**) In CAM5, mean and range of the difference in feedbacks ($\Delta\lambda = \lambda_{2x} - \lambda_{LGM}$) across four LGM reconstructions from results in (A). (**C**) Feedbacks across various AGCMs, using the LGMR reconstruction of the LGM and $2xCO_2$ from LongRunMIP. (**D**) Mean and range of Δλ across various AGCMs from results in (C). Note that HadGEM3 is not included in the kernel-derived feedbacks due to limited model output.

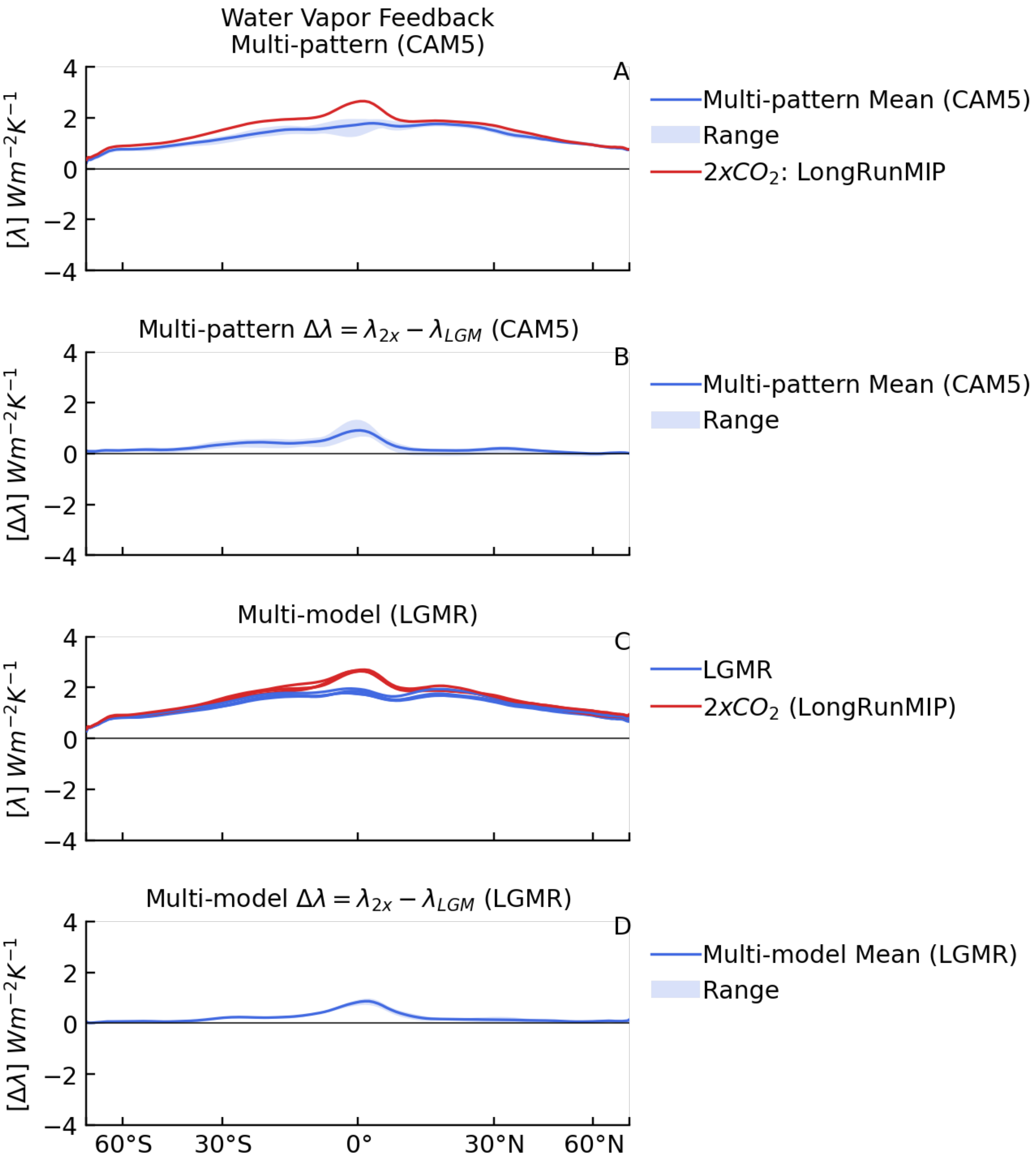

Fig. S18. Zonal-mean water vapor feedback and Δλ.

(**A**) In CAM5, mean and range of feedbacks across four LGM reconstructions and $2xCO_2$ from LongRunMIP. (**B**) In CAM5, mean and range of the difference in feedbacks ($\Delta\lambda = \lambda_{2x} - \lambda_{LGM}$) across four LGM reconstructions from results in (A). (**C**) Feedbacks across various AGCMs, using the LGMR reconstruction of the LGM and $2xCO_2$ from LongRunMIP. (**D**) Mean and range of Δλ across various AGCMs from results in (C). Note that HadGEM3 is not included in the kernel-derived feedbacks due to limited model output.

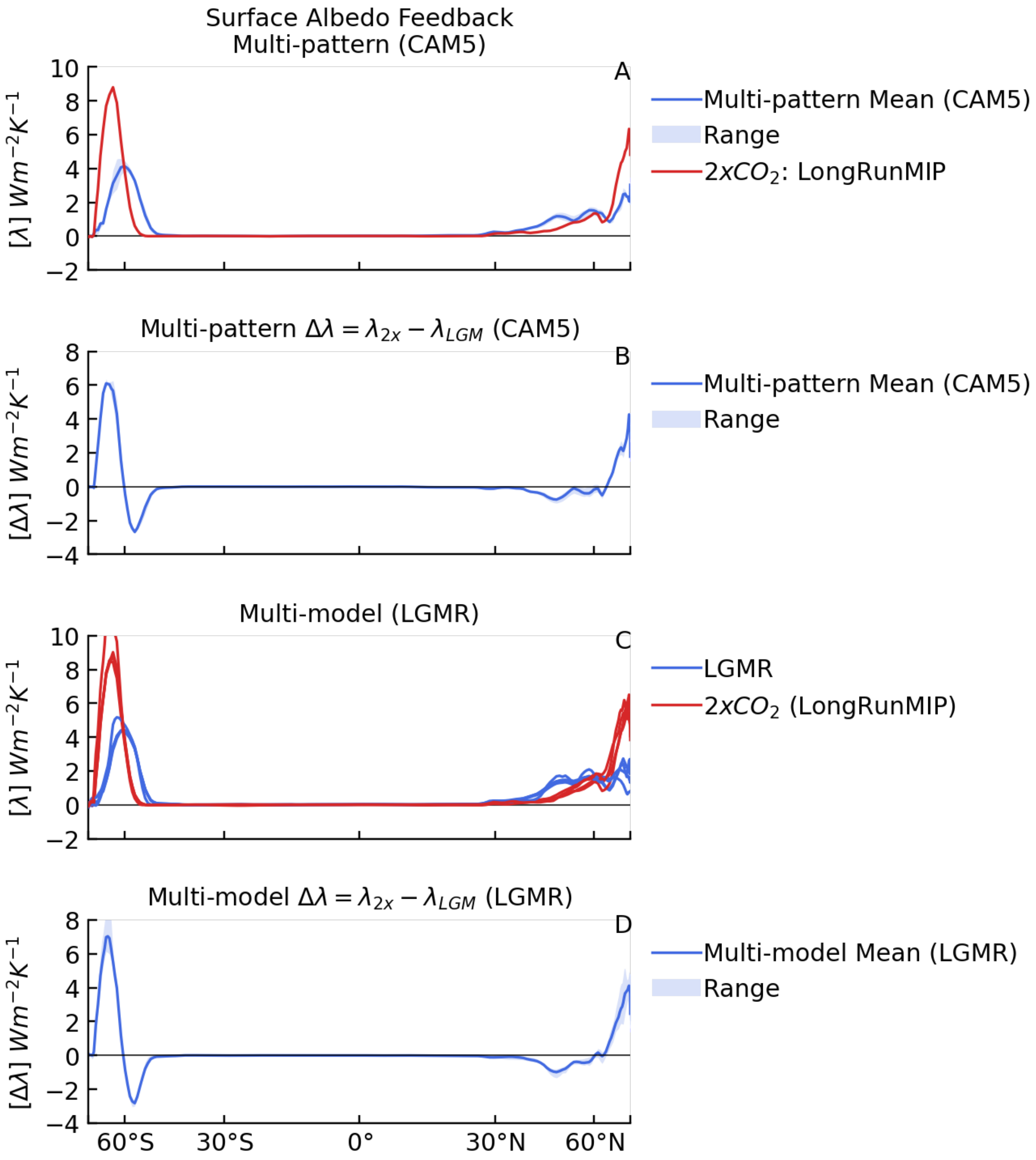

Fig. S19. Zonal-mean surface albedo feedback and Δλ.

(**A**) In CAM5, mean and range of feedbacks across four LGM reconstructions and $2xCO_2$ from LongRunMIP. (**B**) In CAM5, mean and range of the difference in feedbacks ($\Delta\lambda = \lambda_{2x} - \lambda_{LGM}$) across four LGM reconstructions from results in (A). (**C**) Feedbacks across various AGCMs, using the LGMR reconstruction of the LGM and $2xCO_2$ from LongRunMIP. (**D**) Mean and range of Δλ across various AGCMs from results in (C). Note that HadGEM3 is not included in the kernel-derived feedbacks due to limited model output.

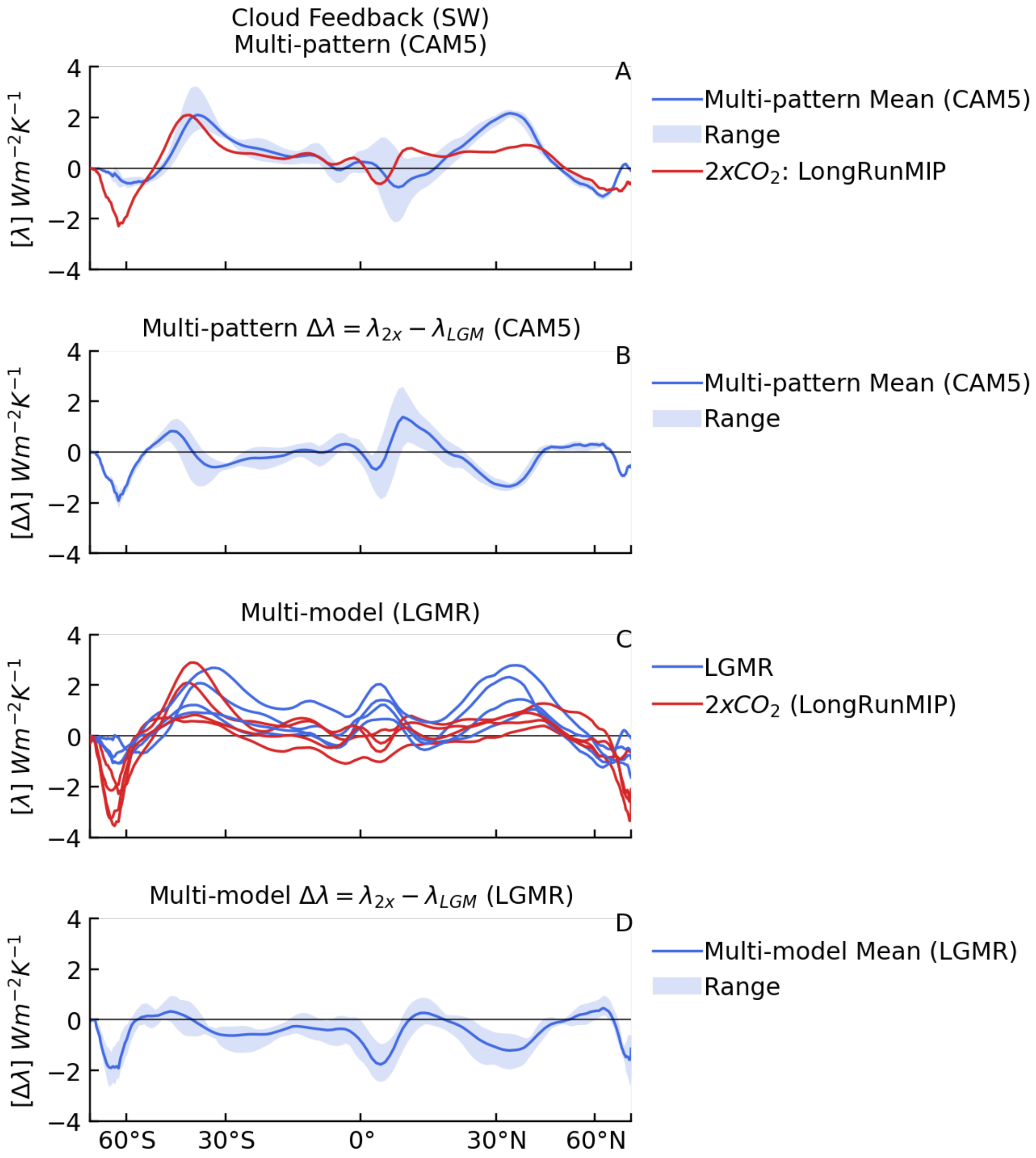

Fig. S20. Zonal-mean shortwave cloud feedback and Δλ.

(**A**) In CAM5, mean and range of feedbacks across four LGM reconstructions and $2xCO_2$ from LongRunMIP. (**B**) In CAM5, mean and range of the difference in feedbacks ($\Delta\lambda = \lambda_{2x} - \lambda_{LGM}$) across four LGM reconstructions from results in (A). (**C**) Feedbacks across various AGCMs, using the LGMR reconstruction of the LGM and $2xCO_2$ from LongRunMIP. (**D**) Mean and range of Δλ across various AGCMs from results in (C). Note that HadGEM3 is not included in the kernel-derived feedbacks due to limited model output.

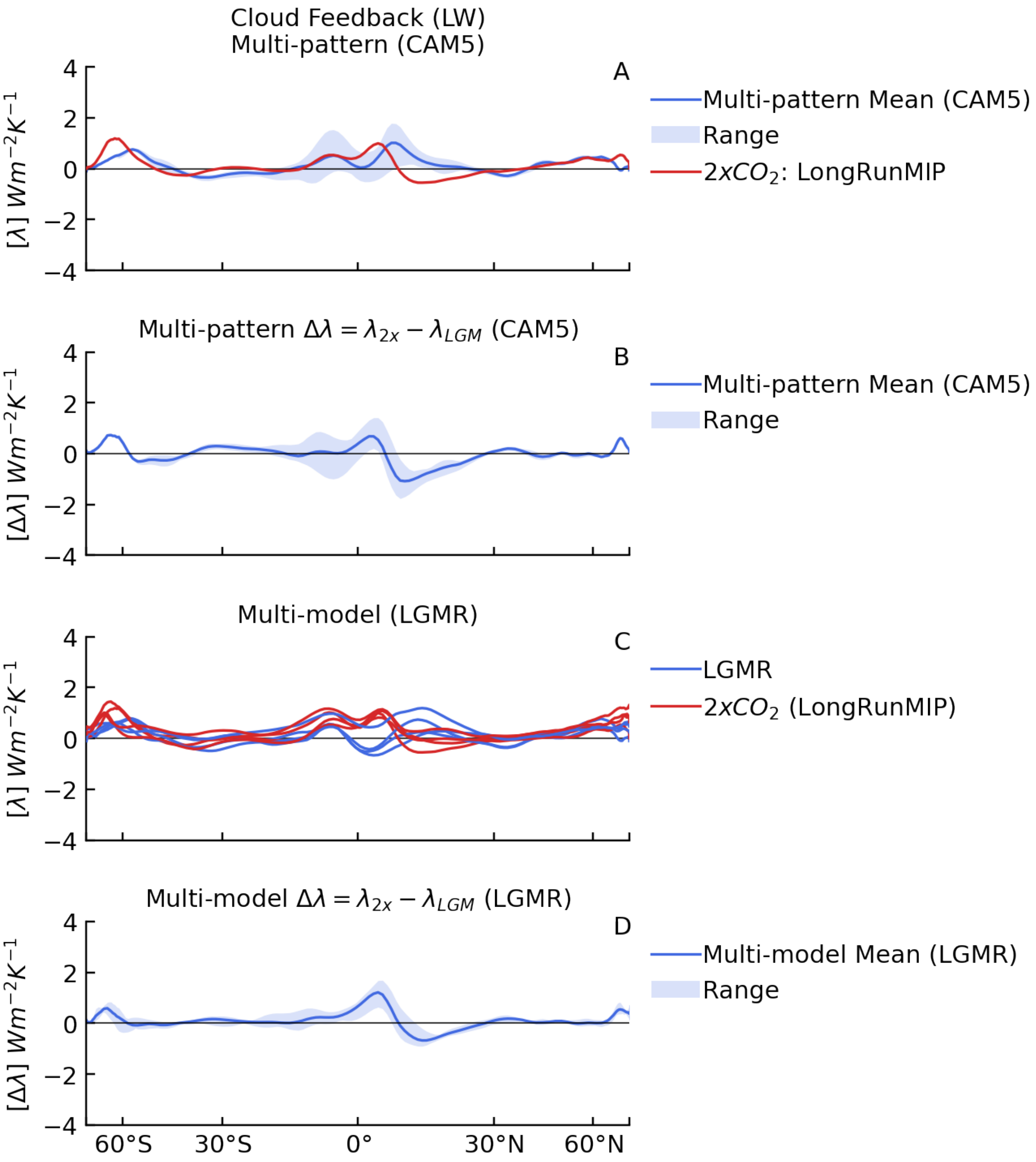

Fig. S21. Zonal-mean longwave cloud feedback and Δλ.

(**A**) In CAM5, mean and range of feedbacks across four LGM reconstructions and $2xCO_2$ from LongRunMIP. (**B**) In CAM5, mean and range of the difference in feedbacks ($\Delta\lambda = \lambda_{2x} - \lambda_{LGM}$) across four LGM reconstructions from results in (A). (**C**) Feedbacks across various AGCMs, using the LGMR reconstruction of the LGM and $2xCO_2$ from LongRunMIP. (**D**) Mean and range of Δλ across various AGCMs from results in (C). Note that HadGEM3 is not included in the kernel-derived feedbacks due to limited model output.

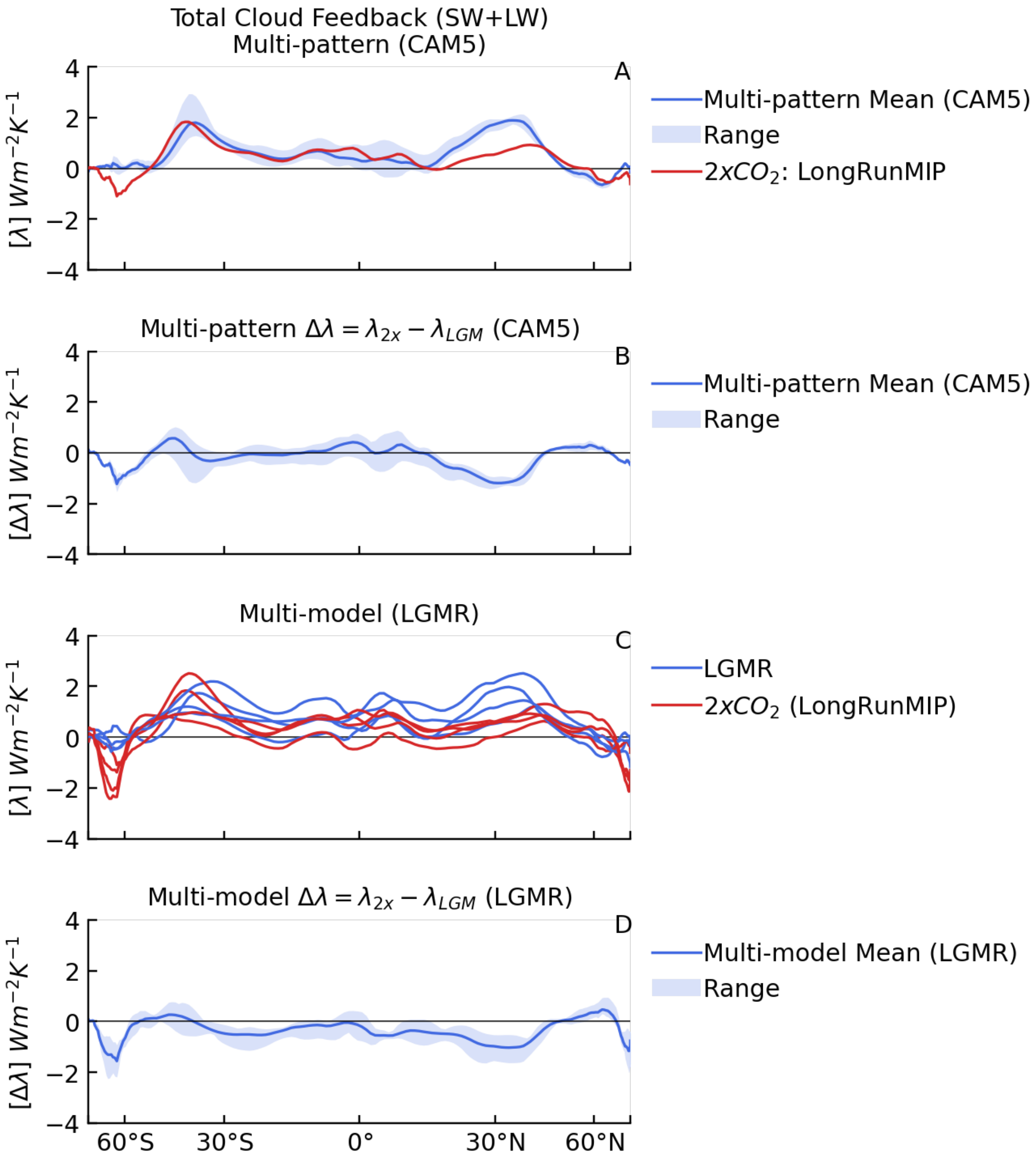

Fig. S22. Zonal-mean total (shortwave + longwave) cloud feedback and Δλ.
(**A**) In CAM5, mean and range of feedbacks across four LGM reconstructions and $2xCO_2$ from LongRunMIP. (**B**) In CAM5, mean and range of the difference in feedbacks ($\Delta\lambda = \lambda_{2x} - \lambda_{LGM}$) across four LGM reconstructions from results in (A). (**C**) Feedbacks across various AGCMs, using the LGMR reconstruction of the LGM and $2xCO_2$ from LongRunMIP. (**D**) Mean and range of Δλ across various AGCMs from results in (C). Note that HadGEM3 is not included in the kernel-derived feedbacks due to limited model output.

Table S1. LGM pattern effect and climate feedbacks in various AGCMs.

[$Wm^{-2}K^{-1}$]	$\Delta\lambda=\lambda_{2x}-\lambda_{LGM}$	λ_{2x} LongRunMIP	λ_{LGM} LGMR	$\Delta\lambda=\lambda_{4x(150yr)}/(1+\zeta)-\lambda_{LGM}$	$\lambda_{4x(150yr)}$	$\varepsilon=\lambda_{2x}/\lambda_{LGM}$ *[unitless]*
**CAM4**	−0.45	−1.47	−1.02	−0.14	−1.23	1.44
**CAM5**	−0.31	−1.05	−0.74	−0.35	−1.15	1.42
**CAM6**	−0.63	−0.83	−0.19	−0.43	−0.66	4.37
**GFDL-AM4**	−0.33	−0.92	−0.60	−0.22	−0.86	1.53
**HadGEM3-GC3.1-LL**	−0.27	−0.62	−0.34	−0.25	−0.63	1.82
**Mean**	**−0.40**	**−0.98**	**−0.58**	**−0.28**	**−0.91**	**2.12**
Std. Dev.	*0.15*	*0.32*	*0.32*	*0.11*	*0.28*	*1.27*

LGM pattern effect ($\Delta\lambda$) calculated as difference in net feedbacks (λ) from 2xCO_2 and LGM. λ_{2x} is calculated in AGCM simulations with LongRunMIP (*39*) 2xCO_2 pattern of SST/SIC. λ_{LGM} is calculated in AGCM simulations with LGMR (*32*) pattern. Alternative values for ($\Delta\lambda$) are shown using 150-year regression of abrupt-4xCO_2 from coupled models corresponding to each AGCM (*17*). ζ is assumed to be 0.06 based on WCRP20's central estimate (*1*). Efficacy, ε, shown in right column. Note that CAM6 is an outlier in efficacy calculations.

Table S2. LGM pattern effect and climate feedbacks from various SST patterns.

	**$\Delta\lambda=\lambda_{2x}-\lambda_{LGM}$** *$Wm^{-2}K^{-1}$*	**λ** *$Wm^{-2}K^{-1}$*	**$\Delta\overline{SST}$** *K*	**$\Delta\overline{T}$** *K*	**$\Delta\overline{N}$** *Wm^{-2}*	**$\Delta\lambda=\lambda_{4x(150yr)}/(1+\zeta)-\lambda_{LGM}$** *$Wm^{-2}K^{-1}$*	**$\varepsilon=\lambda_{2x}/\lambda_{LGM}$**
**CAM4**							
LGMR	−0.45	−1.02	−3.79	−5.06	5.14	−0.14	1.44
lgmDA	−0.69	−0.78	−3.14	−4.16	3.24	−0.38	1.88
Amrhein	−0.48	−0.99	−2.21	−3.38	3.36	−0.17	1.48
Annan	−0.29	−1.17	−2.18	−3.36	3.95	0.01	1.26
**$Mean_{CAM4}$**	**−0.48**	**−0.99**	**−2.83**	**−3.99**	**3.92**	**−0.17**	**1.52**
$StdDev_{CAM4}$	*0.16*	*0.16*	*0.78*	*0.80*	*0.87*	*0.16*	*0.26*
$2xCO_2$	—	*−1.47*	*2.35*	*3.08*	*−4.52*	—	—
**CAM5**							
LGMR	−0.31	−0.74	−3.79	−5.15	3.81	−0.35	1.42
lgmDA	−0.51	−0.54	−3.14	−4.24	2.27	−0.55	1.94
Amrhein	−0.33	−0.72	−2.21	−3.40	2.44	−0.37	1.46
Annan	−0.09	−0.97	−2.18	−3.38	3.28	−0.11	1.08
**$Mean_{CAM5}$**	**−0.31**	**−0.74**	**−2.83**	**−4.05**	**2.95**	**−0.34**	**1.48**
$StdDev_{CAM5}$	*0.18*	*0.18*	*0.78*	*0.84*	*0.72*	*0.18*	*0.35*
$2xCO_2$	—	*−1.05*	*2.35*	*3.09*	*−3.24*	—	—
**$Mean_{CAM4\&5}$**	**−0.39**	**−0.86**	**−2.83**	**−4.01**	**3.41**	**−0.26**	**1.50**
$StdDev_{CAM4\&5}$	*0.21*	*0.21*	*0.72*	*0.76*	*0.90*	*0.18*	*0.29*

LGM pattern effect ($\Delta\lambda$) from net feedbacks (λ) in $2xCO_2$ and with various LGM patterns of SST/SIC. λ_{2x} is calculated in AGCMs with LongRunMIP (*39*) $2xCO_2$ pattern of SST/SIC. λ_{LGM} is calculated in AGCM simulations with four LGM patterns. Global-mean anomalies for SST, near-surface air temperature (T), and top-of-atmosphere radiative imbalance (N) are shown for reference. Values for LGM pattern effect are also shown using 150-year regression of abrupt-$4xCO_2$ from coupled models (*17*). ζ is assumed to be 0.06 based on WCRP20 central estimate (*1*). Efficacy, ε, shown in right column.

Table S3. Climate feedbacks and temperature dependence from pattern-only simulations.

$Wm^{-2}K^{-1}$	$\boldsymbol{\lambda_{2x}^{-0.5K}}$	$\boldsymbol{\lambda_{LGM}^{-0.5K}}$	$\boldsymbol{\Delta\lambda_{Only}^{Pattern} = \lambda_{2x}^{-0.5K} - \lambda_{LGM}^{-0.5K}}$	$\boldsymbol{\Delta\lambda_T = \Delta\lambda - \Delta\lambda_{Only}^{Pattern}}$	$\boldsymbol{\Delta\lambda = \Delta\lambda_{Only}^{Pattern} + \Delta\lambda_T}$, $\boldsymbol{\Delta\lambda = \lambda_{2x} - \lambda_{LGM}}$
**CAM4**	−1.98	−1.55	−0.42	−0.03	−0.45
**CAM5**	−1.59	−1.24	−0.35	0.04	−0.31
**CAM6**	−1.30	−0.55	−0.75	0.12	−0.63
**Mean**	**−1.63**	**−1.12**	**−0.51**	**0.04**	**−0.47**

$\Delta\lambda_{PatternOnly}$ from pattern-only simulations, where LongRunMIP (*39*) $2xCO_2$ and LGMR (*32*) patterns of SST anomalies are scaled to global-mean ΔSST of −0.5 K. Feedback dependence on global-mean temperature ($\Delta\lambda_T$) is estimated as the residual between Δλ in main simulations and $\Delta\lambda_{PatternOnly}$, i.e., assuming $\Delta\lambda=\Delta\lambda_{PatternOnly}+\Delta\lambda_T$. Note that total $\Delta\lambda=\lambda_{2x}-\lambda_{LGM}$.

Table S4. Summary Statistics for Posterior PDFs of Climate Sensitivity.

**PDF from combined lines of evidence (units, K)**	5th %	17th %	50th %	83rd %	95th %	Mean
Assuming ΔT_{LGM} ~ N(μ = –5.0, σ = 1.0) K, as in WCRP20						
WCRP20 Baseline (uniform-λ prior)	2.3	2.6	3.1	3.9	4.7	3.2
**… with Revised $\Delta\lambda_{LGM}$ ~ *N*(–0.37, 0.23) $Wm^{-2}K^{-1}$**	2.1	2.3	2.8	3.4	4.0	2.9
… … and 2x uncertainty, $\Delta\lambda_{LGM}$ ~ *N*(–0.37, 0.46) $Wm^{-2}K^{-1}$	2.1	2.4	2.9	3.6	4.3	3.0
… … based on $\lambda_{4x150yr}$, $\Delta\lambda_{LGM}$ ~ *N*(–0.27, 0.20) $Wm^{-2}K^{-1}$	2.1	2.4	2.8	3.4	4.0	2.9
WCRP20 (uniform-*S* prior)	2.4	2.8	3.5	4.5	5.7	3.7
**... with Revised $\Delta\lambda_{LGM}$ ~ *N*(–0.37, 0.23) $Wm^{-2}K^{-1}$**	2.2	2.5	3.0	3.8	4.6	3.2
… … and 2x uncertainty, $\Delta\lambda_{LGM}$ ~ *N*(–0.37, 0.46) $Wm^{-2}K^{-1}$	2.3	2.6	3.2	4.1	5.1	3.4
… … based on $\lambda_{4x150yr}$, $\Delta\lambda_{LGM}$ ~ *N*(–0.27, 0.20) $Wm^{-2}K^{-1}$	2.2	2.5	3.1	3.8	4.6	3.2
Assuming ΔT_{LGM} ~ N(–6.0, 1.0) K						
WCRP20 Baseline (uniform-λ prior)	2.3	2.7	3.2	4.1	5.0	3.4
**... with Revised $\Delta\lambda_{LGM}$ ~ *N*(–0.37, 0.23) $Wm^{-2}K^{-1}$**	2.1	2.4	2.9	3.5	4.1	3.0
… … and 2x uncertainty, $\Delta\lambda_{LGM}$ ~ *N*(–0.37, 0.46) $Wm^{-2}K^{-1}$	2.2	2.5	3.0	3.7	4.4	3.1
… … based on $\lambda_{4x150yr}$, $\Delta\lambda_{LGM}$ ~ *N*(–0.27, 0.20) $Wm^{-2}K^{-1}$	2.2	2.4	2.9	3.5	4.2	3.0
WCRP20 (uniform-*S* prior)	2.5	2.9	3.7	4.8	6.1	3.9
**... with Revised $\Delta\lambda_{LGM}$ ~ *N*(–0.37, 0.23) $Wm^{-2}K^{-1}$**	2.3	2.6	3.1	3.9	4.7	3.3
… … and 2x uncertainty, $\Delta\lambda_{LGM}$ ~ *N*(–0.37, 0.46) $Wm^{-2}K^{-1}$	2.3	2.7	3.3	4.3	5.3	3.5
… … based on $\lambda_{4x150yr}$, $\Delta\lambda_{LGM}$ ~ *N*(–0.27, 0.20) $Wm^{-2}K^{-1}$	2.3	2.6	3.2	4.0	4.8	3.3
**PDF from LGM evidence alone (uniform-*S* prior)**	5th %	17th %	50th %	83rd %	95th %	Mean
Assuming ΔT_{LGM} ~ N(–5.0, 1.0) K as in WCRP20						
WCRP20	1.7	2.4	4.5	10.6	16.5	6.2
... with Revised $\Delta\lambda_{LGM}$ ~ *N*(–0.37, 0.23) $Wm^{-2}K^{-1}$	1.1	1.5	2.1	3.0	4.2	2.3
… … and 2x uncertainty, $\Delta\lambda_{LGM}$ ~ *N*(–0.37, 0.46) $Wm^{-2}K^{-1}$	1.2	1.5	2.4	4.7	10.0	3.4
Assuming ΔT_{LGM} ~ N(–6.0, 1.0) K						
WCRP20	2.3	3.4	6.8	13.9	18.0	8.2
... with Revised $\Delta\lambda_{LGM}$ ~ *N*(–0.37, 0.23) $Wm^{-2}K^{-1}$	1.4	1.7	2.4	3.5	5.0	2.7
… … and 2x uncertainty, $\Delta\lambda_{LGM}$ ~ *N*(–0.37, 0.46) $Wm^{-2}K^{-1}$	1.4	1.9	3.0	6.4	13.0	4.3

Note: The posterior PDF from LGM evidence alone uses the uniform-*S* prior (0, 20) K, hence the shape of the posterior PDF matches that of the LGM likelihood. Methods follow WCRP20 (*1*).

REFERENCES

1. S. C. Sherwood, M. J. Webb, J. D. Annan, K. C. Armour, P. M. Forster, J. C. Hargreaves, G. Hegerl, S. A. Klein, K. D. Marvel, E. J. Rohling, M. Watanabe, T. Andrews, P. Braconnot, C. S. Bretherton, G. L. Foster, Z. Hausfather, A. S. von der Heydt, R. Knutti, T. Mauritsen, J. R. Norris, C. Proistosescu, M. Rugenstein, G. A. Schmidt, K. B. Tokarska, M. D. Zelinka, An assessment of Earth's climate sensitivity using multiple lines of evidence. *Rev. Geophys.* **58**, e2019RG000678 (2020).

2. P. Forster, T. Storelvmo, K. Armour, W. Collins, J.-L. Dufresne, D. Frame, D. J. Lunt, T. Mauritsen, M. D. Palmer, M. Watanabe, M. Wild, H. Zhang, 2021: The Earth's energy budget, climate feedbacks, and climate sensitivity in *Climate Change 2021: The Physical Science Basis. Contribution of Working Group I to the Sixth Assessment Report of the Intergovernmental Panel on Climate Change*, V. Masson-Delmotte, P. Zhai, A. Pirani, S. L. Connors, C. Péan, S. Berger, N. Caud, Y. Chen, L. Goldfarb, M. I. Gomis, M. Huang, K. Leitzell, E. Lonnoy, J. B. R. Matthews, T. K. Maycock, T. Waterfield, O. Yelekçi, R. Yu, B. Zhou, Eds. (Cambridge Univ. Press, 2021).

3. J. E. Tierney, J. Zhu, J. King, S. B. Malevich, G. J. Hakim, C. J. Poulsen, Glacial cooling and climate sensitivity revisited. *Nature* **584**, 569–573 (2020).

4. S. Manabe, K. Bryan, CO2-induced change in a coupled ocean-atmosphere model and its paleoclimatic implications. *J. Geophys. Res.* **90**, 11689–11707 (1985).

5. PALAEOSENS project members, Making sense of palaeoclimate sensitivity. *Nature* **491**, 683–691 (2012).

6. P. Köhler, B. de Boer, A. S. von der Heydt, L. B. Stap, R. S. W. van de Wal, On the state dependency of the equilibrium climate sensitivity during the last 5 million years. *Clim. Past* **11**, 1801–1823 (2015).

7. A. S. von der Heydt, H. A. Dijkstra, R. S. W. van de Wal, R. Caballero, M. Crucifix, G. L. Foster, M. Huber, P. Köhler, E. Rohling, P. J. Valdes, P. Ashwin, S. Bathiany, T. Berends, L. G. J. van Bree, P. Ditlevsen, M. Ghil, A. M. Haywood, J. Katzav, G. Lohmann, J. Lohmann, V. Lucarini, A. Marzocchi, H. Pälike, I. R. Baroni, D. Simon, A. Sluijs, L. B. Stap, A. Tantet, J. Viebahn, M. Ziegler, Lessons on climate sensitivity from past climate changes. *Curr. Clim. Change Rep.* **2**, 148–158 (2016).

8. T. Friedrich, A. Timmermann, M. Tigchelaar, O. E. Timm, A. Ganopolski, Nonlinear climate sensitivity and its implications for future greenhouse warming. *Sci. Adv.* **2**, e1501923 (2016).

9. E. J. Rohling, G. Marino, G. L. Foster, P. A. Goodwin, A. S. von der Heydt, P. Köhler, Comparing climate sensitivity, past and present. *Ann. Rev. Mar. Sci.* **10**, 261–288 (2018).

10. K. C. Armour, C. M. Bitz, G. H. Roe, Time-varying climate sensitivity from regional feedbacks. *J. Clim.* **26**, 4518–4534 (2013).

11. C. Zhou, M. D. Zelinka, S. A. Klein, Impact of decadal cloud variations on the Earth's energy budget. *Nat. Geosci.* **9**, 871–874 (2016).

12. Y. Dong, C. Proistosescu, K. C. Armour, D. S. Battisti, Attributing historical and future evolution of radiative feedbacks to regional warming patterns using a Green's function approach: The preeminence of the western Pacific. *J. Clim.* **32**, 5471–5491 (2019).

13. T. Andrews, M. J. Webb, The dependence of global cloud and lapse rate feedbacks on the spatial structure of tropical Pacific warming. *J. Clim.* **31**, 641–654 (2018).

14. S. Fueglistaler, Observational evidence for two modes of coupling between sea surface temperatures, tropospheric temperature profile, and shortwave cloud radiative effect in the tropics. *Geophys. Res. Lett.* **46**, 9890–9898 (2019).

15. P. Ceppi, J. M. Gregory, Relationship of tropospheric stability to climate sensitivity and Earth's observed radiation budget. *Proc. Natl. Acad. Sci. U.S.A.* **114**, 13126–13131 (2017).

16. T. Andrews, J. M. Gregory, D. Paynter, L. G. Silvers, C. Zhou, T. Mauritsen, M. J. Webb, K. C. Armour, P. M. Forster, H. Titchner, Accounting for changing temperature patterns increases historical estimates of climate sensitivity. *Geophys. Res. Lett.* **45**, 8490–8499 (2018).

17. T. Andrews, A. Bodas-Salcedo, J. M. Gregory, Y. Dong, K. C. Armour, D. Paynter, P. Lin, A. Modak, T. Mauritsen, J. N. S. Cole, B. Medeiros, J. J. Benedict, H. Douville, R. Roehrig, T. Koshiro, H. Kawai, T. Ogura, J.-L. Dufresne, R. P. Allan, C. Liu, On the effect of historical SST patterns on radiative feedback. *J. Geophys. Res. Atmos.* **127**, e2022JD036675 (2022).

18. C. Zhou, M. D. Zelinka, S. A. Klein, Analyzing the dependence of global cloud feedback on the spatial pattern of sea surface temperature change with a Green's function approach. *J. Adv. Model. Earth Syst.* **9**, 2174–2189 (2017).

19. K. C. Armour, J. Marshall, J. R. Scott, A. Donohoe, E. R. Newsom, Southern Ocean warming delayed by circumpolar upwelling and equatorward transport. *Nat. Geosci.* **9**, 549–554 (2016).

20. Y. Dong, K. C. Armour, M. D. Zelinka, C. Proistosescu, D. S. Battisti, C. Zhou, T. Andrews, Intermodel spread in the pattern effect and its contribution to climate sensitivity in CMIP5 and CMIP6 models. *J. Clim.* **33**, 7755–7775 (2020).

21. C. Proistosescu, P. J. Huybers, Slow climate mode reconciles historical and model-based estimates of climate sensitivity. *Sci. Adv.* **3**, e1602821 (2017).

22. M. Renoult, N. Sagoo, J. Zhu, T. Mauritsen, Causes of the weak emergent constraint on climate sensitivity at the Last Glacial Maximum. *Clim. Past* **19**, 323–356 (2023).

23. J. Zhu, C. J. Poulsen, Last Glacial Maximum (LGM) climate forcing and ocean dynamical feedback and their implications for estimating climate sensitivity. *Clim Past* **17**, 253–267 (2021).

24. P. Braconnot, M. Kageyama, Shortwave forcing and feedbacks in Last Glacial Maximum and Mid-Holocene PMIP3 simulations. *Philos. Trans. R. Soc. A: Math. Phys. Eng. Sci.* **373**, 20140424 (2015).

25. S. Manabe, A. J. Broccoli, The influence of continental ice sheets on the climate of an ice age. *J. Geophys. Res.* **90**, 2167–2190 (1985).

26. K. H. Cook, I. M. Held, Stationary waves of the ice age climate. *J. Clim.* **1**, 807–819 (1988).

27. S.-Y. Lee, J. C. H. Chiang, P. Chang, Tropical Pacific response to continental ice sheet topography. *Clim. Dyn.* **44**, 2429–2446 (2015).

28. P. N. DiNezio, J. E. Tierney, B. L. Otto-Bliesner, A. Timmermann, T. Bhattacharya, N. Rosenbloom, E. Brady, Glacial changes in tropical climate amplified by the Indian Ocean. *Sci. Adv.* **4**, eaat9658 (2018).

29. W. H. G. Roberts, C. Li, P. J. Valdes, The mechanisms that determine the response of the Northern Hemisphere's stationary waves to North American ice sheets. *J. Clim.* **32**, 3917–3940 (2019).

30. D. J. Amaya, A. M. Seltzer, K. B. Karnauskas, J. M. Lora, X. Zhang, P. N. DiNezio, Air-sea coupling shapes North American hydroclimate response to ice sheets during the Last Glacial Maximum. *Earth Planet. Sci. Lett.* **578**, 117271 (2022).

31. G. J. Hakim, J. Emile-Geay, E. J. Steig, D. Noone, D. M. Anderson, R. Tardif, N. Steiger, W. A. Perkins, The last millennium climate reanalysis project: Framework and first results. *J. Geophys. Res. Atmos.* **121**, 6745–6764 (2016).

32. M. B. Osman, J. E. Tierney, J. Zhu, R. Tardif, G. J. Hakim, J. King, C. J. Poulsen, Globally resolved surface temperatures since the Last Glacial Maximum. *Nature* **599**, 239–244 (2021).

33. J. D. Annan, J. C. Hargreaves, T. Mauritsen, A new global surface temperature reconstruction for the Last Glacial Maximum. *Clim. Past* **18**, 1883–1896 (2022).

34. D. E. Amrhein, C. Wunsch, O. Marchal, G. Forget, A global Glacial Ocean state estimate constrained by upper-ocean temperature proxies. *J. Clim.* **31**, 8059–8079 (2018).

35. D. E. Amrhein, G. J. Hakim, L. A. Parsons, Quantifying structural uncertainty in paleoclimate data assimilation with an application to the Last Millennium. *Geophys. Res. Lett.* **47**, e2020GL090485 (2020).

36. L. A. Parsons, D. E. Amrhein, S. C. Sanchez, R. Tardif, M. K. Brennan, G. J. Hakim, Do multi-model ensembles improve reconstruction skill in paleoclimate data assimilation? *Earth Space Sci.* **8**, e2020EA001467 (2021).

37. S. M. Kang, S.-P. Xie, Dependence of climate response on meridional structure of external thermal forcing. *J. Clim.* **27**, 5593–5600 (2014).

38. B. E. J. Rose, K. C. Armour, D. S. Battisti, N. Feldl, D. D. B. Koll, The dependence of transient climate sensitivity and radiative feedbacks on the spatial pattern of ocean heat uptake. *Geophys. Res. Lett.* **41**, 1071–1078 (2014).

39. M. Rugenstein, J. Bloch-Johnson, A. Abe-Ouchi, T. Andrews, U. Beyerle, L. Cao, T. Chadha, G. Danabasoglu, J.-L. Dufresne, L. Duan, M.-A. Foujols, T. Frölicher, O. Geoffroy, J. Gregory, R. Knutti, C. Li, A. Marzocchi, T. Mauritsen, M. Menary, E. Moyer, L. Nazarenko, D.

Paynter, D. Saint-Martin, G. A. Schmidt, A. Yamamoto, S. Yang, LongRunMIP: Motivation and design for a large collection of millennial-length AOGCM simulations. *Bull. Am. Meteorol. Soc.* **100**, 2551–2570 (2019).

40. M. Crucifix, Does the Last Glacial Maximum constrain climate sensitivity? *Geophys. Res. Lett.* **33**, L18701 (2006).

41. M. Yoshimori, J. C. Hargreaves, J. D. Annan, T. Yokohata, A. Abe-Ouchi, Dependency of feedbacks on forcing and climate state in physics parameter ensembles. *J. Clim.* **24**, 6440–6455 (2011).

42. L. B. Stap, P. Köhler, G. Lohmann, Including the efficacy of land ice changes in deriving climate sensitivity from paleodata. *Earth Syst. Dynam.* **10**, 333–345 (2019).

43. J. D. Shakun, Modest global-scale cooling despite extensive early Pleistocene ice sheets. *Quat. Sci. Rev.* **165**, 25–30 (2017).

44. P. O. Hopcroft, P. J. Valdes, How well do simulated Last Glacial Maximum tropical temperatures constrain equilibrium climate sensitivity? *Geophys. Res. Lett.* **42**, 5533–5539 (2015).

45. R. P. Allan, C. Liu, N. G. Loeb, M. D. Palmer, M. Roberts, D. Smith, P.-L. Vidale, Changes in global net radiative imbalance 1985–2012. *Geophys. Res. Lett.* **41**, 5588–5597 (2014).

46. N. G. Loeb, H. Wang, R. P. Allan, T. Andrews, K. Armour, J. N. S. Cole, J.-L. Dufresne, P. Forster, A. Gettelman, H. Guo, T. Mauritsen, Y. Ming, D. Paynter, C. Proistosescu, M. F. Stuecker, U. Willén, K. Wyser, New generation of climate models track recent unprecedented changes in Earth's radiation budget observed by CERES. *Geophys. Res. Lett.* **47**, e2019GL086705 (2020).

47. C. M. Bitz, K. M. Shell, P. R. Gent, D. A. Bailey, G. Danabasoglu, K. C. Armour, M. M. Holland, J. T. Kiehl, Climate sensitivity of the Community Climate System Model, version 4. *J. Clim.* **25**, 3053–3070 (2012).

48. J. Zhu, B. L. Otto-Bliesner, E. C. Brady, C. J. Poulsen, J. E. Tierney, M. Lofverstrom, P. DiNezio, Assessment of equilibrium climate sensitivity of the community Earth System Model version 2 through simulation of the Last Glacial Maximum. *Geophys. Res. Lett.* **48**, e2020GL091220 (2021).

49. J. Zhu, B. L. Otto-Bliesner, E. C. Brady, A. Gettelman, J. T. Bacmeister, R. B. Neale, C. J. Poulsen, J. K. Shaw, Z. S. McGraw, J. E. Kay, LGM paleoclimate constraints inform cloud parameterizations and equilibrium climate sensitivity in CESM2. *J. Adv. Model. Earth Syst.* **14**, e2021MS002776 (2022).

50. G. H. Roe, R. S. Lindzen, The mutual interaction between continental-scale ice sheets and atmospheric stationary waves. *J. Clim.* **14**, 1450–1465 (2001).

51. B. J. Soden, I. M. Held, R. Colman, K. M. Shell, J. T. Kiehl, C. A. Shields, Quantifying climate feedbacks using radiative kernels. *J. Clim.* **21**, 3504–3520 (2008).

52. S. P. Raghuraman, D. Paynter, R. Menzel, V. Ramaswamy, Forcing, cloud feedbacks, cloud masking, and internal variability in the cloud radiative effect satellite record. *J. Clim.* **36**, 4151–4167 (2023).

53. J. Bloch-Johnson, M. Rugenstein, M. B. Stolpe, T. Rohrschneider, Y. Zheng, J. M. Gregory, Climate sensitivity increases under higher CO2 levels due to feedback temperature dependence. *Geophys. Res. Lett.* **48**, e2020GL089074 (2021).

54. J. E. Hansen, M. Sato, L. Simons, L. S. Nazarenko, I. Sangha, P. Kharecha, J. C. Zachos, K. von Schuckmann, N. G. Loeb, M. B. Osman, Q. Jin, G. Tselioudis, E. Jeong, A. Lacis, R. Ruedy,

G. Russell, J. Cao, J. Li, Global warming in the pipeline. *Oxford Open Clim. Change* **3**, kgad008 (2023).

55. J. Hansen, M. Sato, R. Ruedy, L. Nazarenko, A. Lacis, G. A. Schmidt, G. Russell, I. Aleinov, M. Bauer, S. Bauer, N. Bell, B. Cairns, V. Canuto, M. Chandler, Y. Cheng, A. Del Genio, G. Faluvegi, E. Fleming, A. Friend, T. Hall, C. Jackman, M. Kelley, N. Kiang, D. Koch, J. Lean, J. Lerner, K. Lo, S. Menon, R. Miller, P. Minnis, T. Novakov, V. Oinas, Ja. Perlwitz, Ju. Perlwitz, D. Rind, A. Romanou, D. Shindell, P. Stone, S. Sun, N. Tausnev, D. Thresher, B. Wielicki, T. Wong, M. Yao, S. Zhang, Efficacy of climate forcings. *J. Geophys. Res. Atmos.* **110**, D18104 (2005).

56. M. Yoshimori, T. Yokohata, A. Abe-Ouchi, A comparison of climate feedback strength between CO2 doubling and LGM experiments. *J. Climate* **22**, 3374–3395 (2009).

57. M. Kageyama, S. P. Harrison, M.-L. Kapsch, M. Lofverstrom, J. M. Lora, U. Mikolajewicz, S. Sherriff-Tadano, T. Vadsaria, A. Abe-Ouchi, N. Bouttes, D. Chandan, L. J. Gregoire, R. F. Ivanovic, K. Izumi, A. N. LeGrande, F. Lhardy, G. Lohmann, P. A. Morozova, R. Ohgaito, A. Paul, W. R. Peltier, C. J. Poulsen, A. Quiquet, D. M. Roche, X. Shi, J. E. Tierney, P. J. Valdes, E. Volodin, J. Zhu, The PMIP4 Last Glacial Maximum experiments: Preliminary results and comparison with the PMIP3 simulations. *Clim. Past* **17**, 1065–1089 (2021).

58. T. B. Richardson, P. M. Forster, C. J. Smith, A. C. Maycock, T. Wood, T. Andrews, O. Boucher, G. Faluvegi, D. Fläschner, Ø. Hodnebrog, M. Kasoar, A. Kirkevåg, J. -F. Lamarque, J. Mülmenstädt, G. Myhre, D. Olivié, R. W. Portmann, B. H. Samset, D. Shawki, D. Shindell, P. Stier, T. Takemura, A. Voulgarakis, D. Watson-Parris, Efficacy of climate forcings in PDRMIP models. *J. Geophys. Res. Atmos.* **124**, 12824–12844 (2019).

59. C. Zhou, M. Wang, M. D. Zelinka, Y. Liu, Y. Dong, K. C. Armour, Explaining forcing efficacy with pattern effect and state dependence. *Geophys. Res. Lett.* **50**, e2022GL101700 (2023).

60. M. J. Webb, T. Andrews, A. Bodas-Salcedo, S. Bony, C. S. Bretherton, R. Chadwick, H. Chepfer, H. Douville, P. Good, J. E. Kay, S. A. Klein, R. Marchand, B. Medeiros, A. P. Siebesma, C. B. Skinner, B. Stevens, G. Tselioudis, Y. Tsushima, M. Watanabe, The Cloud Feedback Model Intercomparison Project (CFMIP) contribution to CMIP6. *Geosci. Model Dev.* **10**, 359–384 (2017).

61. R. Pincus, P. M. Forster, B. Stevens, The radiative forcing model intercomparison project (RFMIP): Experimental protocol for CMIP6. *Geosci. Model Dev.* **9**, 3447–3460 (2016).

62. A. M. Seltzer, J. Ng, W. Aeschbach, R. Kipfer, J. T. Kulongoski, J. P. Severinghaus, M. Stute, Widespread six degrees celsius cooling on land during the Last Glacial Maximum. *Nature* **593**, 228–232 (2021).

63. Z. Liu, Y. Bao, L. G. Thompson, E. Mosley-Thompson, C. Tabor, G. J. Zhang, M. Yan, M. Lofverstrom, I. Montanez, J. Oster, Tropical mountain ice core δ18O: A Goldilocks indicator for global temperature change. *Sci. Adv.* **9**, eadi6725 (2023).

64. R. Knutti, G. C. Hegerl, The equilibrium sensitivity of the Earth's temperature to radiation changes. *Nat. Geosci.* **1**, 735–743 (2008).

65. MARGO project members, Constraints on the magnitude and patterns of ocean cooling at the Last Glacial Maximum. *Nat. Geosci.* **2**, 127–132 (2009).

66. E. C. Brady, B. L. Otto-Bliesner, J. E. Kay, N. Rosenbloom, Sensitivity to glacial forcing in the CCSM4. *J. Climate* **26**, 1901–1925 (2013).

67. R. B. Neale, J. Richter, S. Park, P. H. Lauritzen, S. J. Vavrus, P. J. Rasch, M. Zhang, The mean climate of the Community Atmosphere Model (CAM4) in forced SST and fully coupled experiments. *J. Climate* **26**, 5150–5168 (2013).
68. R. B. Neale, A. Gettelman, S. Park, C.-C. Chen, P. H. Lauritzen, D. L. Williamson, A. J. Conley, D. Kinnison, D. Marsh, A. K. Smith, F. Vitt, R. Garcia, J.-F. Lamarque, M. Mills, S. Tilmes, H. Morrison, P. Cameron-Smith, W. D. Collins, M. J. Iacono, R. C. Easter, X. Liu, S. J. Ghan, P. J. Rasch, M. A. Taylor, Description of the NCAR Community Atmosphere Model (CAM 5.0) (NCAR/TN-486+STR) (2012); https://doi.org/10.5065/wgtk-4g06.
69. G. Danabasoglu, J. -F. Lamarque, J. Bacmeister, D. A. Bailey, A. K. DuVivier, J. Edwards, L. K. Emmons, J. Fasullo, R. Garcia, A. Gettelman, C. Hannay, M. M. Holland, W. G. Large, P. H. Lauritzen, D. M. Lawrence, J. T. M. Lenaerts, K. Lindsay, W. H. Lipscomb, M. J. Mills, R. Neale, K. W. Oleson, B. Otto-Bliesner, A. S. Phillips, W. Sacks, S. Tilmes, L. Kampenhout, M. Vertenstein, A. Bertini, J. Dennis, C. Deser, C. Fischer, B. Fox-Kemper, J. E. Kay, D. Kinnison, P. J. Kushner, V. E. Larson, M. C. Long, S. Mickelson, J. K. Moore, E. Nienhouse, L. Polvani, P. J. Rasch, W. G. Strand, The Community Earth System Model version 2 (CESM2). *J. Adv. Model. Earth Syst.* **12**, e2019MS001916 (2020).
70. K. D. Williams, D. Copsey, E. W. Blockley, A. Bodas-Salcedo, D. Calvert, R. Comer, P. Davis, T. Graham, H. T. Hewitt, R. Hill, P. Hyder, S. Ineson, T. C. Johns, A. B. Keen, R. W. Lee, A. Megann, S. F. Milton, J. G. L. Rae, M. J. Roberts, A. A. Scaife, R. Schiemann, D. Storkey, L. Thorpe, I. G. Watterson, D. N. Walters, A. West, R. A. Wood, T. Woollings, P. K. Xavier, The Met Office Global Coupled model 3.0 and 3.1 (GC3.0 and GC3.1) configurations. *J. Adv. Model. Earth Syst.* **10**, 357–380 (2017).

71. I. M. Held, H. Guo, A. Adcroft, J. P. Dunne, L. W. Horowitz, J. Krasting, E. Shevliakova, M. Winton, M. Zhao, M. Bushuk, A. T. Wittenberg, B. Wyman, B. Xiang, R. Zhang, W. Anderson, V. Balaji, L. Donner, K. Dunne, J. Durachta, P. P. G. Gauthier, P. Ginoux, J. -C. Golaz, S. M. Griffies, R. Hallberg, L. Harris, M. Harrison, W. Hurlin, J. John, P. Lin, S. -J. Lin, S. Malyshev, R. Menzel, P. C. D. Milly, Y. Ming, V. Naik, D. Paynter, F. Paulot, V. Ramaswamy, B. Reichl, T. Robinson, A. Rosati, C. Seman, L. G. Silvers, S. Underwood, N. Zadeh, Structure and performance of GFDL's CM4.0 climate model. *J. Adv. Model. Earth Syst.* **11**, 3691–3727 (2019).

72. M. A. A. Rugenstein, K. C. Armour, Three flavors of radiative feedbacks and their implications for estimating equilibrium climate sensitivity. *Geophys. Res. Lett.* **48**, e2021GL092983 (2021).

73. J. M. Gregory, W. J. Ingram, M. A. Palmer, G. S. Jones, P. A. Stott, R. B. Thorpe, J. A. Lowe, T. C. Johns, K. D. Williams, A new method for diagnosing radiative forcing and climate sensitivity. *Geophys. Res. Lett.* **31**, L03205 (2004).

74. J. F. Adkins, K. McIntyre, D. P. Schrag, The salinity, temperature, and δ18O of the glacial deep ocean. *Science* **298**, 1769–1773 (2002).

75. R. A. Green, L. Menviel, K. J. Meissner, X. Crosta, D. Chandan, G. Lohmann, W. R. Peltier, X. Shi, J. Zhu, Evaluating seasonal sea-ice cover over the Southern Ocean at the Last Glacial Maximum. *Clim. Past* **18**, 845–862 (2022).

76. K. M. Shell, J. T. Kiehl, C. A. Shields, Using the radiative kernel technique to calculate climate feedbacks in NCAR's Community Atmospheric Model. *J. Climate* **21**, 2269–2282 (2008).

77. A. G. Pendergrass, apendergrass/cam5-kernels (2019);
https://doi.org/10.5281/zenodo.3359041.
78. M. Webb, Code and Data for WCRP Climate Sensitivity Assessment (2020);
https://doi.org/10.5281/zenodo.3945275.
79. J. F. Kok, T. Storelvmo, V. A. Karydis, A. A. Adebiyi, N. M. Mahowald, A. T. Evan, C. He, D. M. Leung, Mineral dust aerosol impacts on global climate and climate change. *Nat. Rev. Earth Environ.* **4**, 71–86 (2023).
80. N. M. Mahowald, L. Li, S. Albani, D. S. Hamilton, J. F. Kok, Opinion: The importance of historical and paleoclimate aerosol radiative effects. *Atmos. Chem. Phys.* **24**, 533–551 (2024).
81. N. Sagoo, T. Storelvmo, Testing the sensitivity of past climates to the indirect effects of dust. *Geophys. Res. Lett.* **44**, 5807–5817 (2017).
82. S. Albani, N. M. Mahowald, Paleodust insights into dust impacts on climate. *J. Climate* **32**, 7897–7913 (2019).
83. S. Albani, Y. Balkanski, N. Mahowald, G. Winckler, V. Maggi, B. Delmonte, Aerosol-climate interactions during the Last Glacial Maximum. *Curr. Clim. Change Rep.* **4**, 99–114 (2018).
84. I. C. Prentice, S. P. Harrison, P. J. Bartlein, Global vegetation and terrestrial carbon cycle changes after the last ice age. *New Phytol.* **189**, 988–998 (2011).
85. P. J. Bartlein, S. P. Harrison, S. Brewer, S. Connor, B. A. S. Davis, K. Gajewski, J. Guiot, T. I. Harrison-Prentice, A. Henderson, O. Peyron, I. C. Prentice, M. Scholze, H. Seppä, B. Shuman, S. Sugita, R. S. Thompson, A. E. Viau, J. Williams, H. Wu, Pollen-based continental climate reconstructions at 6 and 21 ka: A global synthesis. *Clim. Dyn.* **37**, 775–802 (2011).

86. M. Kageyama, S. Albani, P. Braconnot, S. P. Harrison, P. O. Hopcroft, R. F. Ivanovic, F. Lambert, O. Marti, W. R. Peltier, J.-Y. Peterschmitt, D. M. Roche, L. Tarasov, X. Zhang, E. C. Brady, A. M. Haywood, A. N. LeGrande, D. J. Lunt, N. M. Mahowald, U. Mikolajewicz, K. H. Nisancioglu, B. L. Otto-Bliesner, H. Renssen, R. A. Tomas, Q. Zhang, A. Abe-Ouchi, P. J. Bartlein, J. Cao, Q. Li, G. Lohmann, R. Ohgaito, X. Shi, E. Volodin, K. Yoshida, X. Zhang, W. Zheng, The PMIP4 contribution to CMIP6 – Part 4: Scientific objectives and experimental design of the PMIP4-CMIP6 Last Glacial Maximum experiments and PMIP4 sensitivity experiments. *Geosci. Model Dev.* **10**, 4035–4055 (2017).
87. G. A. Schmidt, J. D. Annan, P. J. Bartlein, B. I. Cook, E. Guilyardi, J. C. Hargreaves, S. P. Harrison, M. Kageyama, A. N. LeGrande, B. Konecky, S. Lovejoy, M. E. Mann, V. Masson-Delmotte, C. Risi, D. Thompson, A. Timmermann, L.-B. Tremblay, P. Yiou, Using palaeo-climate comparisons to constrain future projections in CMIP5. *Clim. Past* **10**, 221–250 (2014).
88. J. Zhu, C. J. Poulsen, J. E. Tierney, Simulation of Eocene extreme warmth and high climate sensitivity through cloud feedbacks. *Sci. Adv.* **5**, eaax1874 (2019).
89. W. R. Peltier, D. F. Argus, R. Drummond, Space geodesy constrains ice age terminal deglaciation: The global ICE-6G-C (VM5a) model. *J Geophys Res Solid Earth* **120**, 450–487 (2014).
90. D. F. Argus, W. R. Peltier, R. Drummond, A. W. Moore, The Antarctica component of postglacial rebound model ICE-6G_C (VM5a) based on GPS positioning, exposure age dating of ice thicknesses, and relative sea level histories. *Geophys. J. Int.* **198**, 537–563 (2014).
91. P. N. DiNezio, J. E. Tierney, The effect of sea level on glacial Indo-Pacific climate. *Nat. Geosci.* **6**, 485–491 (2013).

92. J. W. Hurrell, J. J. Hack, D. Shea, J. M. Caron, J. Rosinski, A new sea surface temperature and sea ice boundary dataset for the community atmosphere model. *J. Clim.* **21**, 5145–5153 (2008).

93. P. R. Gent, G. Danabasoglu, L. J. Donner, M. M. Holland, E. C. Hunke, S. R. Jayne, D. M. Lawrence, R. B. Neale, P. J. Rasch, M. Vertenstein, P. H. Worley, Z.-L. Yang, M. Zhang, The Community Climate System Model version 4. *J. Climate* **24**, 4973–4991 (2011).

94. A. Voldoire, D. Saint-Martin, S. Sénési, B. Decharme, A. Alias, M. Chevallier, J. Colin, J. -F. Guérémy, M. Michou, M. -P. Moine, P. Nabat, R. Roehrig, D. Salas y Mélia, R. Séférian, S. Valcke, I. Beau, S. Belamari, S. Berthet, C. Cassou, J. Cattiaux, J. Deshayes, H. Douville, C. Ethé, L. Franchistéguy, O. Geoffroy, C. Lévy, G. Madec, Y. Meurdesoif, R. Msadek, A. Ribes, E. Sanchez-Gomez, L. Terray, R. Waldman, Evaluation of CMIP6 DECK experiments with CNRM-CM6-1. *J. Adv. Model. Earth Syst.* **11**, 2177–2213 (2019).

95. P. M. Cox, R. A. Betts, C. D. Jones, S. A. Spall, I. J. Totterdell, Acceleration of global warming due to carbon-cycle feedbacks in a coupled climate model. *Nature* **408**, 184–187 (2000).

96. T. Mauritsen, J. Bader, T. Becker, J. Behrens, M. Bittner, R. Brokopf, V. Brovkin, M. Claussen, T. Crueger, M. Esch, I. Fast, S. Fiedler, D. Fläschner, V. Gayler, M. Giorgetta, D. S. Goll, H. Haak, S. Hagemann, C. Hedemann, C. Hohenegger, T. Ilyina, T. Jahns, D. Jimenéz-de-la-Cuesta, J. Jungclaus, T. Kleinen, S. Kloster, D. Kracher, S. Kinne, D. Kleberg, G. Lasslop, L. Kornblueh, J. Marotzke, D. Matei, K. Meraner, U. Mikolajewicz, K. Modali, B. Möbis, W. A. Müller, J. E. M. S. Nabel, C. C. W. Nam, D. Notz, S.-S. Nyawira, H. Paulsen, K. Peters, R. Pincus, H. Pohlmann, J. Pongratz, M. Popp, T. J. Raddatz, S. Rast, R. Redler, C. H. Reick, T. Rohrschneider, V. Schemann, H. Schmidt, R. Schnur, U. Schulzweida, K. D. Six, L. Stein, I.

Stemmler, B. Stevens, J.-S. von Storch, F. Tian, A. Voigt, P. Vrese, K.-H. Wieners, S. Wilkenskjeld, A. Winkler, E. Roeckner, Developments in the MPI-M Earth System Model version 1.2 (MPI-ESM1.2) and its response to increasing CO2. *J. Adv. Model. Earth Syst.* **11**, 998–1038 (2019).

97. D. Paynter, T. L. Frölicher, L. W. Horowitz, L. G. Silvers, Equilibrium climate sensitivity obtained from multimillennial runs of two GFDL climate models. *J. Geophys. Res. Atmos.* **123**, 1921–1941 (2018).

98. K-1 Model Developers, K-1 Coupled GCM (MIROC) Description (2004).

99. A. Yamamoto, A. Abe-Ouchi, M. Shigemitsu, A. Oka, K. Takahashi, R. Ohgaito, Y. Yamanaka, Global deep ocean oxygenation by enhanced ventilation in the Southern Ocean under long-term global warming. *Global Biogeochem. Cycles* **29**, 1801–1815 (2015).

100. A. G. Pendergrass, A. Conley, F. M. Vitt, Surface and top-of-atmosphere radiative feedback kernels for CESM-CAM5. *Earth Syst. Sci. Data* **10**, 317–324 (2018).

101. M. D. Zelinka, T. A. Myers, D. T. McCoy, S. Po-Chedley, P. M. Caldwell, P. Ceppi, S. A. Klein, K. E. Taylor, Causes of higher climate sensitivity in CMIP6 models. *Geophys. Res. Lett.* **47**, e2019GL085782 (2020).

102. B. J. Soden, I. M. Held, An assessment of climate feedbacks in coupled ocean–atmosphere models. *J. Clim.* **19**, 3354–3360 (2006).
